# Copper-Catalyzed One-Pot Functionalization of Styrenes: Application toward Pharmaceutically Relevant Phenylethylamine Triazole Scaffolds

**DOI:** 10.1021/acs.joc.5c01782

**Published:** 2025-09-15

**Authors:** Timothy A. Hilton, Thomas M. Richardson, Aidan P. McKay, Allan J. B. Watson

**Affiliations:** EaStCHEM, School of Chemistry, University of St Andrews, St Andrews, Fife KY16 9ST, United Kingdom

## Abstract

A Cu-catalyzed, one-pot synthesis of 1,2,3-triazolyl phenylethylamine scaffolds is reported. This method utilizes a single copper catalyst to promote aziridination via nitrene addition, ring-opening with azide, and copper-catalyzed azide–alkyne cycloaddition to afford 1,4-disubstituted 1,2,3-triazole phenylethylamine scaffolds. The utility of this process is demonstrated in the synthesis of pharmaceutically relevant cores from simple styrene motifs.

## Graphical abstract

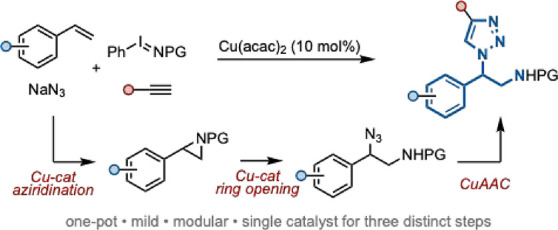

Aryl and heteroaryl ethylamine scaffolds are ubiquitous within bioactive molecules and natural products.^1^ Aryl triazole moieties have similarly found widespread utility across materials science^2^ and chemical biology.^3^ These scaffolds have also been utilized extensively within drug development, with examples such as centhaquine and rufinamide (Scheme 1a), showing promise as treatments for hypovolemic shock and as seizure medication, respectively.^1,4^

**Scheme 1:**
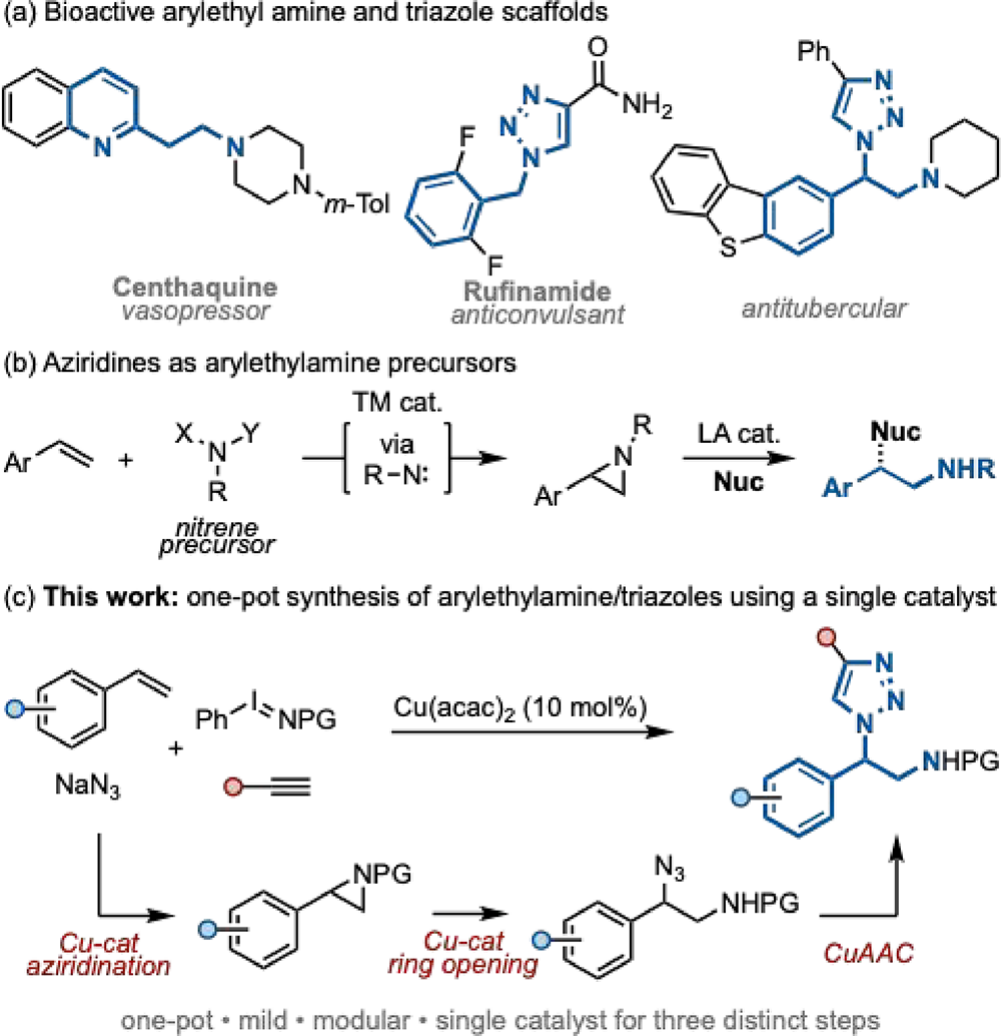
Access to (Hetero)­aryl Ethylamine Scaffolds Using Aziridine Intermediates

Numerous methodologies to access arylethylamines have been reported. Recently, the application of aziridine scaffolds as precursors for aryl ethylamine cores has become more prominent.^5^ Aziridines are readily accessed from alkenes,^6^ employing metal-nitrene chemistry using a range of nitrene precursors,^6,7^ catalysts,^7^ ^b‑e^,^8^ and chiral ligands.^9^ Subsequent aziridine ring-opening by a variety of nucleophiles, often promoted by Lewis acid catalysts, gives access to C–C, C–N, C–O, C–S, and C–X ethylamine scaffolds (Scheme 1b).^10^

The copper-catalyzed azide–alkyne cycloaddition (CuAAC) reaction is a robust methodology for the construction of 1,2,3-triazole motifs (Scheme 1c)^11^; however, the direct functionalization of alkenes to aminoazide motifs for use in CuAAC processes remains relatively rare. Recent methodologies have been reported by Morandi and co-workers,^12^ Che and co-workers,^13^ and Aikawa and co-workers,^14^ utilizing Fe, Ru, and Cu catalysis, respectively. The applicability of these products within CuAAC chemistry to afford 1,2,3-triazole scaffolds has been demonstrated from the isolated aminoazide species.^12–14^ Phenylethylamine triazole scaffolds were previously accessed from activated aryl aziridines.

Kumaraswamy et al. reported the ring opening of an *N*-Ts aryl aziridine with NaN_3_ to afford the corresponding aminoazide in situ, with subsequent CuAAC to deliver the triazole product.^15^ A similar process was reported by Xia and co-workers using Ru photocatalysis.^16^ Tokuyama and co-workers reported the direct synthesis of aminoazides from styrene under Cu catalysis without CuAAC.^17^ Moran and Lebœuf have also described the 1,2-diamination of styrenes via the ring-opening of an aziridine intermediate under Fe catalysis.^18^

The mechanistic basis for the Cu-catalyzed aziridination,^19^ Cu-catalyzed aziridine ring-opening,^20^ and CuAAC^21^ is well established. With this in mind, we considered that a single Cu catalyst could be used sequentially for multiple roles, enabling a one-pot approach to access phenylethylamine 1,2,3-triazole scaffolds from styrenes (Scheme 1c). Such multicatalysis platforms are emerging as attractive methods for the synthesis of more complex, value-added products from simple starting materials.^22^ This system would offer improved reaction efficiency, while also removing the need for isolation of reaction intermediates, in a similar manner to other multistep sequential processes, which have been reported using single Pd,^23^ Rh,^24^ and Cu catalysts.^25^

Optimization of the individual aziridination, ring-opening, and CuAAC steps were conducted separately (see ESI for full details): a brief overview selected variables is shown in Table 1 (see ESI for full details).

**Table 1:**
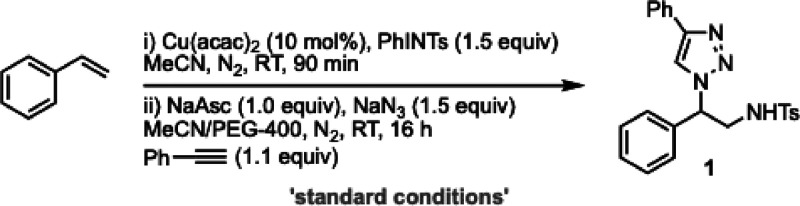
Reaction Development^a^

entry	deviation from “standard conditions”	yield **1** (%)^b^
1	none	88^c^
2	CuBr_2_	68
3	Cu(TFA)_2_	50
4	MeCN:water (1:1)	20
5	No Cu(acac)_2_	0
6	No NaAsc	47

a Reactions performed at a 0.30 mmol scale. NaAsc = sodium ascorbate; PEG–400 = polyethylene glycol 400.

b Determined by ^1^H NMR using MeNO_2_ as an internal standard.

c Isolated yield.

Mild conditions using Cu­(acac)_2_ delivered the desired phenylethylamine triazole scaffold (**1**) in 88% isolated yield as a single regioisomer (entry 1). Alternative Cu sources could be employed with erosion in yield (entries 2 and 3). The use of PEG-400 as a cosolvent, to solubilize NaN_3_, was found to be necessary for a successful reaction in agreement with observations noted by Kumaraswamy and co-workers, with concentration effects noted (see ESI for full details).^15^ Using water as an alternative cosolvent significantly decreased the product yield (entry 4). Control reactions confirmed that the reaction cannot proceed in the absence of a catalyst (entry 5). The reaction was significantly improved by the presence of NaAsc; however, it was observed to proceed in the absence of an external reductant (entry 6), suggesting that the resting state of the catalyst after the aziridination step is Cu­(I), in agreement with previous mechanistic studies of these systems.^19^

The generality of the developed process was explored by using a range of styrenes, acetylenes, and iminoiodanes (Scheme 2). A wide range of substituted styrene derivatives were accommodated, with generally moderate-to-excellent yields observed. These included examples of synthetic handles (**2**) and electronically distinct (**4**, **5**) substituents.

**Scheme 2:**
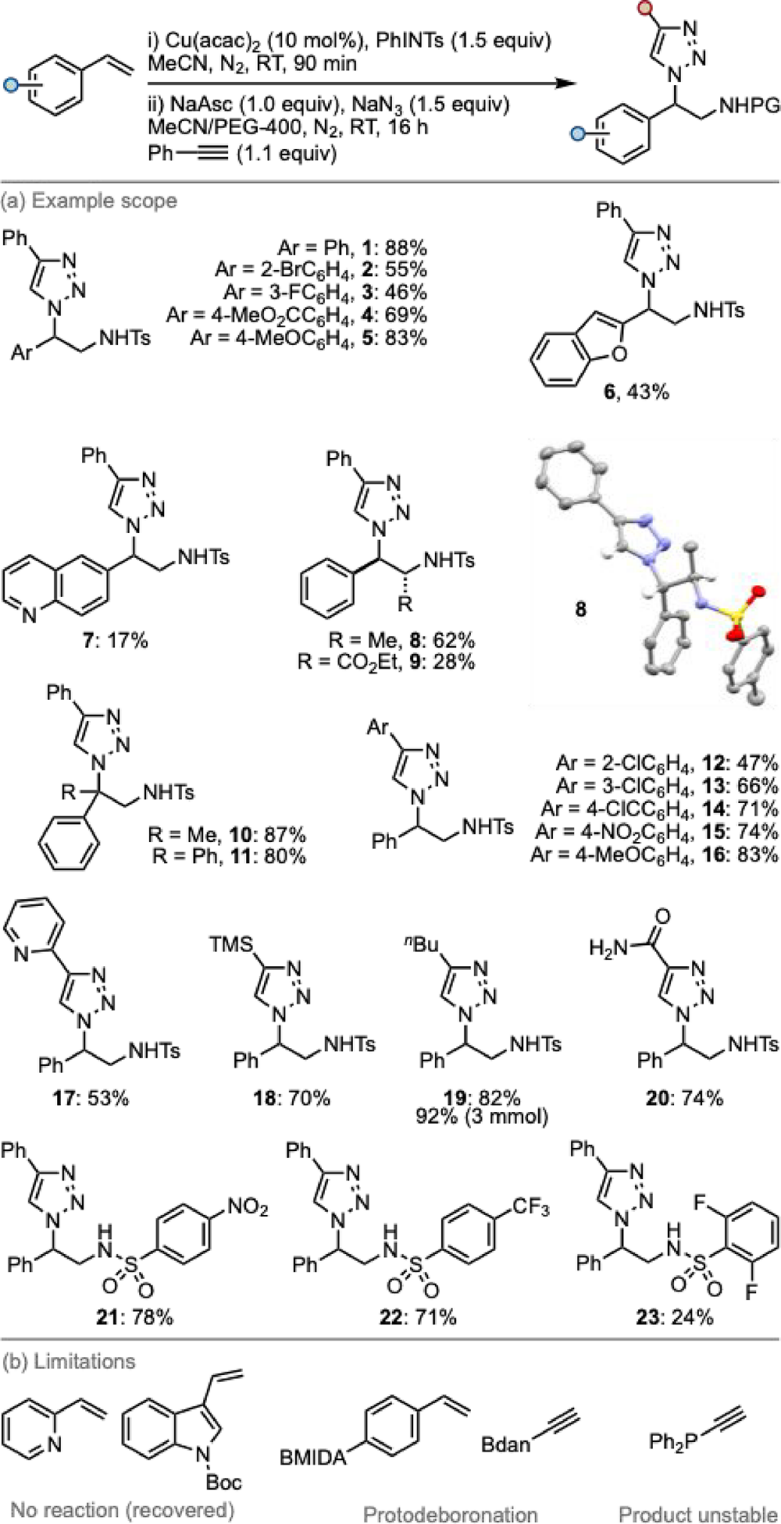
Reaction Scope^a^

a Reactions performed at a 0.30 mmol scale, unless otherwise stated. Isolated yields.

Heterocyclic olefins were tolerated, although with significantly reduced yields (**6**, **7**). β-Substituted styrenes were tolerated, giving the expected products as a single diastereomer (**8**, **9**), enabling access to unnatural amino acid derivatives. The reaction was also amenable to substitution at the benzylic position (**10**, **11**).

Consistent with classic CuAAC methodologies, a broad variety of acetylenes could be employed without significant erosion in yield. This included substituted phenyl acetylene derivatives (**12–16**) and acetylenes with heterocyclic (**17**), silyl (**18**), alkyl (**19**), and acyl (**20**) groups, delivering the desired triazoles in high yield. Reaction scale-up was also successfully demonstrated, with **19** delivered in excellent yield at the gram scale.

Finally, alternative iminoiodanes were explored as substrates. Nosyl-protected substrate **21** was obtained in good yield, providing a smoother deprotection to the free amine scaffolds than tosyl derivatives (e.g., **1**).^26^ Alternative electron-deficient derivatives were obtained in moderate to good yield (**22, 23**).

With regard to limitations, specific heterocyclic alkenes, such as vinylpyridine and *N*-boc-3-vinyl indole, were unreactive and recovered almost quantitatively from the reaction mixtures, suggesting an issue with the aziridination step. When using a protected *N*-methyliminodiacetyl boronate (BMIDA) substituted styrene or a naphthalene-1,8-diamino boryl (BDan) acetylene, protodeboronation occurred during the reaction. The product from the reaction of diphenylphosphinyl acetylene was not stable to isolation.

Finally, we sought to apply the developed methodology to a range of pharmaceutically relevant cores, giving derivatives of triazolylamines that have shown promising bioactivity (Scheme 3). Kantevari and co-workers have disclosed the antitubercular activity of ethylamine dibenzo­[*b*,*d*]­thiophene-1,2,3-triazole derivatives.^27^ Using the described one-pot process, these motifs could be accessed in a streamlined fashion (**24**). Rufinamide analogue **25** was obtained from 2,6-difluorostyrene.^28^ A derivative of trimethoxybenzamide was obtained from 3,4,5-trimethoxystyrene (**26**).^29^ Finally, **27**, an analogue of carboxyamidotriazole, was obtained from 3,5-dichloro-4-carbomethoxystyrene.^30^

**Scheme 3:**
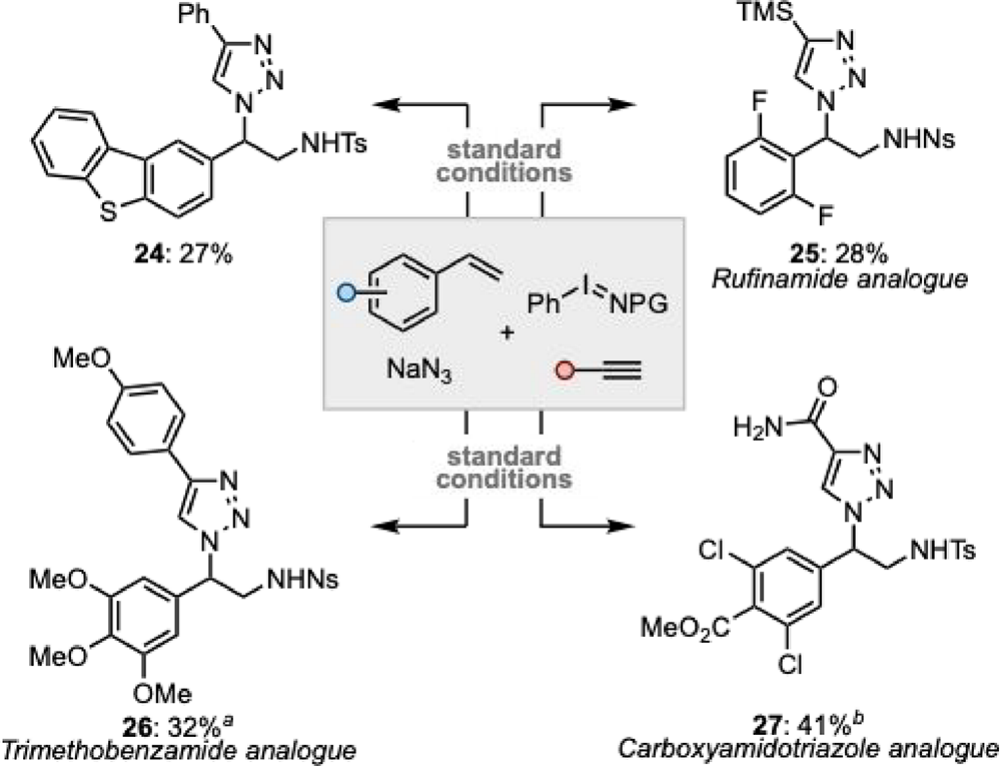
Access to Pharmaceutical Cores^a^

a Reactions performed at a 0.30 mmol scale. Isolated yields. ^a^0.60 mmol scale. ^b^0.20 mmol scale.

In summary, a copper-catalyzed, one-pot functionalization of (hetero)­aryl alkene scaffolds to 1,2,3-triazolyl aryl ethylamines has been developed. The reaction uses a single catalyst for three distinct reactions, proceeds under mild conditions, and allows the modular and efficient construction of triazole ethylamine cores with utility in a variety of therapeutic areas.

## Supplementary Material



## Data Availability

The data underlying this study are available in the published article and its Supporting Information.

## Notes

Reaction optimization, compound characterization data, crystal structure data, and NMR spectra (PDF)
