# Supplementary material for: Copper-Catalyzed One-Pot Functionalization of Styrenes: Application toward Pharmaceutically Relevant Phenylethylamine Triazole Scaffolds
Source: J Org Chem. 2025 Sep 15;90(38):13774–8. doi: 10.1021/acs.joc.5c01782 (PMC12481559; doi:10.1021/acs.joc.5c01782)

Supporting Information

Copper-Catalyzed One-Pot Functionalization of Styrenes: Application towards Pharmaceutically Relevant Phenylethylamine Triazole Scaffolds

Timothy A. Hilton, Thomas M. Richardson, Aidan P. McKay, and Allan J. B. Watson*

EaStCHEM, School of Chemistry, University of St Andrews, North Haugh, St Andrews, Fife, KY16 9ST, UK

*Email: aw260@st-andrews.ac.uk

Contents:

1. General Experimental Details S2
2. General Experimental Procedures S3
3. Reaction Optimisation Data S4
4. Characterisation Data S10
5. X-Ray Crystallography S31
6. References S34
7. Copies of NMR Spectra S36

1. General Experimental Details

1.1. Purification of Solvents and Reagents

Dry MeCN was obtained by distillation of acetonitrile (HPLC grade, Fischer) into a flame dried flask containing activated 4Å molecular sieves under a blanket of N_2. Dry THF was obtained from a PureSolv SPS-400-5 solvent purification system. Acetone, Et_2O, EtOAc, and hexane for purification purposes were used as obtained from suppliers without further purification. All other reagents and solvents were obtained from commercial suppliers and were used without further purification unless otherwise stated.

1.2. Experimental Details

Reactions were carried out using conventional glassware, in 4 mL dram vials, or in capped 10 mL microwave vials. Microwave vials were oven dried (180 °C) and cooled to room temperature under vacuum prior to use. Reaction mixtures were prepared in vials before being capped with a septum and placed under an atmosphere of N_2 by evacuating and refilling with N_2 (three cycles). Reactions carried out at elevated temperatures were performed using a sand bath (sand bath temperature) or oil bath (oil temperature) atop a temperature-regulated hotplate/stirrer. Cooling to 0 °C was achieved using an ice/water bath.

1.3. Purification of Products

Thin layer chromatography was performed using Merck silica plates coated with fluorescent indicator UV254. These were analysed under 254 nm UV light and/or developed using aqueous potassium permanganate or ethanolic vanillin solution. Flash column chromatography was performed using silica gel (40–62 μm, Fluorochem).

1.4. Analysis of Products

^{1}H and ^{13}C NMR spectra were obtained on either a Brucker AVII 400 (fitted with a BBFO probe), or Bruker Neo 400 (fitted with a BBF-H-D-iprobe) at 400 and 101 MHz, respectively; or on a Bruker AVIII 500 (fitted with either a CryoProbe Prodigy BBO, or SmartProbe BBFO+ probe) at 500 and 128 MHz, respectively. ^{19}F NMR were obtained on a Bruker AVIII-HD 500 (fitted with a SmartProbe BBFO+ probe) at 470 MHz, or on a Bruker Neo 400 (fitted with a BBF-H-D-iprobe) at 376 MHz. All spectra were recorded at room temperature, with the deuterated solvents used as a lock for spectra and internal reference ($CDCl_3$: ^{1}H, 7.26 ppm and ^{13}C, 77.16 ppm; Acetone-d_6: ^{1}H, 2.05 ppm and ^{13}C 28.9 ppm, 206.3 ppm; DMSO-d_6: ^{1}H, 2.50 ppm and ^{13}C, 39.5 ppm). Unless otherwise stated, ^{13}C and ^{19}F NMR spectra were measured proton-decoupled. All chemical shifts (δ) are reported in parts per million (ppm). All coupling constants, J, are quoted in Hz, and refer to $^{3}J_{HH}$ unless otherwise noted. NMR spectra are reported as follows: chemical shift/ppm (multiplicity, coupling constant(s), number of nuclei). Multiplicity is given as broad (br.), singlet (s), doublet (d), triplet (t), quartet (q), quintet (quint), hextet (h), multiplet (m), and combinations thereof. Signals which overlap are reported as multiplets. Fourier Transformed Infra-Red (FTIR) spectra were obtained using a Shimadzu IRAffinity-1 Fourier transform IR spectrophotometer with a Specac Aquest ATR (diamond puck). Spectra were recorded as specified within the procedure as solids, oils, or thin films (CH_2Cl_2 or acetone). Transmittance is recorded as maximal absorption in wavenumbers (cm^{-1}). High resolution mass spectrometry (HRMS) were recorded on a Bruker micrOTOF benchtop ESI with either positive or negative electrospray ionisation or EI using a Thermo Mat 900XP, Double Focussing Hi-resolution mass spectrometer at the University of Edinburgh mass spectrometry facility (SIRCAMS). The number of decimal places is determined by the accuracy of the machine (*i.e.,* both four and five digits given).

2. General Experimental Procedures

2.1. General Procedure A

One-pot procedure, for example synthesis of **1**.

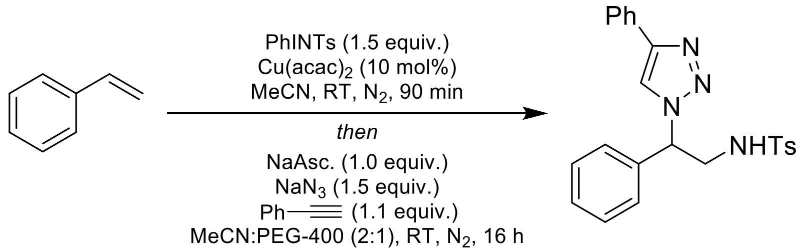

A 4 mL dram vial equipped with a Teflon-coated stir-bar was charged with iminoiodinane (0.46 mmol, 1.5 equiv.) and styrene derivative (0.30 mmol, 1.0 equiv. – **if solid**). The vial was capped and placed under an atmosphere of N_2 by evacuating and refilling with N_2 (three cycles). Dry MeCN (1.0 mL, 0.3 M) was added, followed by styrene derivative (0.30 mmol, 1.0 equiv. – **if liquid**). The reaction mixture was stirred for one minute at RT before the vial was quickly decapped, and copper(II) acetoacetonate (7.9 mg, 10 mol%) was added. The vial was recapped, and the reaction mixture was stirred at RT for 90 minutes under a flow of N_2.

An oven-dried microwave vial equipped with a Teflon-coated stir-bar was charged with sodium ascorbate (60.5 mg, 0.30 mmol, 1.0 equiv.), sodium azide (29.8 mg, 0.46 mmol, 1.5 equiv.) and alkyne (0.33 mmol, 1.1 equiv. – **if solid**). The vial was capped and placed under an atmosphere of N_2 by evacuating and refilling with N_2 (three cycles), before the addition of PEG-400 (500 µL, 0.6 M). The aziridine reaction mixture was added *via* syringe, and the resulting mixture was stirred at RT for two minutes prior to the addition of alkyne (0.33 mmol, 1.1 equiv. – **if liquid**). The reaction mixture was stirred at RT for 16 h, before being filtered through a short pad of silica gel (*c.a.* 2 cm), eluting with EtOAc (50 mL). The filtrate was washed with water (3 × 50 mL), dried over Na_2SO_4, filtered, and concentrated *in vacuo* to afford the crude product. This was purified by silica gel column chromatography, with respective purification methods disclosed below.

2.2. General Procedure B

Synthesis of iminoiodanes, for example PhINTs.

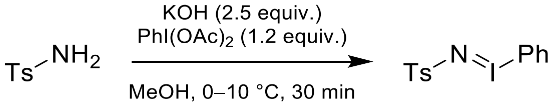

A round-bottom flask equipped with a Teflon-coated stir-bar was charged with 4-methylbenzenesulfonamide (6.85 g, 40 mmol, 1.0 equiv.), potassium hydroxide (5.55 g, 100 mmol, 2.5 equiv.), and MeOH (60 mL, 0.66 M). The resulting suspension was cooled in an ice-water bath, and phenyl-γ^3-iodanediyl diacetate (15.1 g, 48 mmol, 1.2 equiv.) was added portionwise over a period of 10–20 minutes, such that the internal temperature was maintained below 10 °C. The reaction mixture was stirred for 30 minutes in an ice-water bath, before being refrigerated (*c.a.* 5 °C) overnight. The resulting mixture was filtered, and the solid was washed with ice-cold MeOH (20 mL). The solid was dried *in vacuo* at 0.5 mbar to afford PhINTs as a pale-yellow solid (10.5 g, 70%).

2.3. General Procedure C

Synthesis of vinylic arenes by Suzuki coupling, for example 2-vinylbenzofuran.

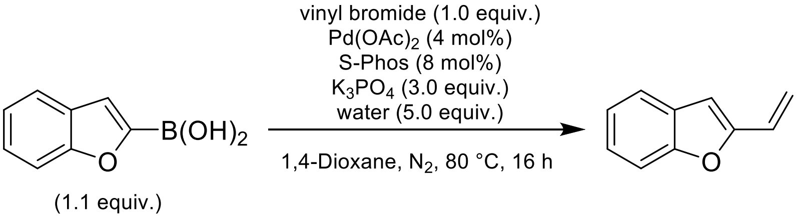

An oven-dried flask, equipped with a Teflon-coated stir-bar and a reflux condenser, was charged with benzofuran-2-ylboronic acid (356 mg, 2.2 mmol, 1.1 equiv.), palladium(II) acetate (18.0 mg, 80 µmol, 4 mol%), S-phos (65.7 mg, 160 µmol, 8 mol%), and K_3PO_4 (1.27 g, 6.0 mmol, 3.0 equiv.). The flask was sealed and placed under an atmosphere of N_2 by evacuating and refilling with N_2 (three cycles), before vinyl bromide (2.0 mL, 1.0 M in THF, 2.0 mmol, 1.0 equiv.), 1,4-dioxane (8.0 mL, 0.25 M), and water (180 µL, 10 mmol, 5.0 equiv.) were added, and the reaction mixture was stirred at 80 °C for 16 h. The mixture was allowed to cool to RT, and filtered through a short pad of celite, eluting with CH_2Cl_2 (50 mL). The mixture was concentrated *in vacuo*, and the crude product was purified by silica gel column chromatography, with respective purification methods disclosed below.

3. Reaction Optimisation Data

3.1. Optimisation of Aziridination step

General optimisation reaction procedure

An oven-dried 4 mL dram vial equipped with a Teflon-coated stir-bar was charged with PhINTs (171 mg, 0.46 mmol, 1.5 equiv.) The vial was capped and placed under an atmosphere of N_2 by evacuating and refilling with N_2 (three cycles). Styrene (36.0 µL, 0.30 mmol, 1.0 equiv.) was added, followed by dry MeCN (3 mL, 0.1 M). The vial was quickly decapped and $Cu(acac)_2$ (7.9 mg, 10 mol%) was added. The vial was recapped and the reaction mixture was stirred at RT for two hours under a flow of N_2. The reaction was poured into a mixture of brine and 10% aq. NH_3 solution (1:1, 5 mL). The reaction was extracted with EtOAc (3 × 5 mL), and the combined organic extracts were dried over Na_2SO_4, filtered, and concentrated *in vacuo*. NMR yield was quantified by ^{1}H NMR analysis of the crude reaction mixture using trichloroethylene (TCE, 27.0 µL, 0.30 mmol, 1.0 equiv.) as an internal standard.

Table S1: Reaction stoichiometry screen

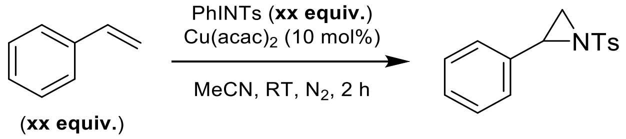

Entry	Equiv. styrene	Equiv. PhINTs	Aziridine (%)[a]	Styrene (%)[a]
1	5.0	1.0	75	17
2	4.0	1.0	69[b]	-
3	2.0	1.0	62	25
4	1.0	2.0	84 (76)[b]	8
**5**	**1.0**	**1.5**	**Quant. (99)[b]**	**0**

Reaction conditions: MeCN [0.1 M], RT, N_2, 2 h. [a] Determined by ^{1}H NMR using TCE as internal standard; [b] Isolated yield.

Table S2: Copper catalyst screen

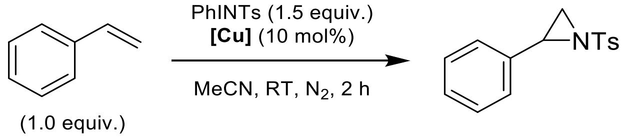

Entry	[Cu]	Aziridine (%)[a]	Styrene (%)[a]
**1**	**$Cu(acac)_2$**	**Quant.**	**0**
2	$Cu(OTf)_2$	75	0
3	$Cu(OAc)_2$	10	26
4	$CuSO_4$	0	76
**5**	**$Cu(TFA)_2$**	**Quant.**	**0**
6	$CuBr_2$	85	0

Reaction conditions: MeCN [0.1 M], RT, N_2, 2 h. [a] Determined by ^{1}H NMR using TCE as internal standard.

Table S3: Concentration screen (2-hour reaction time)

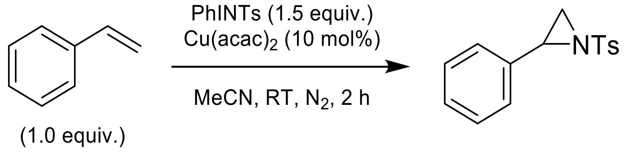

Entry	Reaction concentration	Aziridine (%)[a]	Styrene (%)[a]
1	0.025 M	95	0
2	0.050 M	Quant.	0
3	0.100 M	Quant.	0
4	0.200 M	Quant.	0
5	0.400 M	Quant.	0

[a] Determined by ^{1}H NMR using TCE as internal standard.

Table S4: Concentration screen (30-minute reaction time)

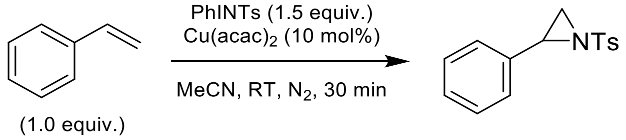

Entry	Reaction concentration	Aziridine (%)[a]	Styrene (%)[a]
1	0.025 M	81	5
2	0.050 M	89	10
**3**	**0.100 M**	**Quant.**	**0**
**4**	**0.200 M**	**95**	**0**
**5**	**0.400 M**	**98**	**0**

[a] Determined by ^{1}H NMR using TCE as internal standard.

3.2. Optimisation of Ring opening/click step

<u>Prepared according to the procedure outlined by Kumaraswamy.</u>[1]

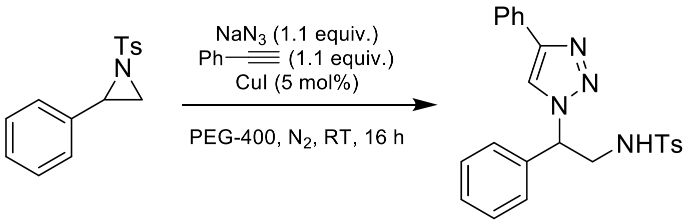

82%

An oven-dried microwave vial equipped with a Teflon-coated stir-bar was charged with 2-phenyl-1-tosylaziridine (54.7 mg, 0.20 mmol, 1.0 equiv.), sodium azide (14.3 mg, 0.22 mmol, 1.1 equiv.), and CuI (1.9 mg, 5 mol%). The vial was capped and placed under an atmosphere of N_2 by evacuating and refilling with N_2 (three cycles). PEG–400 (600 µL, 0.3 M) was added, and the reaction mixture was stirred for five minutes at RT prior to the addition of phenylacetylene (21.0 µL, 0.22 mmol, 1.1 equiv.), and the reaction mixture was stirred at RT for 16 hours. The reaction mixture was quenched with water (5 mL) and extracted with EtOAc (3 × 10 mL). The combined organic extracts were dried over Na_2SO_4, filtered, and concentrated *in vacuo*. The crude product was purified by flash column chromatography (silica gel, 40% EtOAc in hexane) to afford the product as a white amorphous solid (68.4 mg, 82%).

<u>Using alternative *t*BuOH/water solvent conditions.</u>

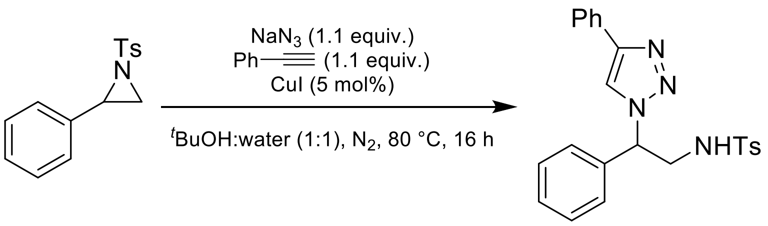

76%

An oven-dried microwave vial equipped with a Teflon-coated stir-bar was charged with 2-phenyl-1-tosylaziridine (54.7 mg, 0.20 mmol, 1.0 equiv.), sodium azide (14.3 mg, 0.22 mmol, 1.1 equiv.), and CuI (1.9 mg, 5 mol%). The vial was capped and placed under an atmosphere of N_2 by evacuating and refilling with N_2 (three cycles). A 1:1 mixture of water and *tert*-butanol (600 µL, 0.33 M) was added

and the reaction mixture was stirred for five minutes at RT prior to the addition of phenylacetylene (21.0 µL, 0.22 mmol, 1.1 equiv.), and the reaction mixture was stirred at 80 °C for 16 hours. The reaction mixture was quenched by addition of water (5 mL) and extracted with EtOAc (3 × 10 mL). The combined organic extracts were dried over Na_2SO_4, filtered, and concentrated *in vacuo*. NMR yield was quantified by ^{1}H NMR analysis of the crude reaction mixture using trichloroethylene (TCE, 18.0 µL, 0.20 mmol, 1.0 equiv.) as an internal standard (76% NMR yield).

Using alternative MeCN:PEG–400 solvent conditions.

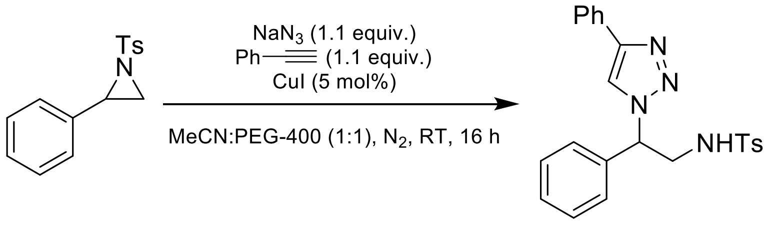

An oven-dried microwave vial equipped with a Teflon-coated stir-bar was charged with 2-phenyl-1-tosylaziridine (54.7 mg, 0.20 mmol, 1.0 equiv.), sodium azide (14.3 mg, 0.22 mmol, 1.1 equiv.), and CuI (1.9 mg, 5 mol%). The vial was capped and placed under an atmosphere of N_2 by evacuating and refilling with N_2 (three cycles). MeCN (300 µL, 0.66 M) and PEG–400 (300 µL, 0.66 M) were added and the reaction mixture was stirred for five minutes at RT prior to the addition of phenylacetylene (21.0 µL, 0.22 mmol, 1.1 equiv.), and the reaction mixture was stirred at RT for 16 hours. The reaction mixture was quenched with water (5 mL) and extracted with EtOAc (3 × 10 mL). The combined organic extracts were dried over Na_2SO_4, filtered, and concentrated *in vacuo*. NMR yield was quantified by ^{1}H NMR analysis of the crude reaction mixture using trichloroethylene (TCE, 18.0 µL, 0.20 mmol, 1.0 equiv.) as an internal standard (83% NMR yield).

Evidence of copper-catalysis for the ring-opening

Table S5: Copper-catalyzed ring-opening screen

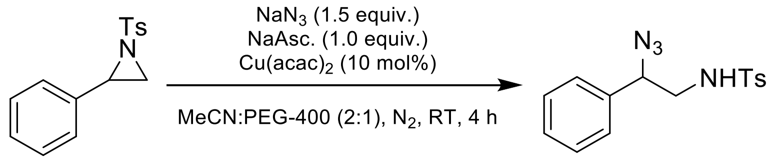

Entry	Change to conditions	Azide (%)[a]	Aziridine (%)[a]
1	none	57	39
2	no $Cu(acac)_2$	40	59

[a] Determined by ^{1}H NMR using TCE as internal standard.

An oven-dried microwave vial equipped with a Teflon-coated stir-bar was charged with 2-phenyl-1-tosylaziridine (27.3 mg, 0.10 mmol, 1.0 equiv.), sodium azide (9.9 mg, 0.15 mmol, 1.5 equiv.), sodium ascorbate (20.2 mg, 0.10 mmol, 1.0 equiv.), and $Cu(acac)_2$ (2.6 mg, 10 mol%). The vial was capped and placed under an atmosphere of N_2 by evacuating and refilling with N_2 (three cycles). MeCN (300 µL) and PEG–400 (150 µL) were added and the reaction mixture was stirred for four hours at RT. The reaction mixture was quenched with water (5 mL) and extracted with EtOAc (3 × 10 mL). The combined organic extracts were dried over Na_2SO_4, filtered, and concentrated *in vacuo*. NMR yield was quantified by ^{1}H NMR analysis of the crude reaction mixture using trichloroethylene (TCE, 9.0 µL, 0.10 mmol, 1.0 equiv.) as an internal standard.

Click reaction between sodium azide and phenyl acetylene:

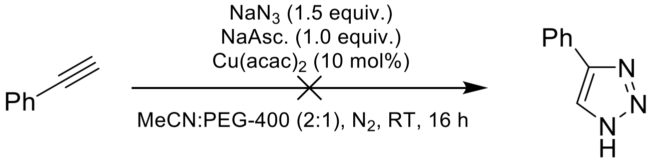

An oven-dried microwave vial equipped with a Teflon-coated stir-bar was charged with sodium azide (13.0 mg, 0.20 mmol, 1.5 equiv.), sodium ascorbate (40.4 mg, 0.20 mmol, 1.0 equiv.), and $Cu(acac)_2$ (5.2 mg, 10 mol%). The vial was capped and placed under an atmosphere of N_2 by evacuating and refilling with N_2 (three cycles). MeCN (1 mL) and PEG–400 (0.5 mL) were added and the reaction mixture was stirred at RT for 5 minutes prior to the addition of phenyl acetylene (21 µL, 0.22 mmol, 1.1 equiv.). The reaction was stirred at RT for 16 hours. The reaction mixture was quenched with water (5 mL) and extracted with EtOAc (3 × 10 mL). The combined organic extracts were dried over Na_2SO_4, filtered, and concentrated *in vacuo*. NMR yield was quantified by ^{1}H NMR analysis of the crude reaction mixture using trichloroethylene (TCE, 9.0 µL, 0.10 mmol, 1.0 equiv.) as an internal standard. Quantitative phenyl acetylene was detected.

3.3. Optimisation of One-pot procedure

Optimisation reactions were performed according to General Procedure A.

Table S6: Catalyst screen

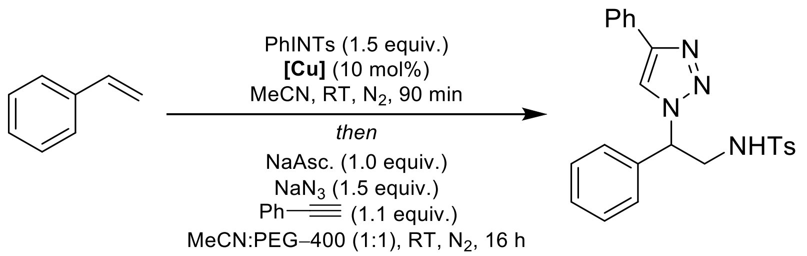

Entry	Catalyst	Yield (%)[a]
1	$Cu(acac)_2$	71
2	$Cu(TFA)_2$	50
3	$CuBr_2$	68

[a] Determined by ^{1}H NMR using TCE as internal standard.

Table S7: Solvent screen

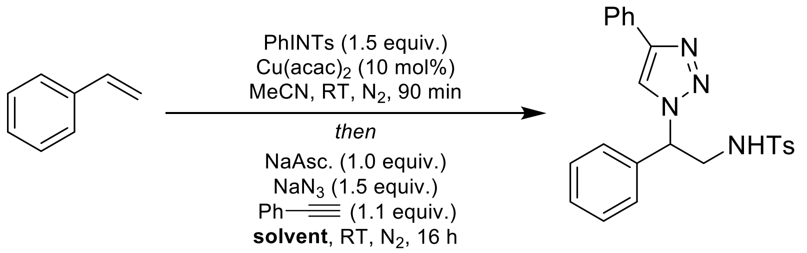

Entry	Solvent	Yield (%)[a]
1	MeCN:PEG–400 (1:1)	66
2	MeCN:1,3-Dioxolane (1:1)	Trace
3	MeCN:Triethylorthoformate (1:1)	Trace
4	MeCN:γ-Butyrolactone (1:1)	Trace
5	MeCN:water (1:1)	20
6	MeCN:Glyme (1:1)	5
7	MeCN:Diglyme (1:1)	7
8	MeCN:1,4-Dioxane (1:1)	0
**9**	**MeCN:PEG–400 (2:1)**	**82**
10	MeCN:PEG–400 (4:1)	42
11	MeCN:PEG–400 (1:2)	65
12	MeCN:PEG–400 (1:4)	72

[a] Determined by ^{1}H NMR using nitromethane as internal standard.

N.B., Isolation of product from PEG–400 was challenging at MeCN:PEG–400 ratios greater than 1:2.

Table S8: Concentration screen

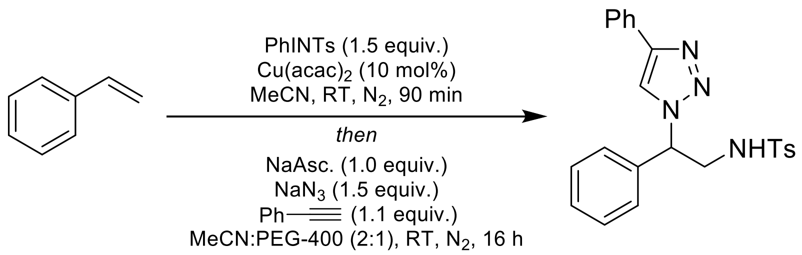

Entry	Reaction concentration	Yield (%)[a]
1	0.100 M	42
2	0.140 M	64
**3**	**0.200 M**	**Quant. (88)**[b]
4	0.400 M	51

[a] Determined by ^{1}H NMR using nitromethane as internal standard; [b] Isolated yield.

3.4. Control experiments

Table S9: Control experiments

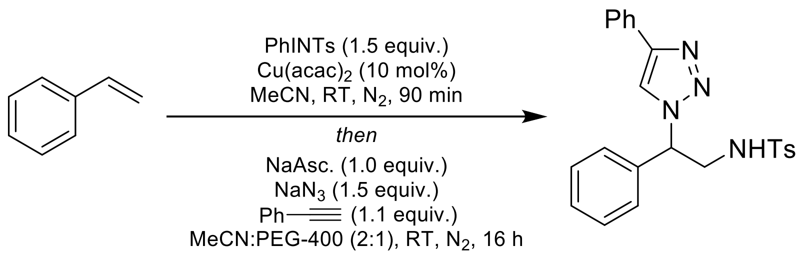

Entry	Change to conditions	Yield (%)[a]
1	No $Cu(acac)_2$	0
2	No NaAsc.	47
3	4 h	48[b]
4	Wet MeCN, under air	12

[a] Determined by ^{1}H NMR using nitromethane as internal standard; [b] Isolated yield.

4. Characterisation data

2-Phenyl-1-tosylaziridine (S1)

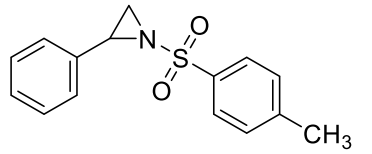

Chemical Formula: $C_{15}H_{15}NO_2S$
Exact Mass: 273.0823
Molecular Weight: 273.3500

An oven-dried flask equipped with a Teflon-coated stir-bar was charged with 4-methyl-*N*-(phenyl-γ^3-iodaneylidene)benzenesulfonamide (221 mg, 0.60 mmol, 2.0 equiv.) The flask was sealed and placed under an atmosphere of Ar by evacuating and refilling with Ar (three cycles). Dry MeCN (3 mL) was added, followed by styrene (34 µL, 0.30 mmol, 1.0 equiv.). Cu(acac)$_2$ (7.8 mg, 10 mol%) was added quickly in one portion. The reaction mixture was stirred under Ar at room temperature for two hours. The solution was partitioned between 1:1 10% aqueous ammonia solution:brine (3 mL) and EtOAc (4 mL). The organic layer was separated, and the aqueous fraction was washed with EtOAc (2 × 3 mL). The combined organic extracts were dried over Na_2SO_4, filtered, and concentrated *in vacuo*. The crude product was purified by flash chromatography (silica gel, 0–20% Et_2O in hexane) to afford the title compound as an amorphous white solid (61 mg, 75%).

^{1}H NMR (500 MHz, CDCl$_3$): δ 7.90 – 7.85 (m, 2H), 7.36 – 7.30 (m, 2H), 7.31 – 7.25 (m, 3H), 7.25 – 7.18 (m, 2H), 3.78 (dd, *J* = 7.2, 4.5 Hz, 1H), 2.98 (d, *J* = 7.2 Hz, 1H), 2.43 (s, 3H), 2.39 (d, *J* = 4.4 Hz, 1H).

The observed spectra are consistent with the literature.[2]

4-Methyl-*N*-methyl-(phenyl-γ^3-iodaneylidene)benzenesulfonamide (S2)

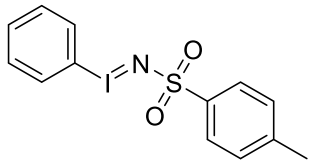

Chemical Formula: $C_{13}H_{12}INO_2S$
Exact Mass: 372.9633
Molecular Weight: 373.2085

Prepared according to General Procedure B, using 4-methylbenzenesulfonamide (6.85 g, 40 mmol, 1.0 equiv.). The product was obtained without further purification after filtration as a pale yellow solid (10.5 g, 70%).

Iminoiodane purity was verified by decomposition point (104.6 °C, *lit*[3] 102–105 °C).

The product was stored in a freezer (–18 °C) under an atmosphere of argon. Prolonged exposure to air at room temperature resulted in degradation.

4-Nitro-*N*-(phenyl-γ^3-iodaneylidene)benzenesulfonamide (S3)

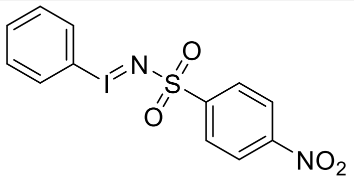

Chemical Formula: $C_{12}H_9IN_2O_4S$
Exact Mass: 403.9328
Molecular Weight: 404.1785

Prepared according to General Procedure B, using 4-nitrobenzenesulfonamide (4.04 g, 20 mmol, 1.0 equiv.). The product was obtained without further purification after filtration as a pale yellow solid (7.78 g, 96%).

Iminoiodane purity was verified by decomposition point (123.6 °C, *lit*[3] 124 °C).

The product was stored in a freezer (–18 °C) under an atmosphere of argon. Prolonged exposure to air at room temperature resulted in degradation.

***N*-(Phenyl-γ^3-iodaneylidene)-4-(trifluoromethyl)benzenesulfonamide (S4)**

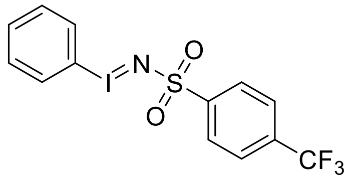

Chemical Formula: $C_{13}H_9F_3INO_2S$
Exact Mass: 426.9351
Molecular Weight: 427.1797

Prepared according to General Procedure B, using 4-(trifluoromethyl)benzenesulfonamide (1.13 g, 5.0 mmol, 1.0 equiv.). The product was obtained without further purification after filtration as a pale yellow solid (1.20 g, 56%).

The product was stored in a freezer (–18 °C) under an atmosphere of argon.

2,6-Difluoro-*N*-(phenyl-γ^3-iodaneylidene)benzenesulfonamide (S5)

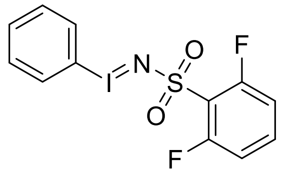

Chemical Formula: $C_{12}H_8F_2INO_2S$
Exact Mass: 394.9289
Molecular Weight: 395.1623

Prepared according to General Procedure B, using 2,6-difluorobenzenesulfonamide (1.45 g, 7.5 mmol, 1.0 equiv.). The product was obtained without further purification after filtration as a pale yellow solid (1.84 g, 62%).

The product was stored in a freezer (–18 °C) under an atmosphere of argon.

2-Vinylbenzofuran (S6)

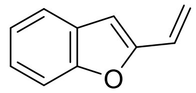

Chemical Formula: $C_{10}H_8O$
Exact Mass: 144.0575
Molecular Weight: 144.1730

Prepared according to General Procedure C, using benzofuran-2-ylboronic acid (356 mg, 2.2 mmol, 1.1 equiv.) and vinyl bromide (2.0 mL, 1.0 M in THF, 2.0 mmol, 1.0 equiv.). The crude product was purified by column chromatography (silica gel, 0–1% EtOAc in hexane) to afford the title compound as a colourless oil (199 mg, 69%).

^{1}H NMR (500 MHz, $CDCl_3$): δ 7.52 (ddd, *J* = 7.7, 1.4, 0.7 Hz, 1H), 7.45 (dq, *J* = 8.1, 0.9 Hz, 1H), 7.27 (ddd, *J* = 8.3, 7.2, 1.3 Hz, 1H), 7.20 (td, *J* = 7.5, 1.0 Hz, 1H), 6.64 (dd, *J* = 17.5, 11.2 Hz, 1H), 6.60 (s, 1H), 5.96 (dd, *J* = 17.5, 1.2 Hz, 1H), 5.38 (dd, *J* = 11.2, 1.2 Hz, 1H).

The observed spectra are consistent with the literature.[4]

6-Vinylquinoline (S7)

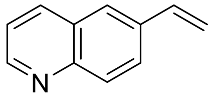

Chemical Formula: $C_{11}H_9N$
Exact Mass: 155.0735
Molecular Weight: 155.2000

Prepared according to General Procedure C, using quinoline-6-ylboronic acid (381 mg, 2.2 mmol, 1.1 equiv.) and vinyl bromide (2.0 mL, 1.0 M in THF, 2.0 mmol, 1.0 equiv.). The crude product was purified by column chromatography (silica gel, 5–10% EtOAc in hexane) to afford the title compound as a colourless oil (148 mg, 48%).

^{1}H NMR (400 MHz, $CDCl_3$): δ 8.86 (d, *J* = 4.0 Hz, 1H), 8.13 (d, *J* = 8.3 Hz, 1H), 8.07 (dd, *J* = 8.8, 2.2 Hz, 1H), 7.91 – 7.84 (m, 1H), 7.72 (s, 1H), 7.39 (dt, *J* = 7.3, 3.1 Hz, 1H), 6.89 (ddd, *J* = 17.8, 11.1, 2.3 Hz, 1H), 5.91 (dd, *J* = 17.6, 2.3 Hz, 1H), 5.40 (dd, *J* = 11.0, 2.3 Hz, 1H).

The observed spectra are consistent with the literature.[5]

2-Vinylbenzo[*b*,*d*]thiophene (S8)

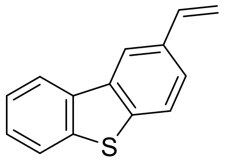

Chemical Formula: $C_{14}H_{10}S$
Exact Mass: 210.0503
Molecular Weight: 210.2940

Prepared according to General Procedure C, using dibenzo[*b*,*d*]thiophen-2-ylboronic acid (1.25 g, 5.5 mmol, 1.1 equiv.) and vinyl bromide (5.0 mL, 1.0 M in THF, 5.0 mmol, 1.0 equiv.). The crude product was purified by column chromatography (silica gel, 0–1% EtOAc in hexane) to afford the title compound as a colourless oil (572 mg, 54%).

^{1}H NMR (500 MHz, $CDCl_3$): δ 8.21 – 8.13 (m, 2H), 7.89 – 7.82 (m, 1H), 7.80 (d, *J* = 8.3 Hz, 1H), 7.56 (dd, *J* = 8.3, 1.7 Hz, 1H), 7.50 – 7.43 (m, 2H), 6.90 (dd, *J* = 17.5, 10.9 Hz, 1H), 5.88 (d, *J* = 17.4 Hz, 1H), 5.33 (d, *J* = 11.1 Hz, 1H).

The observed spectra are consistent with the literature.[6]

1,2,3-Trimethoxy-5-vinylbenzene (S9)

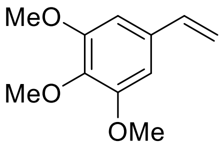

Chemical Formula: $C_{11}H_{14}O_3$
Exact Mass: 194.0943
Molecular Weight: 194.2300

Prepared according to General Procedure C, using (3,4,5-trimethoxyphenyl)boronic acid (466 mg, 2.2 mmol, 1.1 equiv.) and vinyl bromide (2.0 mL, 1.0 M in THF, 2.0 mmol, 1.0 equiv.). The crude product was purified by column chromatography (silica gel, 20–30% Et_2O in hexane) to afford the title compound as a yellow oil (281 mg, 72%).

^{1}H NMR (500 MHz, $CDCl_3$): δ 6.69 – 6.56 (m, 3H), 5.66 (dd, *J* = 17.5, 0.8 Hz, 1H), 5.22 (dd, *J* = 10.8, 0.8 Hz, 1H), 3.88 (s, 6H), 3.85 (s, 3H).

The observed spectra are consistent with the literature.[7]

Methyl 2,6-dichloro-4-vinylbenzoate (S13)

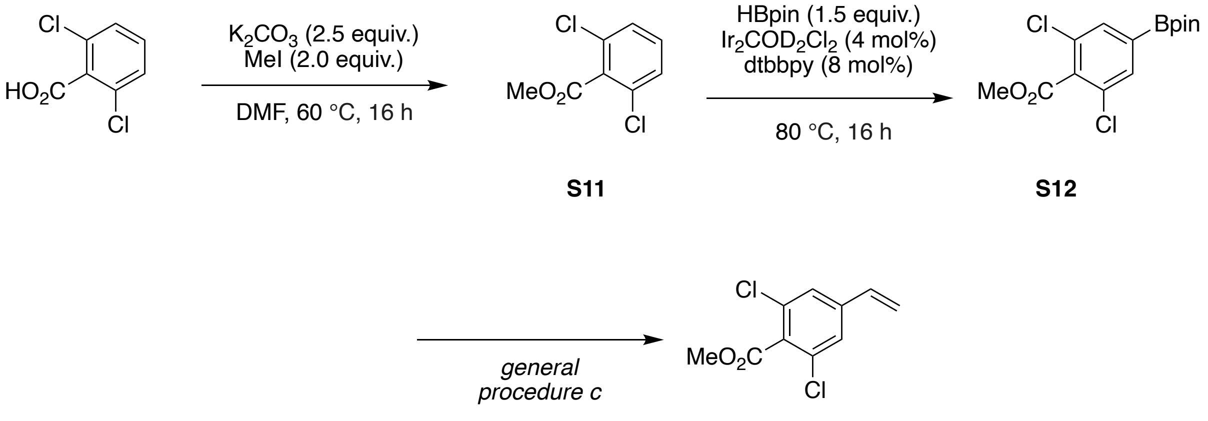

S13

Methyl 2,6-dichlorobenzoate (S11)

A flask was charged with 2,6-dichlorobenzoic acid (2.87 g, 15.0 mmol, 1.0 equiv.), potassium carbonate (5.18 g, 37.5 mmol, 2.5 equiv.), and DMF (37.5 mL, 0.4 M). Iodomethane (1.9 mL, 30.0 mmol, 2.0 equiv.) was added and the flask was heated to 60 °C for 16 hours. The mixture was allowed to cool to room temperature, diluted with water (50 mL), and extracted with EtOAc (3 × 100 mL). The combined organic extracts were washed with brine (250 mL), 10% aq. LiCl (3 × 100 mL), dried over Na_2SO_4, filtered, and concentrated under reduced pressure. The crude material was purified by column chromatography (silica gel, 10% EtOAc in hexane) to afford the title compound as a yellow oil (3.01 g, 98%).

^{1}H NMR (500 MHz, $CDCl_3$): δ 7.35 – 7.25 (m, 4H), 3.98 (s, 3H).

The observed spectra are consistent with the literature.[8]

Methyl 2,6-dichloro-4-(4,4,5,5-tetramethyl-1,3,2-dioxaborolan-2-yl)benzoate (S12)

A flame-dried Schlenk flask was charged with cyclooctadiene iridium chloride dimer (134 mg, 4 mol%) and 4,4′-di-*tert*-butyl-2,2′-dipyridyl (107 mg, 8 mol%), purged and placed under an atmosphere of Ar (three cycles). Pinacolborane (1.09 mL, 7.5 mmol, 1.5 equiv.) was added, followed by methyl 2,6-dichlorobenzoate (**S11**) (1.03 g, 5.00 mmol, 1.0 equiv.) and the mixture was heated to 80 °C for 16 hours. The reaction was allowed to cool to room temperature, exposed to air, diluted with CH_2Cl_2 (50 mL), and concentrated *in vacuo*. The crude product was purified by column chromatography (boron-capped silica gel, 25% CH_2Cl_2 in hexane) to afford the title compound as a clear oil, which solidified in the freezer (1.37 g, 83%).

^{1}H NMR (500 MHz, $CDCl_3$): δ 7.71 (s, 2H), 3.97 (s, 3H), 1.34 (s, 12H).

The observed spectra are consistent with the literature.[9]

Methyl 2,6-dichloro-4-vinylbenzoate (S13)

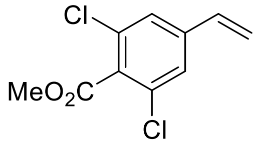

Chemical Formula: $C_{10}H_8Cl_2O_2$
Exact Mass: 229.9901
Molecular Weight: 231.0720

Prepared according to General Procedure C, using methyl 2,6-dichloro-4-(4,4,5,5-tetramethyl-1,3,2-dioxaborolan-2-yl)benzoate (**S12**) (1.09 g, 3.30 mmol, 1.1 equiv.) and vinyl bromide (3.0 mL, 1.0 M in THF, 3.0 mmol, 1.0 equiv.). The crude product was purified by column chromatography (silica gel, 0–30% CH_2Cl_2 in hexane) to afford the title compound as a colourless oil, which could not be separated from methyl 2,6-dichlorobenzoate (**S11**) (670 mg, 46% purity, 44%).

^{1}H NMR (500 MHz, CDCl$_3$): δ 7.32 (s, 2H), 6.58 (dd, *J* = 17.5, 10.9 Hz, 1H), 5.80 (d, *J* = 17.5 Hz, 1H), 5.42 (d, *J* = 10.9 Hz, 1H), 3.96 (s, 3H).

***tert*-Butyl 3-vinyl-1H-indole-1-carboxylate (S14)**

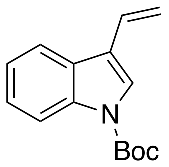

Chemical Formula: $C_{15}H_{17}NO_2$
Exact Mass: 243.1259
Molecular Weight: 243.3060

An oven-dried flask equipped with a Teflon-coated stir-bar was charged with methyltriphenylphosphonium bromide (1.97 g, 5.50 mmol, 1.1 equiv.). The flask was sealed and placed under an atmosphere of N_2 by evacuating and refilling with N_2 (three cycles). Dry THF (15.0 mL, 0.33 M) was added, the mixture was cooled to 0 °C, and a solution of *n*-butyllithium in hexane (2.50 mL, 2.4 M, 6.00 mmol, 1.2 equiv.) was added dropwise. After stirring for 30 minutes at 0 °C, *tert*-butyl 3-formyl-1H-indole-1-carboxylate (1.23 g, 5.00 mmol, 1.0 equiv.) was slowly added. The reaction was allowed to warm to room temperature and stirred for 16 hours. The reaction mixture was then cooled to 0 °C and quenched with aqueous ammonium chloride (15 mL). The biphasic solution was extracted with EtOAc (3 × 20 mL). The combined organic extracts were washed with brine, dried over $MgSO_4$, and concentrated *in vacuo*. The crude product was purified by column chromatography (silica gel, 5% EtOAc in hexane) to afford the title compound as a yellow oil (456 mg, 38%).

^{1}H NMR (400 MHz, CDCl$_3$): δ 8.20 (d, *J* = 8.2 Hz, 1H), 7.81 (d, *J* = 7.8 Hz, 1H), 7.65 (s, 1H), 7.40 – 7.27 (m, 2H), 6.83 (dd, *J* = 17.8, 11.3 Hz, 1H), 5.83 (d, *J* = 17.8 Hz, 1H), 5.34 (d, *J* = 11.3 Hz, 1H), 1.69 (s, 9H).

The observed spectra are consistent with the literature.[10]

6-Methyl-2-(4-vinylphenyl)-1,3,6,2-dioxazaborocane-4,8-dione (S15)

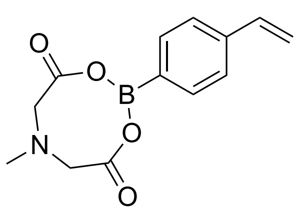

Chemical Formula: $C_{13}H_{14}BNO_4$
Exact Mass: 259.1016
Molecular Weight: 259.0680

An oven-dried flask equipped with a Teflon-coated stir-bar was charged with (4-vinylphenyl)boronic acid (740 mg, 5.00 mmol, 1.0 equiv.) and 2,2'-(methylazanediyl)diacetic acid (736 mg, 5.00 mmol, 1.0 equiv.). The flask was equipped with a reflux condenser and sealed with a rubber septum. The flask was placed under an atmosphere of N_2 by evacuating and refilling with N_2 (three cycles). Dry toluene (90.0 mL) and dry DMSO (10.0 mL, 0.05 M) were added and the resulting mixture was stirred at 100 °C under N_2 for 16 hours. The reaction mixture was allowed to cool to room temperature, then concentrated *in vacuo* to remove toluene. The remaining mixture was diluted with water (50 mL), with instant formation of a precipitate. The aqueous suspension was extracted with a mixture of EtOAc/ acetone (3:1, 3 × 50 mL). The combined organic extracts were dried over Na_2SO_4, filtered, and concentrated *in vacuo*. The crude product was purified by column chromatography (silica gel, 20% hexane in EtOAc) to afford the title compound as a white solid (1.12 g, 87%).

^{1}H NMR (400 MHz, CD_3CN): δ 7.49 – 7.44 (m, 4H), 6.78 (dd, *J* = 17.7, 10.9 Hz, 1H), 5.85 (dd, *J* = 17.7, 1.0 Hz, 1H), 5.27 (dd, *J* = 10.9, 1.0 Hz, 1H), 4.06 (d, *J* = 17.1 Hz, 2H), 3.89 (d, *J* = 17.0 Hz, 2H), 2.50 (s, 3H).

The observed spectra are consistent with the literature.[11]

2-Ethynyl-2,3-dihydro-1*H*-naphtho[1,8-*de*][1,3,2]diazaborinine (S16)

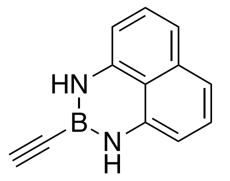

Chemical Formula: $C_{12}H_9BN_2$
Exact Mass: 192.0859
Molecular Weight: 192.0280

An oven-dried flask equipped with a Teflon-coated stir-bar was sealed and placed under an atmosphere of N_2 by evacuating and refilling with N_2 (three cycles). Dry THF (20 mL, 0.5 M) was added and the solvent was cooled to –78 °C. A solution of ethynylmagnesium bromide in THF (27.8 mL, 0.36 M, 10.0 mmol, 1.0 equiv.) was added and the mixture was stirred at –78 ºC for 15 minutes. Trimethyl borate (1.34 mL, 12.0 mmol, 1.2 equiv.) was added dropwise at –78 ºC and the mixture was allowed to stir for two hours whilst allowing to warm to RT. A separate oven-dried flask was equipped with a Teflon-coated stir-bar and charged with naphthalene-1,8-diamine (1.90 g, 12.0 mmol, 1.2 equiv.). The flask was placed under an atmosphere of N_2 by evacuating and refilling with N_2 (three cycles). Dry THF (5 mL, 2.4 M) was added and the naphthalene-1,8-diamine mixture was stirred at RT for five minutes. The naphthalene-1,8-diamine mixture was then added to the alkyne borate solution dropwise, followed by the dropwise addition of acetic acid (740 µL, 13.0 mmol, 1.3 equiv.) to the combined reaction mixture. This was then stirred for two hours at RT, before the mixture was quenched with sat aq. $NaHCO_3$ (20 mL). The phases were separated, and the aqueous phase was extracted with EtOAc (2 × 50 mL). The combined organic extracts were dried over Na_2SO_4, filtered, and concentrated *in vacuo*. The crude product was purified by column chromatography (silica gel, 0–20% EtOAc in hexane) to afford the title compound as a white solid (1.84 g, 96%).

^{1}H NMR (400 MHz, CDCl$_3$): δ 7.18 – 7.05 (m, 4H), 6.32 (dd, *J* = 7.20, 1.17 Hz, 2H), 5.88 (s, 2H), 2.64 (s, 1H).

The observed spectra are consistent with the literature.[12]

Ethynyldiphenylphosphane (S17)

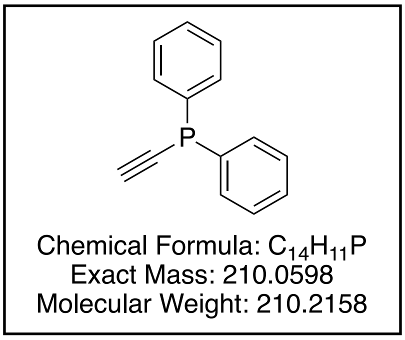

An oven-dried flask equipped with a Teflon-coated stir-bar was sealed and placed under an atmosphere of N_2 by evacuating and refilling with N_2 (three cycles). The flask was charged with ethynyltrimethylsilane (1.52 mL, 11.0 mmol, 1.1 equiv.) and dry THF (10 mL, 1.1 M) under N_2 at RT. The mixture was cooled to 0 °C, a solution of *n*-BuLi in hexane (4.40 mL, 2.5 M, 11.0 mmol, 1.1 equiv.) was added dropwise, and the resulting solution was stirred at this temperature for 30 minutes. A separate oven-dried flask equipped with a Teflon-coated stir-bar was sealed and placed under an atmosphere of N_2 by evacuating and refilling with N_2 (three cycles). This flask was charged with chlorodiphenylphosphane (1.85 mL, 10.0 mmol, 1.0 equiv.) and dry THF (5 mL, 2.0 M) under N_2 and stirred for five minutes at RT. This mixture of chlorodiphenylphosphine was then added dropwise to the ethynyltrimethylsilane mixture at 0 °C, and the reaction mixture was subsequently stirred for one hour whilst allowing to warm to RT. After quenching the reaction mixture with water (20 mL), the biphasic mixture was separated, and the aqueous phase was extracted with a 50:50 mixture of hexane/EtOAc (3 × 50 mL). The combined organic extracts were dried over Na_2SO_4, filtered, and concentrated *in vacuo* to afford the TMS protected product. This was subsequently dissolved in MeOH (10 mL, 1.0 M) and potassium carbonate (2.07 g, 15.0 mmol, 1.5 equiv.) was added. The resulting mixture was stirred for one hour before adding water (20 mL) and separating the biphasic mixture. The aqueous phase was extracted with a 50:50 hexane/EtOAc mixture (3 × 50 mL). The combined organic extracts were dried over Na_2SO_4, filtered, and concentrated *in vacuo*. The crude product was purified by column chromatography (basified silica gel, hexane), to afford the title compound as a white solid (1.18 g, 56%).

^{1}H NMR (500 MHz, CDCl$_3$): δ 7.66 – 7.57 (m, 4H), 7.40 – 7.31 (m, 6H), 3.24 (s, 1H).

The observed spectra are consistent with the literature.[13]

4-Methyl-*N*-(2-phenyl-2-(4-phenyl-1*H*-1,2,3-triazol-1-yl)ethyl)benzenesulfonamide (1)

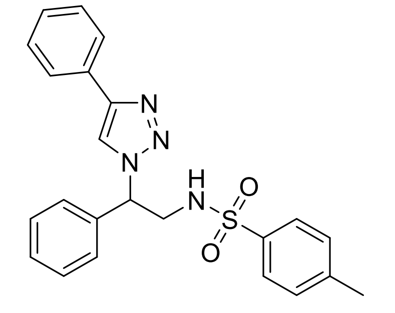

Chemical Formula: $C_{23}H_{22}N_4O_2S$
Exact Mass: 418.1463
Molecular Weight: 418.5150

Prepared according to General Procedure A, using PhINTs (170 mg, 0.45 mmol, 1.5 equiv.), styrene (35.0 µL, 0.30 mmol, 1.0 equiv.), and phenylacetylene (37.0 µL, 0.33 mmol, 1.1 equiv.). The crude product was purified by column chromatography (silica gel, 20% acetone in hexane) to afford the title compound as a white amorphous solid (113 mg, 88%).

^{1}H NMR (500 MHz, DMSO-*d*$_6$): δ 8.76 (s, 1H), 8.07 (t, *J* = 6.0 Hz, 1H, N*H*), 7.82 (dd, *J* = 8.0, 1.4 Hz, 2H), 7.69 – 7.64 (m, 2H), 7.48 – 7.29 (m, 10H), 5.84 (dd, *J* = 9.5, 5.5 Hz, 1H), 3.85 (ddd, *J* = 13.9, 9.5, 6.3 Hz, 1H), 3.54 – 3.45 (m, 1H), 2.33 (s, 3H).

^{13}C{^{1}H} NMR (126 MHz, DMSO-*d*$_6$): δ 146.4, 142.9, 137.3, 137.0, 130.6, 129.7, 128.9, 128.9, 128.7, 127.9, 127.1, 126.5, 125.1, 120.9, 64.3, 46.0, 20.9.

IR (neat): 3253, 2357, 1456, 1329, 1159, 1094, 813 cm^{-1}.

HRMS (ESI): [M+Na$^+$] ($C_{23}H_{22}N_4O_2SNa^+$) requires *m*/*z* 441.1356, found *m*/*z* 441.1362.

***N*-(2-(2-Bromophenyl)-2-(4-phenyl-1*H*-1,2,3-triazol-1-yl)ethyl)-4-methylbenzenesulfonamide (2)**

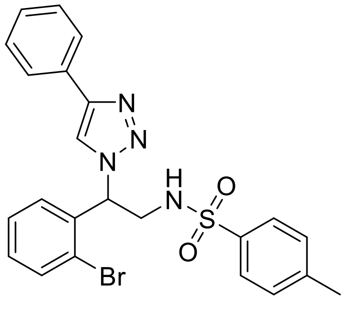

Chemical Formula: $C_{23}H_{21}BrN_4O_2S$
Exact Mass: 496.0569
Molecular Weight: 497.4110

Prepared according to General Procedure A, using PhINTs (170 mg, 0.45 mmol, 1.5 equiv.), 2-bromostyrene (38.0 µL, 0.30 mmol, 1.0 equiv.), and phenylacetylene (37.0 µL, 0.33 mmol, 1.1 equiv.). The crude product was purified by column chromatography (silica gel, 20% acetone in hexane) to afford the title compound as a yellow amorphous solid (83 mg, 55%).

^{1}H NMR (500 MHz, Acetone-*d*$_6$): δ 8.42 (s, 1H), 7.92 – 7.86 (m, 2H), 7.80 – 7.74 (m, 2H), 7.70 – 7.65 (m, 1H), 7.47 – 7.26 (m, 8H), 7.05 (br. s, 1H, N*H*), 6.34 (dd, *J* = 9.8, 4.8 Hz, 1H), 4.03 (ddd, *J* = 14.7, 9.7, 5.3 Hz, 1H), 3.80 – 3.73 (m, 1H), 2.39 (s, 3H).

^{13}C{^{1}H} NMR (126 MHz, Acetone-*d*$_6$): δ 147.9, 144.2, 138.9, 137.0, 134.2, 132.0, 131.5, 130.6, 129.6, 129.4, 129.3, 128.8, 127.8, 126.3, 123.9, 122.2, 64.4, 46.6, 21.4.

IR (thin film): 3348, 2361, 1333, 1159, 1092, 764 cm^{-1}.

HRMS (ESI): [M+H$^+$] ($C_{23}H_{22}BrN_4O_2S^+$) requires *m*/*z* 497.0641, found *m*/*z* 497.0632.

N-(2-(3-Fluorophenyl)-2-(4-phenyl-1*H*-1,2,3-triazol-1-yl)ethyl)-4-methylbenzenesulfonamide (3)

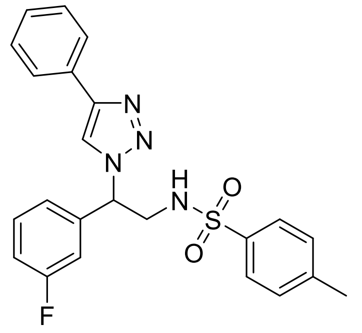

Chemical Formula: $C_{23}H_{21}FN_4O_2S$
Exact Mass: 436.1369
Molecular Weight: 436.5054

Prepared according to General Procedure A, using PhINTs (168 mg, 0.45 mmol, 1.5 equiv.), 3-fluorostyrene (36.0 µL, 0.30 mmol, 1.0 equiv.), and phenylacetylene (37.0 µL, 0.33 mmol, 1.1 equiv.). The crude product was purified by column chromatography (silica gel, 20–50% EtOAc in hexane) to afford the title compound as a yellow amorphous solid (61 mg, 46%).

^{1}H NMR (500 MHz, Acetone-*d*$_6$): δ 8.46 (s, 1H), 7.90 – 7.84 (m, 2H), 7.78 – 7.72 (m, 2H), 7.48 – 7.40 (m, 3H), 7.39 – 7.35 (m, 2H), 7.32 (ddt, *J* = 8.0, 6.8, 1.3 Hz, 1H), 7.29 – 7.24 (m, 1H), 7.24 – 7.19 (m, 1H), 7.13 (tdd, *J* = 8.6, 2.7, 0.9 Hz, 1H), 6.97 (t, *J* = 6.4 Hz, 1H, N*H*), 5.99 (dd, *J* = 9.2, 5.6 Hz, 1H), 4.06 (ddd, *J* = 14.1, 9.1, 6.3 Hz, 1H), 3.79 (ddd, *J* = 14.2, 6.5, 5.6 Hz, 1H), 2.39 (s, 3H).

^{13}C{^{1}H} NMR (126 MHz, Acetone-*d*$_6$): δ 164.0 (d, $^{1}J_{CF}$ = 245.2 Hz), 148.3, 144.4, 141.1 (d, $^{3}J_{CF}$ = 7.4 Hz), 139.1, 132.3, 132.1 (d, $^{3}J_{CF}$ = 8.1 Hz), 130.8, 129.9, 129.0, 128.0, 126.5, 124.5, 121.9, 116.7 (d, $^{2}J_{CF}$ = 21.2 Hz), 115.3 (d, $^{2}J_{CF}$ = 22.9 Hz), 65.4, 47.8, 21.6.

^{19}F NMR (377 MHz, Acetone-*d*$_6$): δ –113.5.

IR (thin film): 3343, 2363, 1456, 1339, 1157, 1092, 766 cm^{-1}.

HRMS (ESI): [M+Na^{+}] ($C_{23}H_{21}FN_4O_2SNa^{+}$) requires *m/z* 459.1262, found *m/z* 459.1252.

Methyl 4-(2-((4-methylphenyl)sulfonamido)-1-(4-phenyl-1*H*-1,2,3-triazol-1-yl)ethyl)benzoate (4)

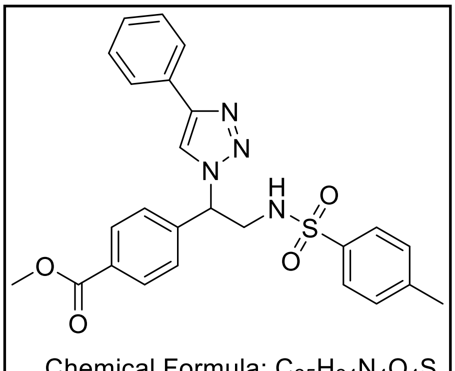

Chemical Formula: $C_{25}H_{24}N_4O_4S$
Exact Mass: 476.1518
Molecular Weight: 476.5510

Prepared according to General Procedure A, using PhINTs (168 mg, 0.45 mmol, 1.5 equiv.), methyl 4-vinylbenzoate (47.8 mg, 0.30 mmol, 1.0 equiv.), and phenylacetylene (37.0 µL, 0.33 mmol, 1.1 equiv.). The crude product was purified by column chromatography (silica gel, 20–50% EtOAc in hexane) to afford the title compound as a white amorphous solid (99 mg, 69%).

^{1}H NMR (500 MHz, Acetone-*d*$_6$): δ 8.47 (s, 1H), 8.02 – 7.97 (m, 2H), 7.88 – 7.86 (m, 2H), 7.75 – 7.71 (m, 2H), 7.55 – 7.52 (m, 2H), 7.45 – 7.31 (m, 5H), 6.98 (t, *J* = 6.3 Hz, 1H, N*H*), 6.05 (dd, *J* = 9.1, 5.5 Hz, 1H), 4.09 (ddd, *J* = 14.2, 9.1, 6.3 Hz, 1H), 3.87 (s, 3H), 3.82 (ddd, *J* = 14.2, 6.5, 5.5 Hz, 1H), 2.38 (s, 3H.

^{13}C{^{1}H} NMR (126 MHz, Acetone-*d*$_6$): δ 166.8, 148.1, 144.3, 143.2, 139.0, 132.1, 131.6, 130.8, 130.7, 129.8, 128.9, 128.5, 127.9, 126.4, 121.9, 65.5, 52.6, 47.7, 21.5.

IR (thin film): 3325, 2356, 1717, 1558, 1506, 1287, 1159, 766 cm^{-1}.

HRMS (ESI): [M+H^{+}] ($C_{25}H_{25}N_4O_4S^{+}$) requires *m/z* 477.1591, found *m/z* 477.1584.

***N*-(2-(4-Methoxyphenyl)-2-(4-phenyl-1*H*-1,2,3-triazol-1-yl)ethyl)-4-methylbenzenesulfonamide (5)**

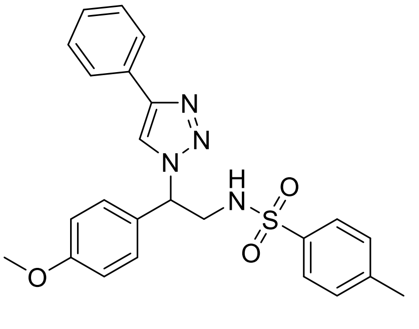

Chemical Formula: $C_{24}H_{24}N_4O_3S$
Exact Mass: 448.1569
Molecular Weight: 448.5410

Prepared according to General Procedure A, using PhINTs (168 mg, 0.45 mmol, 1.5 equiv.), 1-methoxy 4-vinylbenzene (40.0 µL, 0.30 mmol, 1.0 equiv.), and phenylacetylene (37.0 µL, 0.33 mmol, 1.1 equiv.). The crude product was purified by column chromatography (silica gel, 10–30% EtOAc in hexane) to afford the title compound as a white amorphous solid (112 mg, 83%).

^{1}H NMR (500 MHz, DMSO-*d*$_6$): δ 8.71 (s, 1H), 8.02 (t, *J* = 5.9 Hz, 1H, N*H*), 7.83 – 7.78 (m, 2H), 7.67 – 7.64 (m, 2H), 7.44 (t, *J* = 7.7 Hz, 2H), 7.36 – 7.31 (m, 5H), 6.94 – 6.90 (m, 2H), 5.77 (dd, *J* = 9.3, 5.7 Hz, 1H), 3.81 (ddd, *J* = 13.8, 9.3, 6.2 Hz, 1H), 3.73 (s, 3H), 3.46 (dt, *J* = 13.8, 5.7 Hz, 1H), 2.34 (s, 3H).

^{13}C{^{1}H} NMR (126 MHz, DMSO-*d*$_6$): δ 159.4, 146.4, 142.8, 137.3, 130.7, 129.7, 129.0, 128.9, 128.5, 127.9, 126.4, 125.1, 120.6, 114.2, 63.7, 55.2, 46.1, 20.9.

IR (thin film): 3347, 2359, 1516, 1254, 1159, 1094, 1032, 814, 766 cm^{-1}.

HRMS (ESI): [M+H$^+$] ($C_{24}H_{25}N_4O_3S^+$) requires *m*/*z* 449.1642, found *m*/*z* 449.1627.

***N*-(2-Benzofuran-2-yl)-2-(4-phenyl-1*H*-1,2,3-triazol-1-yl)ethyl)-4-methylbenzenesulfonamide (6)**

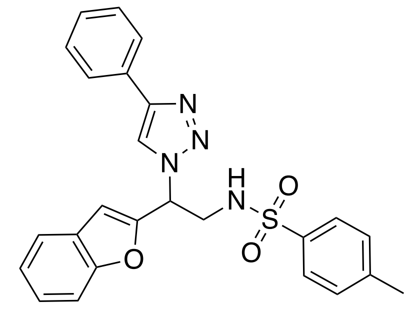

Chemical Formula: $C_{25}H_{22}N_4O_3S$
Exact Mass: 458.1413
Molecular Weight: 458.5360

Prepared according to General Procedure A, using PhINTs (168 mg, 0.45 mmol, 1.5 equiv.), 2-vinylbenzofuran (43.4 mg, 0.30 mmol, 1.0 equiv.), and phenylacetylene (37.0 µL, 0.33 mmol, 1.1 equiv.). The crude product was purified by column chromatography (silica gel, 10–20% acetone in hexane) to afford the title compound as a white amorphous solid (60 mg, 43%).

^{1}H NMR (500 MHz, Acetone-*d*$_6$): δ 8.49 (s, 1H), 7.90 – 7.86 (m, 2H), 7.77 – 7.73 (m, 2H), 7.63 (dt, *J* = 7.7, 1.0 Hz, 1H), 7.48 (dt, *J* = 8.3, 0.9 Hz, 1H), 7.45 – 7.40 (m, 2H), 7.36 – 7.30 (m, 4H), 7.25 (td, *J* = 7.5, 1.0 Hz, 1H), 7.10 (t, *J* = 6.5 Hz, 1H, N*H*), 7.03 (d, *J* = 0.9 Hz, 1H), 6.25 – 6.20 (m, 1H), 4.08 – 4.00 (m, 2H), 2.37 (s, 3H).

^{13}C{^{1}H} NMR (126 MHz, Acetone-*d*$_6$): δ 155.9, 152.9, 148.2, 144.3, 138.9, 132.1, 130.7, 130.3, 129.7, 128.9, 127.8, 126.4, 126.1, 124.2, 122.6, 121.3, 112.2, 107.2, 59.7, 46.0, 21.5.

IR (thin film): 3325, 2359, 2322, 1558, 1456, 1339, 1159, 1094, 813, 752 cm^{-1}.

HRMS (ESI): [M+Na$^+$] ($C_{25}H_{22}N_4O_3SNa^+$) requires *m*/*z* 481.1305, found *m*/*z* 481.1305.

4-Methyl-*N*-(2-(4-phenyl-1*H*-1,2,3-triazol-1-yl)-2-(quinolin-6-yl)ethyl)benzenesulfonamide (7)

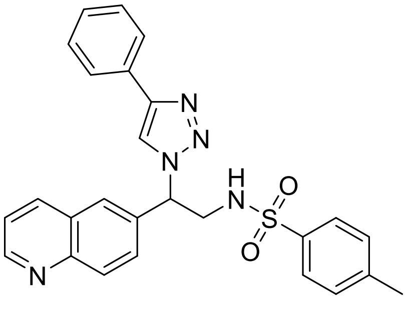

Chemical Formula: $C_{26}H_{23}N_5O_2S$
Exact Mass: 469.1572
Molecular Weight: 469.5630

Prepared according to General Procedure A, using PhINTs (168 mg, 0.45 mmol, 1.5 equiv.), 6-vinylquinoline (46.6 mg, 0.30 mmol, 1.0 equiv.), and phenylacetylene (37.0 µL, 0.33 mmol, 1.1 equiv.). The crude product was purified by column chromatography (silica gel, 20–100% acetone in hexane) to afford a crude solid, which was suspended in a minimum volume of acetone and filtered through a Büchner funnel, with the resulting solid washed with ice-cold acetone (*c.a.* 20 mL) to afford the title compound as a pale yellow amorphous solid (23 mg, 17%).

^{1}H NMR (500 MHz, DMSO-*d*$_6$): δ 8.92 (dd, *J* = 4.2, 1.7 Hz, 1H), 8.81 (s, 1H), 8.41 – 8.35 (m, 1H), 8.13 (s, 1H, N*H*), 8.04 – 7.99 (m, 2H), 7.82 (dd, *J* = 8.3, 1.3 Hz, 2H), 7.77 (dd, *J* = 8.8, 2.1 Hz, 1H), 7.68 – 7.63 (m, 2H), 7.56 (dd, *J* = 8.3, 4.2 Hz, 1H), 7.48 – 7.41 (m, 2H), 7.37 – 7.29 (m, 3H), 6.05 (dd, *J* = 8.9, 6.0 Hz, 1H), 3.96 (dd, *J* = 13.9, 8.9 Hz, 1H), 3.69 (dd, *J* = 13.9, 6.0 Hz, 1H), 2.32 (s, 3H).

^{13}C{^{1}H} NMR (126 MHz, DMSO-*d*$_6$): δ 151.3, 147.4, 146.5, 142.8, 137.4, 136.3, 134.9, 130.6, 129.7, 129.7, 128.9, 128.3, 128.0, 127.6, 127.0, 126.4, 125.1, 122.0, 121.1, 64.0, 45.9, 20.9.

IR (thin film): 3334, 2357, 1558, 1506, 1339, 1155, 1094, 766 cm^{-1}.

HRMS (ESI): [M+H$^+$] ($C_{26}H_{24}N_5O_2S^+$) requires *m*/*z* 470.1645, found *m*/*z* 470.1641.

4-Methyl-*N*-(1-phenyl-1-(4-phenyl-1*H*-1,2,3-triazol-1-yl)propan-2-yl)benzenesulfonamide (8)

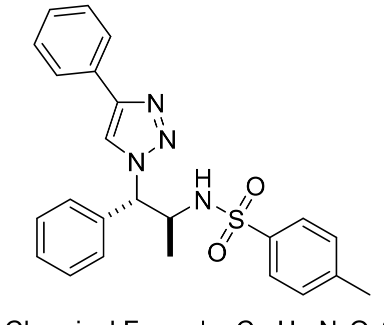

Chemical Formula: $C_{24}H_{24}N_4O_2S$
Exact Mass: 432.1620
Molecular Weight: 432.5420

Prepared according to General Procedure A, using PhINTs (168 mg, 0.45 mmol, 1.5 equiv.), (*E*)-prop-1-en-1-ylbenzene (39.0 µL, 0.30 mmol, 1.0 equiv.), and phenylacetylene (37.0 µL, 0.33 mmol, 1.1 equiv.). The crude product was purified by column chromatography (silica gel, 20–30% acetone in hexane) to afford the title compound as a white amorphous solid (81 mg, 62%, >20:1 *dr*).

^{1}H NMR (500 MHz, Acetone-*d*$_6$): δ 8.56 (s, 1H), 7.90 – 7.85 (m, 2H), 7.61 – 7.55 (m, 2H), 7.49 – 7.45 (m, 2H), 7.41 (dd, *J* = 8.4, 7.0 Hz, 2H), 7.33 – 7.23 (m, 6H), 6.68 (d, *J* = 9.1 Hz, 1H, N*H*), 5.76 (d, *J* = 8.7 Hz, 1H), 4.52 – 4.42 (m, 1H), 2.40 (s, 3H), 1.07 (d, *J* = 6.6 Hz, 3H).

^{13}C{^{1}H} NMR (126 MHz, Acetone-*d*$_6$): δ 147.9, 143.8, 139.8, 137.7, 132.0, 130.4, 129.6, 129.4, 129.2, 129.0, 128.7, 127.6, 126.2, 121.7, 70.2, 53.6, 21.4, 18.9.

IR (thin film): 3326, 1599, 1456, 1329, 1155, 1090, 814, 766 cm^{-1}.

HRMS (ESI): [M+Na$^+$] ($C_{24}H_{24}N_4O_2SNa^+$) requires *m*/*z* 455.1512, found *m*/*z* 455.1517.

Ethyl 2-((4-Methylphenyl)sulfonamido)-3-phenyl-3-(4-phenyl-1*H*-1,2,3-triazol-1-yl) (9)

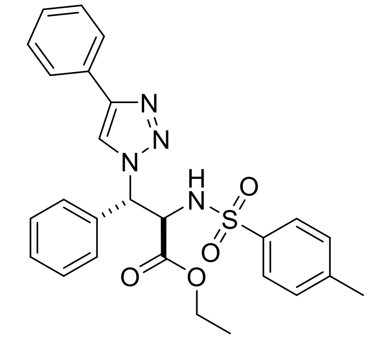

Chemical Formula: $C_{26}H_{26}N_4O_4S$
Exact Mass: 490.1675
Molecular Weight: 490.5780

Prepared according to General Procedure A, using PhINTs (112 mg, 0.30 mmol, 1.0 equiv.), ethyl cinnamate (75.6 µL, 0.30 mmol, 1.0 equiv.), and phenylacetylene (37.0 µL, 0.33 mmol, 1.1 equiv.). The crude product was purified by column chromatography (silica gel, 20% acetone in hexane) to afford the title compound as a white amorphous solid, which coeluted with tosylamine (55 mg, 74% purity, 28%, >20:1 *dr*).

^{1}H NMR (500 MHz, Acetone-d_6): δ 8.56 (s, 1H), 7.88 – 7.81 (m, 2H), 7.64 – 7.56 (m, 4H), 7.44 – 7.33 (m, 6H), 7.35 – 7.27 (m, 3H), 6.07 (d, *J* = 10.3 Hz, 1H, N*H*), 5.10 (t, *J* = 10.2 Hz, 1H), 3.75 – 3.58 (m, 2H), 2.39 (s, 3H), 0.84 (t, *J* = 7.1 Hz, 3H).

^{13}C{^{1}H} NMR (126 MHz, Acetone-d_6): δ 169.8, 148.2, 144.3, 138.8, 136.1, 131.9, 130.4, 129.9, 129.7, 129.5, 128.9, 127.9, 126.3, 121.4, 66.3, 62.2, 59.8, 21.4, 13.9.

IR (thin film): 3310, 1738, 1456, 1337, 1159, 1092, 814, 766 cm^{-1}.

HRMS (ESI): [M+Na$^+$] ($C_{26}H_{26}N_4O_4SNa^+$) requires *m*/*z* 513.1567, found *m*/*z* 513.1562.

4-Methyl-*N*-(2-phenyl-2-(4-phenyl-1*H*-1,2,3-triazol-1-yl)propyl)benzenesulfonamide (10)

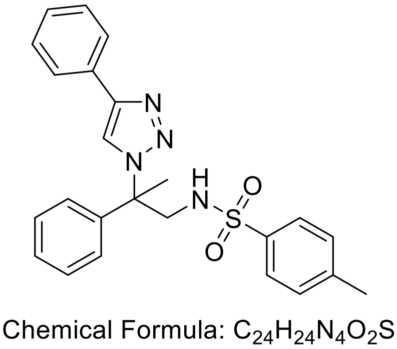

Chemical Formula: $C_{24}H_{24}N_4O_2S$
Exact Mass: 432.1620
Molecular Weight: 432.5420

Prepared according to General Procedure A, using PhINTs (168 mg, 0.45 mmol, 1.5 equiv.), (*E*)-prop-1-en-2-ylbenzene (39.0 µL, 0.30 mmol, 1.0 equiv.), and phenylacetylene (37.0 µL, 0.33 mmol, 1.1 equiv.). The crude product was purified by column chromatography (silica gel, 20% acetone in hexane) to afford the title compound as a white amorphous solid (113 mg, 87%).

^{1}H NMR (500 MHz, Acetone-d_6): δ 8.46 (s, 1H), 7.93 – 7.88 (m, 2H), 7.79 – 7.76 (m, 2H), 7.43 (dd, *J* = 8.4, 7.0 Hz, 2H), 7.38 (d, *J* = 8.0 Hz, 2H), 7.36 – 7.30 (m, 4H), 7.10 – 7.05 (m, 2H), 6.68 (t, *J* = 7.1 Hz, 1H, N*H*), 4.08 (dd, *J* = 13.7, 6.2 Hz, 1H), 3.78 (dd, *J* = 13.7, 8.0 Hz, 1H), 2.40 (s, 3H), 2.18 (s, 3H).

^{13}C{^{1}H} NMR (126 MHz, Acetone-d_6): δ 148.0, 144.0, 143.7, 139.1, 132.1, 130.5, 129.63, 129.60, 128.9, 128.7, 127.8, 126.2, 121.2, 68.1, 52.7, 25.4, 21.4.

IR (thin film): 3319, 1600, 1331, 1159, 1092, 1074, 814, 764 cm^{-1}.

HRMS (ESI): [M+H$^+$] ($C_{24}H_{25}N_4O_2S^+$) requires *m*/*z* 433.1693, found *m*/*z* 433.1693.

N-(2,2-Diphenyl-2-(4-phenyl-1H-1,2,3-triazol-1-yl)ethyl)-4-methylbenzenesulfonamide (11)

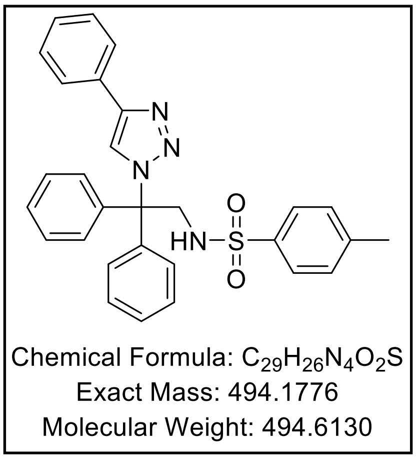

Prepared according to General Procedure A, using PhINTs (168 mg, 0.45 mmol, 1.5 equiv.), ethene-1,1-diylbenzene (53.0 µL, 0.30 mmol, 1.0 equiv.), and phenylacetylene (37.0 µL, 0.33 mmol, 1.1 equiv.). The crude product was purified by column chromatography (silica gel, 20% acetone in hexane) to afford the title compound as a white amorphous solid (119 mg, 80%).

^{1}H NMR (500 MHz, Acetone-d_6): δ 8.07 (s, 1H), 7.89 – 7.85 (m, 2H), 7.76 – 7.73 (m, 2H), 7.43 – 7.37 (m, 10H), 7.33 – 7.29 (m, 1H), 7.17 – 7.13 (m, 4H), 6.54 (t, J = 6.9 Hz, 1H, NH), 4.44 (d, J = 7.0 Hz, 2H), 2.42 (s, 3H).

^{13}C{^{1}H} NMR (126 MHz, Acetone-d_6): δ 147.2, 144.2, 141.1, 138.6, 131.7, 130.6, 130.2, 129.6, 129.4, 128.9, 127.9, 126.9, 126.4, 123.2, 74.2, 52.8, 21.4.

IR (thin film): 3317, 2359, 1558, 1506, 1339, 1162, 1092, 764 cm^{-1}.

HRMS (ESI): [M+Na$^+$] ($C_{29}H_{26}N_4O_2SNa^+$) requires m/z 517.1669, found m/z 517.1667.

N-(2-(4-(2-Chlorophenyl)-1H-1,2,3-triazol-1-yl)-2-phenylethyl)-4-methylbenzenesulfonamide (12)

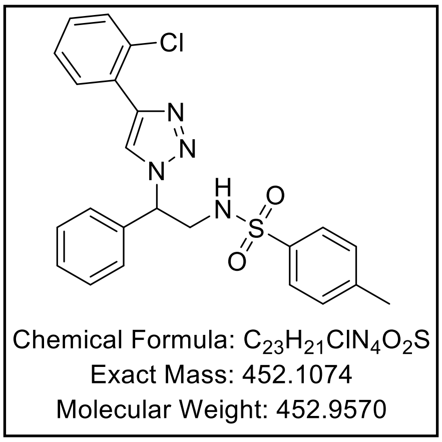

Prepared according to General Procedure A, using PhINTs (171 mg, 0.45 mmol, 1.5 equiv.), styrene (35.0 µL, 0.30 mmol, 1.0 equiv.), and 2-chlorophenyl acetylene (40.8 µL, 0.33 mmol, 1.1 equiv.). The crude product was purified by column chromatography (silica gel, 20–40% EtOAc in hexane) to afford the title compound as a white amorphous solid (65 mg, 47%).

^{1}H NMR (500 MHz, Acetone-d_6): δ 8.64 (s, 1H), 8.23 (dd, J = 7.9, 1.7 Hz, 1H), 7.77 – 7.73 (m, 2H), 7.51 (dd, J = 8.0, 1.3 Hz, 1H), 7.46 – 7.42 (m, 3H), 7.40 – 7.34 (m, 6H), 6.98 (t, J = 6.3 Hz, 1H, NH), 6.02 (dd, J = 9.6, 5.2 Hz, 1H), 4.13 (ddd, J = 14.2, 9.7, 6.4 Hz, 1H), 3.76 (ddd, J = 14.2, 6.3, 5.2 Hz, 1H), 2.89 (s, 3H).

^{13}C{^{1}H} NMR (126 MHz, Acetone-d_6): δ 144.09, 144.05, 138.8, 138.2, 131.6, 131.1, 130.54, 130.48, 130.4, 130.0, 129.8, 129.6, 128.1, 128.0, 127.7, 124.9, 65.8, 47.7, 21.4.

IR (thin film): 3315, 1602, 1331, 1157, 1092, 814, 762 cm^{-1}.

HRMS (ESI): [M+H$^+$] ($C_{23}H_{22}ClN_4O_2S^+$) requires m/z 453.1147, found m/z 453.1141.

N-(2-(4-(3-Chlorophenyl)-1H-1,2,3-triazol-1-yl)-2-phenylethyl)-4-methylbenzenesulfonamide (13)

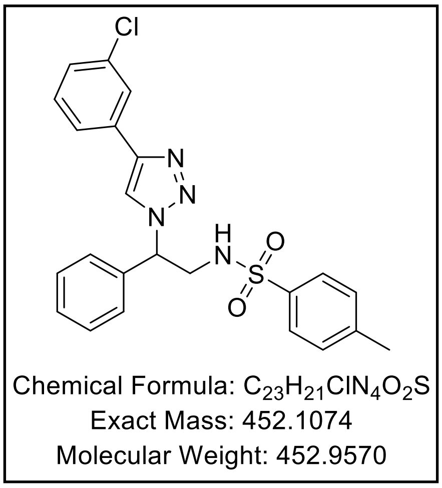

Prepared according to General Procedure A, using PhINTs (171 mg, 0.45 mmol, 1.5 equiv.), styrene (35.0 µL, 0.30 mmol, 1.0 equiv.), and 3-chlorophenyl acetylene (41.4 µL, 0.33 mmol, 1.1 equiv.). The crude product was purified by column chromatography (silica gel, 20–40% EtOAc in hexane) to afford the title compound as a white amorphous solid (91 mg, 66%).

^{1}H NMR (500 MHz, Acetone-d_6): δ 8.53 (s, 1H), 7.89 (t, *J* = 1.9 Hz, 1H), 7.82 (dt, *J* = 7.8, 1.3 Hz, 1H), 7.76 – 7.73 (m, 2H), 7.47 – 7.32 (m, 9H), 6.97 (t, *J* = 6.3 Hz, 1H, N*H*), 5.96 (dd, *J* = 9.6, 5.2 Hz, 1H), 4.13 – 4.01 (m, 1H), 3.80 – 3.71 (m, 1H), 2.38 (s, 3H).

^{13}C{^{1}H} NMR (126 MHz, Acetone-d_6): δ 146.5, 144.1, 138.8, 138.0, 135.1, 134.1, 131.4, 130.6, 129.8, 129.6, 128.5, 128.0, 127.7, 125.9, 124.6, 122.2, 65.9, 47.7, 21.4.

IR (thin film): 3317, 2357, 1607, 1456, 1331, 1157, 1094, 814, 787 cm^{-1}.

HRMS (ESI): [M+H$^+$] ($C_{23}H_{22}ClN_4O_2S^+$) requires *m/z* 453.1147, found *m/z* 453.1152.

N-(2-(4-(4-Chlorophenyl)-1H-1,2,3-triazol-1-yl)-2-phenylethyl)-4-methylbenzenesulfonamide (14)

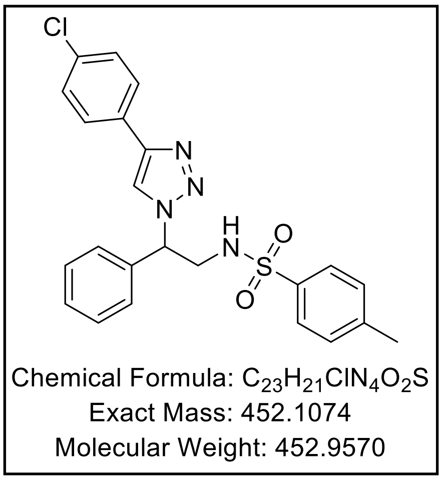

Prepared according to General Procedure A, using PhINTs (171 mg, 0.45 mmol, 1.5 equiv.), styrene (35.0 µL, 0.30 mmol, 1.0 equiv.), and 4-chlorophenyl acetylene (45.9 mg, 0.33 mmol, 1.1 equiv.). The crude product was purified by column chromatography (silica gel, 20–40% EtOAc in hexane) to afford the title compound as a white amorphous solid (99 mg, 71%).

^{1}H NMR (500 MHz, Acetone-d_6): δ 8.47 (s, 1H), 7.91 – 7.85 (m, 2H), 7.76 – 7.73 (m, 2H), 7.48 – 7.44 (m, 2H), 7.43 – 7.33 (m, 7H), 6.94 (t, *J* = 6.3 Hz, 1H, N*H*), 5.94 (dd, *J* = 9.5, 5.2 Hz, 1H), 4.12 – 4.01 (m, 1H), 3.74 (ddd, *J* = 14.2, 6.5, 5.4 Hz, 1H), 2.39 (s, 3H).

^{13}C{^{1}H} NMR (126 MHz, Acetone-d_6): δ 146.8, 144.1, 138.9, 138.1, 133.8, 131.0, 130.6, 129.82, 129.76, 129.6, 128.0, 127.8, 127.7, 121.8, 65.9, 47.7, 21.4.

IR (thin film): 3340, 1441, 1319, 1148, 1089, 912, 822, 812 cm^{-1}.

HRMS (ESI): [M+Na$^+$] ($C_{23}H_{21}ClN_4O_2SNa^+$) requires *m/z* 475.0966, found *m/z* 475.0969.

4-Methyl-*N*-(2-(4-(4-nitrophenyl)-1*H*-1,2,3-triazol-1-yl)-2-phenylethyl)benzenesulfonamide (15)

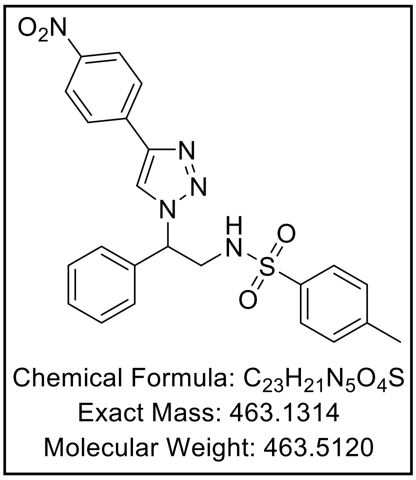

Prepared according to General Procedure A, using PhINTs (171 mg, 0.45 mmol, 1.5 equiv.), styrene (35.0 µL, 0.30 mmol, 1.0 equiv.), and 1-ethynyl-4-nitrobenzene (49.4 mg, 0.33 mmol, 1.1 equiv.). The crude product was purified by column chromatography (silica gel, 20–50% EtOAc in hexane) to afford the title compound as a yellow amorphous solid (104 mg, 74%).

^{1}H NMR (500 MHz, Acetone-*d*$_6$): δ 8.71 (s, 1H), 8.33 – 8.28 (m, 2H), 8.15 – 8.11 (m, 2H), 7.77 – 7.73 (m, 2H), 7.46 – 7.42 (m, 2H), 7.41 – 7.34 (m, 5H), 6.99 (t, *J* = 6.3 Hz, 1H, N*H*), 6.01 (dd, *J* = 9.7, 5.2 Hz, 1H), 4.11 (ddd, *J* = 14.2, 9.7, 6.5 Hz, 1H), 3.77 (ddd, *J* = 14.2, 6.3, 5.2 Hz, 1H), 2.37 (s, 3H).

^{13}C{^{1}H} NMR (126 MHz, Acetone-*d*$_6$): δ 148.1, 146.0, 144.2, 138.8, 138.3, 137.8, 130.6, 129.9, 129.7, 128.1, 127.7, 126.9, 125.0, 123.6, 66.1, 47.7, 21.4.

IR (thin film): 3336, 2342, 1516, 1321, 1159, 1094, 854, 816 cm^{-1}.

HRMS (ESI): [M+Na$^+$] ($C_{23}H_{21}N_5O_4SNa^+$) requires *m*/*z* 486.1207, found *m*/*z* 486.1203.

***N*-(2-(4-(4-methoxyphenyl)-1*H*-1,2,3-triazol-1-yl)-2-phenylethyl)-4-methylbenzenesulfonamide (16)**

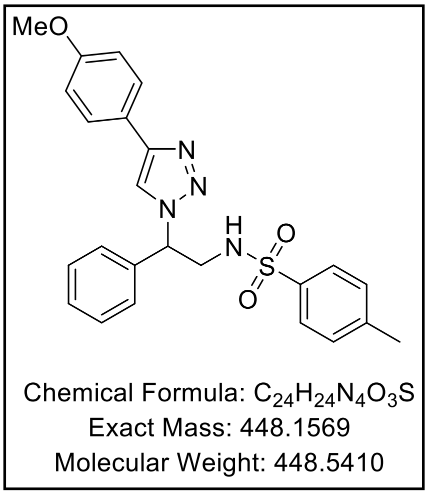

Prepared according to General Procedure A, using PhINTs (168 mg, 0.45 mmol, 1.5 equiv.), styrene (35.0 µL, 0.30 mmol, 1.0 equiv.), and 1-ethynyl-4-methoxybenzene (43.5 µL, 0.33 mmol, 1.1 equiv.). The crude product was purified by column chromatography (silica gel, 20–40% acetone in hexane) to afford the title compound as a white amorphous solid (113 mg, 83%).

^{1}H NMR (500 MHz, Acetone-*d*$_6$): δ 8.30 (s, 1H), 7.80 – 7.77 (m, 2H), 7.77 – 7.74 (m, 2H), 7.42 – 7.34 (m, 7H), 7.00 – 6.97 (m, 2H), 6.97 – 6.93 (m, 1H, N*H*), 5.92 (dd, *J* = 9.5, 5.3 Hz, 1H), 4.06 (ddd, *J* = 14.1, 9.5, 6.2 Hz, 1H), 3.82 (s, 3H), 3.73 (ddd, *J* = 14.1, 6.5, 5.3 Hz, 1H), 2.39 (s, 3H).

^{13}C{^{1}H} NMR (126 MHz, Acetone-*d*$_6$): δ 160.6, 147.9, 144.2, 138.9, 138.4, 130.6, 129.8, 129.6, 128.1, 127.8, 127.6, 124.7, 120.6, 115.1, 65.8, 55.7, 47.8, 21.5.

IR (thin film): 3311, 2357, 1497, 1331, 1250, 1159, 1092, 1030, 837, 814 cm^{-1}.

HRMS (ESI): [M+H$^+$] ($C_{24}H_{25}N_4O_3S^+$) requires *m*/*z* 449.1642, found *m*/*z* 449.1646.

4-Methyl-*N*-(2-phenyl-2-(4-(pyridin-2-yl)-1*H*-1,2,3-triazol-1-yl)ethyl)benzenesulfonamide (17)

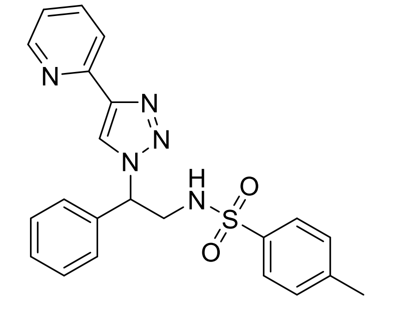

Chemical Formula: $C_{22}H_{21}N_5O_2S$
Exact Mass: 419.1416
Molecular Weight: 419.5030

Prepared according to General Procedure A, using PhINTs (171 mg, 0.45 mmol, 1.5 equiv.), styrene (35.0 µL, 0.30 mmol, 1.0 equiv.), and 2-ethynyl pyridine (34.0 µL, 0.33 mmol, 1.1 equiv.). The crude product was purified by column chromatography (silica gel, 10–20% acetone in hexane) to afford the title compound as a white amorphous solid (68 mg, 53%).

^{1}H NMR (500 MHz, Acetone-*d*$_6$): δ 8.56 (ddd, *J* = 4.8, 1.8, 1.0 Hz, 1H), 8.48 (s, 1H), 8.09 (dt, *J* = 8.0, 1.1 Hz, 1H), 7.86 (td, *J* = 7.7, 1.8 Hz, 1H), 7.78 – 7.72 (m, 2H), 7.48 – 7.42 (m, 2H), 7.42 – 7.33 (m, 5H), 7.29 (ddd, *J* = 7.5, 4.8, 1.2 Hz, 1H), 6.96 (t, *J* = 6.2 Hz, 1H, N*H*), 5.99 (dd, *J* = 9.6, 5.2 Hz, 1H), 4.12 (ddd, *J* = 14.1, 9.6, 6.3 Hz, 1H), 3.76 (ddd, *J* = 12.9, 6.3, 5.3 Hz, 1H), 2.39 (s, 3H).

^{13}C{^{1}H} NMR (126 MHz, Acetone-*d*$_6$): δ 151.7, 150.6, 149.1, 144.2, 139.0, 138.3, 137.7, 130.7, 129.9, 129.7, 128.2, 127.9, 123.7, 123.6, 120.4, 66.0, 47.7, 21.5.

IR (thin film): 3320, 1711, 1599, 1331, 1159, 1094, 765 cm^{-1}.

HRMS (ESI): [M+Na$^+$] ($C_{22}H_{21}N_5O_2SNa^+$) requires *m*/*z* 442.1308, found *m*/*z* 442.1308.

4-Methyl-*N*-(2-phenyl-2-(4-(trimethylsilyl)-1*H*-1,2,3-triazol-1-yl)ethyl)benzenesulfonamide (18)

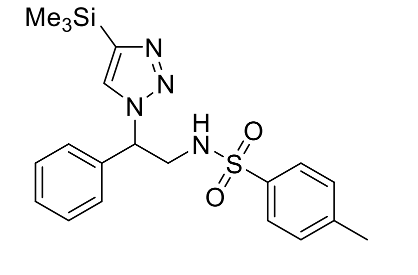

Chemical Formula: $C_{20}H_{26}N_4O_2SSi$
Exact Mass: 414.1546
Molecular Weight: 414.5990

Prepared according to General Procedure A, using PhINTs (168 mg, 0.45 mmol, 1.5 equiv.), styrene (35.0 µL, 0.30 mmol, 1.0 equiv.), and trimethylsilylacetylene (46.5 µL, 0.33 mmol, 1.1 equiv.). The crude product was purified by column chromatography (silica gel, 10–20% acetone in hexane) to afford the title compound as a white amorphous solid (89 mg, 70%).

^{1}H NMR (500 MHz, Acetone-*d*$_6$): δ 8.06 (s, 1H), 7.77 – 7.74 (m, 2H), 7.40 – 7.30 (m, 7H), 6.89 (t, *J* = 6.3 Hz, 1H, N*H*), 5.92 (dd, *J* = 9.2, 5.6 Hz, 1H), 4.01 (ddd, *J* = 14.0, 9.2, 6.3 Hz, 1H), 3.69 (dt, *J* = 14.0, 6.0 Hz, 1H), 2.41 (s, 3H), 0.26 (s, 9H).

^{13}C{^{1}H} NMR (126 MHz, Acetone-*d*$_6$): δ 146.1, 144.1, 138.9, 138.6, 130.9, 130.6, 129.8, 129.5, 128.1, 127.8, 65.3, 47.9, 21.5, –0.9.

IR (thin film): 3312, 2955, 1456, 1333, 1250, 1159, 1094, 843 cm^{-1}.

HRMS (ESI): [M+Na$^+$] ($C_{20}H_{26}N_4O_2SSiNa^+$) requires *m*/*z* 437.1438, found *m*/*z* 437.1436.

N-(2-(4-Butyl-1*H*-1,2,3-triazol-1-yl)-2-phenylethyl)-4-methylbenzenesulfonamide (19)

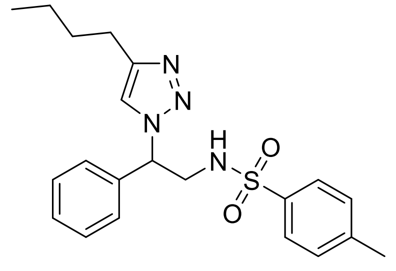

Chemical Formula: $C_{21}H_{26}N_4O_2S$
Exact Mass: 398.1776
Molecular Weight: 398.5250

Prepared according to General Procedure A, using PhINTs (171 mg, 0.45 mmol, 1.5 equiv.), styrene (35.0 µL, 0.30 mmol, 1.0 equiv.), and 1-hexyne (38.6 µL, 0.33 mmol, 1.1 equiv.). The crude product was purified by column chromatography (silica gel, 20–40% EtOAc in hexane) to afford the title compound as a white amorphous solid (100 mg, 82%).

Also prepared at gram-scale according to General Procedure A, using PhINTs (1.68 g, 4.5 mmol, 1.5 equiv.), styrene (345 µL, 3.0 mmol, 1.0 equiv.), and 1-hexyne (380 µL, 3.3 mmol, 1.1 equiv.). The crude product was purified by column chromatography (silica gel, 20–40% EtOAc in hexane) to afford the title compound as a white amorphous solid (1.07 g, 90%).

^{1}H NMR (500 MHz, Acetone-*d*$_6$): δ 7.79 – 7.72 (m, 3H), 7.40 – 7.36 (m, 2H), 7.36 – 7.27 (m, 5H), 6.91 (t, *J* = 6.3 Hz, 1H, N*H*), 5.84 (dd, *J* = 9.3, 5.5 Hz, 1H), 3.98 (ddd, *J* = 14.0, 9.3, 6.3 Hz, 1H), 3.66 (ddd, *J* = 14.0, 6.4, 5.5 Hz, 1H), 2.68 – 2.59 (m, 2H), 2.41 (s, 3H), 1.64 – 1.55 (m, 2H), 1.39 – 1.31 (m, 2H), 0.90 (t, *J* = 7.4 Hz, 3H).

^{13}C{^{1}H} NMR (126 MHz, Acetone-*d*$_6$): δ 148.4, 144.1, 138.8, 138.6, 130.5, 129.7, 129.4, 127.9, 127.7, 122.2, 65.3, 47.7, 32.2, 25.9, 22.9, 21.4, 14.1.

IR (thin film): 3305, 2930, 1456, 1333, 1159, 1094, 814 cm^{-1}.

HRMS (ESI): [M+H$^+$] ($C_{21}H_{27}N_4O_2S^+$) requires *m*/*z* 399.1849, found *m*/*z* 399.1863.

1-(2-((4-Methylphenyl)sulfonamido)-1-phenylethyl)-1*H*-1,2,3-triazole-4-carboxamide (20)

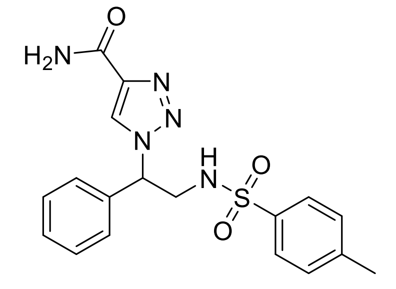

Chemical Formula: $C_{18}H_{19}N_5O_3S$
Exact Mass: 385.1209
Molecular Weight: 385.4420

Prepared according to General Procedure A, using PhINTs (171 mg, 0.46 mmol, 1.5 equiv.), styrene (35.0 µL, 0.30 mmol, 1.0 equiv.), and propiolamide (23.2 mg, 0.33 mmol, 1.1 equiv.). The crude product was purified by column chromatography (silica gel, 30–100% acetone in hexane) to afford the title compound as a white amorphous solid (87 mg, 74%).

^{1}H NMR (500 MHz, DMSO-*d*$_6$): δ 8.75 (s, 1H), 8.04 (t, *J* = 6.0 Hz, 1H, N*H*), 7.84 (s, 1H, N*H*$_2$), 7.69 – 7.64 (m, 2H), 7.47 (s, 1H, N*H*$_2$), 7.40 – 7.34 (m, 7H), 5.87 (dd, *J* = 9.7, 5.3 Hz, 1H), 3.87 (ddd, *J* = 13.9, 9.7, 6.4 Hz, 1H), 3.50 – 3.37 (m, 1H), 2.37 (s, 3H).

^{13}C{^{1}H} NMR (126 MHz, DMSO-*d*$_6$): δ 161.5, 143.1, 143.0, 137.2, 136.7, 129.8, 129.0, 128.8, 127.2, 126.5, 126.2, 64.5, 45.9, 21.0.

IR (thin film): 3273, 1653, 1339, 1155, 1092, 814 cm^{-1}.

HRMS (ESI): [M+Na$^+$] ($C_{18}H_{19}N_5O_3SNa^+$) requires *m*/*z* 408.1101, found *m*/*z* 408.1098.

4-Nitro-*N*-(2-phenyl-2-(4-phenyl-1*H*-1,2,3-triazol-1-yl)ethyl)benzenesulfonamide (21)

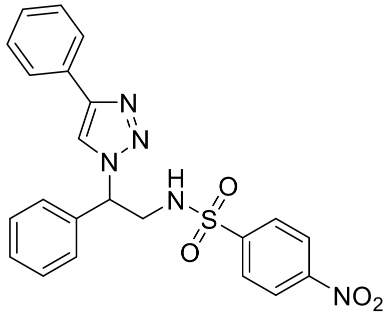

Chemical Formula: $C_{22}H_{19}N_5O_4S$
Exact Mass: 449.1158
Molecular Weight: 449.4850

Prepared according to General Procedure A, using 4-nitro-*N*-(phenyl-γ[3]-iodaneylidene)benzenesulfonamide (185 mg, 0.45 mmol, 1.5 equiv.), styrene (35.0 μL, 0.30 mmol, 1.0 equiv.), and phenylacetylene (37.0 μL, 0.33 mmol, 1.1 equiv.). The crude product was purified by column chromatography (silica gel, 10–30% EtOAc in hexane) to afford the title compound as a white amorphous solid (106 mg, 78%).

^{1}H NMR (500 MHz, Acetone-*d*$_6$): δ 8.43 (s, 1H), 8.39 – 8.33 (m, 2H), 8.13 – 8.09 (m, 2H), 7.83 (dd, *J* = 7.9, 1.4 Hz, 2H), 7.46 – 7.29 (m, 9H), 5.96 (dd, *J* = 9.7, 5.1 Hz, 1H), 4.23 (ddd, *J* = 14.3, 9.6, 4.7 Hz, 1H), 3.91 (dd, *J* = 14.1, 4.9 Hz, 1H).

^{13}C{^{1}H} NMR (126 MHz, Acetone-*d*$_6$): δ 150.9, 148.0, 147.5, 138.0, 131.9, 129.9, 129.7, 129.7, 129.1, 128.8, 128.1, 126.2, 125.3, 121.5, 65.8, 47.7.

IR (thin film): 3105, 2359, 1529, 1348, 1312, 1163, 1092, 854 cm^{-1}.

HRMS (ESI): [M+H$^+$] ($C_{22}H_{20}N_5O_4S^+$) requires *m*/*z* 450.1231, found *m*/*z* 450.1232.

***N*-(2-Phenyl-2-(4-phenyl-1*H*-1,2,3-triazol-1-yl)ethyl)-4-(trifluoromethyl)benzenesulfonamide (22)**

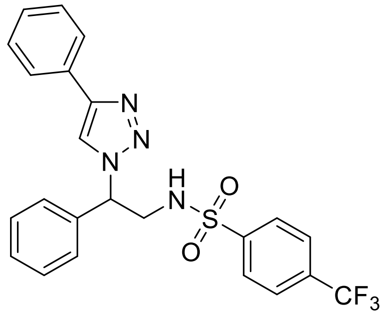

Chemical Formula: $C_{23}H_{19}F_3N_4O_2S$
Exact Mass: 472.1181
Molecular Weight: 472.4862

Prepared according to General Procedure A, using *N*-(phenyl-γ[3]-iodaneylidene)-4-(trifluoromethyl)benzenesulfonamide (196 mg, 0.45 mmol), styrene (35.0 μL, 0.30 mmol, 1.0 equiv.), and phenylacetylene (37.0 μL, 0.33 mmol). The crude product was purified by column chromatography (silica gel, 10–30% acetone in hexane) to afford the title compound as a white amorphous solid (103 mg, 71%).

^{1}H NMR (500 MHz, Acetone-*d*$_6$): δ 8.45 (s, 1H), 8.09 (d, *J* = 8.2 Hz, 2H), 7.92 (d, *J* = 8.2 Hz, 2H), 7.88 – 7.85 (m, 2H), 7.45 – 7.29 (m, 9H), 5.97 (dd, *J* = 9.5, 5.3 Hz, 1H), 4.17 (ddd, *J* = 14.5, 9.4, 5.3 Hz, 1H), 3.87 (dt, *J* = 14.1, 5.6 Hz, 1H).

^{13}C{^{1}H} NMR (126 MHz, Acetone-*d*$_6$): δ 148.2, 145.9, 138.2, 134.3 (q, $^2J_{CF}$ = 32.6 Hz), 132.2, 130.0, 129.8, 128.9, 128.7, 128.2, 127.8 (q, $^1J_{CF}$ = 272.0 Hz), 127.4 (q, $^3J_{CF}$ = 3.9 Hz), 126.4, 121.7, 66.0, 47.8.

^{19}F NMR (470 MHz, Acetone-*d*$_6$): δ –63.6.

IR (thin film): 3182, 1323, 1167, 1132, 1063, 841, 766 cm^{-1}.

HRMS (ESI): [M+H$^+$] ($C_{23}H_{20}F_3N_4O_2S^+$) requires *m*/*z* 473.1254, found *m*/*z* 473.1253.

2,6-Difluoro-*N*-(2-phenyl-2-(4-phenyl-1*H*-1,2,3-triazol-1-yl)ethyl)benzenesulfonamide (23)

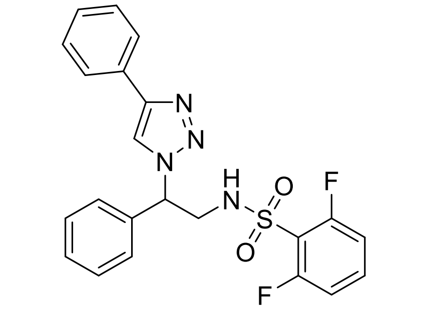

Chemical Formula: $C_{22}H_{18}F_2N_4O_2S$
Exact Mass: 440.1119
Molecular Weight: 440.4688

Prepared according to General Procedure A, using 2,6-difluoro-*N*-(phenyl-γ^3-iodaneylidene)benzenesulfonamide (181 mg, 0.45 mmol, 1.5 equiv.), styrene (35.0 µL, 0.30 mmol, 1.0 equiv.), and phenylacetylene (37.0 µL, 0.33 mmol, 1.1 equiv.). The crude product was purified by column chromatography (silica gel, 10–30% acetone in hexane) to afford the title compound as a white amorphous solid (32 mg, 24%).

^{1}H NMR (500 MHz, DMSO-d_6): δ 8.82 – 8.72 (m, 2H), 7.83 – 7.77 (m, 2H), 7.61 (ddd, *J* = 14.5, 8.5, 6.1 Hz, 1H), 7.48 – 7.30 (m, 8H), 7.18 (t, *J* = 9.2 Hz, 2H), 5.88 (dd, *J* = 9.5, 5.5 Hz, 1H), 4.10 (dd, *J* = 14.2, 9.5 Hz, 1H), 3.79 (dd, *J* = 14.2, 5.5 Hz, 1H).

^{13}C{^{1}H} NMR (126 MHz, DMSO-d_6): δ 158.5 (dd, $^1J_{CF}$ = 255.9 Hz, $^3J_{CF}$ = 3.8 Hz), 146.5, 135.1 (t, $^2J_{CF}$ = 14.7 Hz), 130.7, 128.98 (2C), 129.96, 128.8, 128.0, 127.2, 125.2, 121.0, 113.4 (dd, $^2J_{CF}$ = 23.1 Hz, $^4J_{CF}$ = 3.0 Hz), 64.3, 46.1.

^{19}F NMR (470 MHz, DMSO-d_6): δ –108.4.

IR (neat): 3383, 1614, 1468, 1161, 1204, 827, 750 cm^{-1}.

HRMS (ESI): [M+Na$^+$] ($C_{22}H_{18}F_2N_4O_2SNa^+$) requires *m*/*z* 463.1011, found *m*/*z* 463.1012.

***N*-(2-(Dibenzo[*b*,*d*]thiophen-2-yl)-2-(4-phenyl-1*H*-1,2,3-triazol-1-yl)ethyl)-4-methylbenzenesulfonamide (24)**

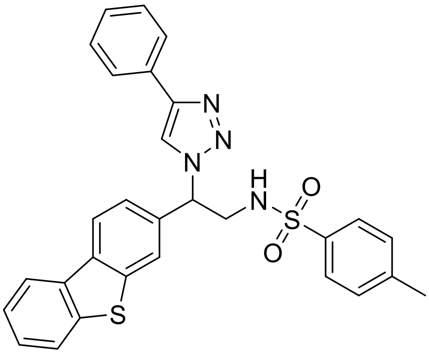

Chemical Formula: $C_{29}H_{24}N_4O_2S_2$
Exact Mass: 524.1341
Molecular Weight: 524.6570

Prepared according to General Procedure A, using PhINTs (171 mg, 0.45 mmol, 1.5 equiv.), 2-vinyldibenzo[*b*,*d*]thiophene (63.1 mg, 0.30 mmol, 1.0 equiv.), and phenylacetylene (37.0 µL, 0.33 mmol, 1.1 equiv.). The crude product was purified by column chromatography (silica gel, 20% acetone in hexane) to afford the title compound as a white amorphous solid (42 mg, 27%).

^{1}H NMR (500 MHz, DMSO-d_6): δ 8.83 (s, 1H), 8.48 (d, *J* = 1.8 Hz, 1H), 8.42 – 8.37 (m, 1H), 8.14 (t, *J* = 6.0 Hz, 1H, N*H*), 8.05 – 8.01 (m, 2H), 7.84 – 7.80 (m, 2H), 7.68 – 7.64 (m, 2H), 7.57 (dd, *J* = 8.4, 1.8 Hz, 1H), 7.56 – 7.52 (m, 2H), 7.44 (t, *J* = 7.7 Hz, 2H), 7.35 – 7.29 (m, 3H), 6.02 (dd, *J* = 8.9, 6.1 Hz, 1H), 4.00 (ddd, *J* = 14.5, 8.9, 6.2 Hz, 1H), 3.69 (dt, *J* = 13.7, 5.9 Hz, 1H), 2.28 (s, 3H).

^{13}C{^{1}H} NMR (126 MHz, DMSO-d_6): δ 146.6, 142.8, 139.01, 138.96, 137.4, 135.3, 134.6, 133.6, 130.6, 129.6, 128.9, 127.9, 127.4, 126.4, 126.3, 125.1, 124.8, 123.5, 123.1, 122.3, 121.0, 120.7, 64.2, 46.0, 20.9.

IR (thin film): 3261, 2930, 1456, 1333, 1159, 1094, 814 cm^{-1}.

HRMS (ESI): [M+Na$^+$] ($C_{29}H_{24}N_4O_2S_2Na^+$) requires *m/z* 547.1233, found *m/z* 547.1234.

N-(2-(2,6-Difluorophenyl)-2-(4-(trimethylsilyl)-1*H*-1,2,3-triazol-1-yl)ethyl)-4-nitrobenzenesulfonamide (25)

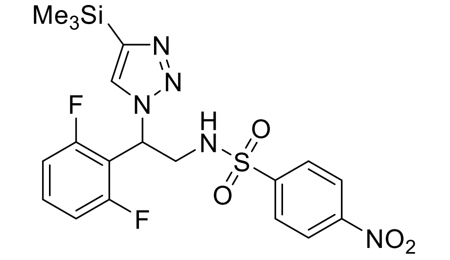

Chemical Formula: $C_{19}H_{21}F_2N_5O_4SSi$
Exact Mass: 481.1052
Molecular Weight: 481.5498

Prepared according to General Procedure A, using 4-nitro-*N*-(phenyl-γ^3-iodaneylidene)benzenesulfonamide (185 mg, 0.45 mmol, 1.5 equiv.), 1,3-difluoro-2-vinylbenzene (37.0 µL, 0.30 mmol, 1.0 equiv.), and trimethylsilylacetylene (47.0 µL, 0.33 mmol, 1.1 equiv.). The crude product was purified by column chromatography (silica gel, 15–20% acetone in hexane) to afford the title compound as a yellow oil (40 mg, 28%).

^{1}H NMR (500 MHz, Acetone-*d*$_6$): δ 8.43 – 8.40 (m, 2H), 8.15 – 8.11 (m, 2H), 8.11 (s, 1H), 7.57 – 7.51 (m, 1H, N*H*), 7.51 – 7.45 (m, 1H), 7.10 – 7.02 (m, 2H), 6.36 – 6.30 (m, 1H), 4.28 (dt, *J* = 14.1, 6.9 Hz, 1H), 4.06 (dt, *J* = 13.6, 6.3 Hz, 1H), 0.25 (s, 9H).

^{13}C{^{1}H} NMR (126 MHz, Acetone-*d*$_6$): δ 162.1 (dd, $^1J_{CF}$ = 249.7 Hz, $^3J_{CF}$ = 7.3 Hz), 151.1, 147.3, 146.4, 132.8 (t, $^3J_{CF}$ = 10.8 Hz), 130.1, 129.2, 125.4, 113.7 (t, $^2J_{CF}$ = 17.1 Hz), 113.2 (dd, $^2J_{CF}$ = 19.9 Hz, $^4J_{CF}$ = 4.5 Hz), 55.5, 45.5, –1.0.

^{19}F NMR (377 MHz, Acetone-*d*$_6$): δ –113.6.

IR (thin film): 3100, 1626, 1530, 1472, 1348, 1163, 1094, 841, 735 cm^{-1}.

HRMS (ESI): [M+H$^+$] ($C_{19}H_{22}F_2N_5O_4SSi^+$) requires *m/z* 482.1124, found *m/z* 482.1120.

N-(2-(4-(4-Methoxyphenyl)-1*H*-1,2,3-triazol-1-yl)-2-(3,4,5-trimethoxyphenyl)ethyl)-4-nitrobenzenesulfonamide (26)

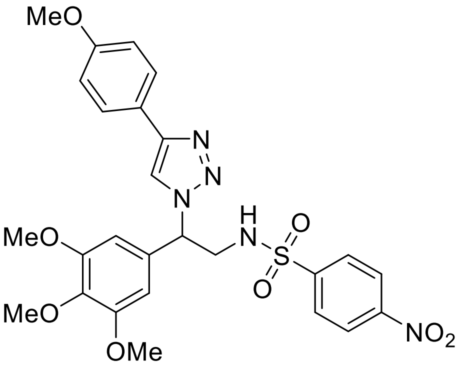

Chemical Formula: $C_{26}H_{27}N_5O_8S$
Exact Mass: 569.1580
Molecular Weight: 569.5890

Prepared according to General Procedure A, using 4-nitro-*N*-(phenyl-γ^3-iodaneylidene)benzenesulfonamide (365 mg, 0.90 mmol, 1.5 equiv.), 1,2,3-trimethoxy-5-vinylbenzene (117 mg, 0.60 mmol, 1.0 equiv.), and 4-methoxyphenylacetylene (86 µL, 0.66 mmol, 1.1 equiv.). The crude product was purified by column chromatography (silica gel, 10–30% acetone in hexane) to afford the title compound as a yellow amorphous solid (108 mg, 32%).

^{1}H NMR (500 MHz, Acetone-*d*$_6$): δ 8.37 – 8.33 (m, 2H), 8.30 (s, 1H), 8.10 – 8.06 (m, 2H), 7.77 – 7.71 (m, 2H), 7.39 (t, *J* = 6.1 Hz, 1H, N*H*), 6.99 – 6.94 (m, 2H), 6.78 (s, 2H), 5.84 (dd, *J* = 9.0, 5.8 Hz, 1H), 4.22 (ddd, *J* = 14.4, 9.0, 6.0 Hz, 1H), 3.97 – 3.91 (m, 1H), 3.82 (s, 3H), 3.78 (s, 6H), 3.68 (s, 3H).

$^{13}C\{^1H\}$ NMR (126 MHz, Acetone-*d*$_6$): δ 160.6, 154.7, 150.9, 147.9, 147.6, 139.5, 133.1, 129.1, 127.5, 125.3, 124.6, 120.4, 115.1, 105.9, 65.8, 60.5, 56.6, 55.6, 47.5.

IR (thin film): 3105, 1699, 1593, 1530, 1499, 1464, 1423, 1348, 1248, 1163, 1125, 837 cm^{-1}.

HRMS (ESI): [M+H$^+$] ($C_{26}H_{28}N_5O_8S^+$) requires *m/z* 570.1653, found *m/z* 570.1655.

Methyl 4-(1-(4-carbamoyl-1H-1,2,3-triazol-1-yl)-2-((4-methylphenyl)sulfonamido)ethyl)-2,6-dichlorobenzoate (27)

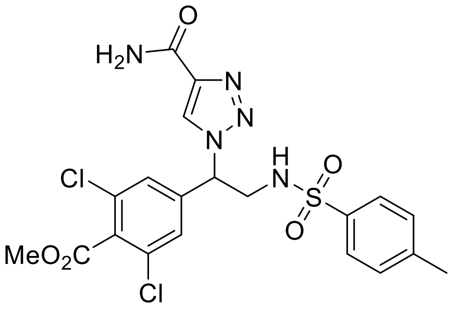

Chemical Formula: $C_{20}H_{19}Cl_2N_5O_5S$
Exact Mass: 511.0484
Molecular Weight: 512.3620

Prepared according to General Procedure A, using PhINTs (112 mg, 0.30 mmol, 1.5 equiv.), methyl 2,6-dichloro-4-vinylbenzoate (100 mg, 46% purity, 0.20 mmol, 1.0 equiv.), and propiolamide (15.2 mg, 0.22 mmol, 1.1 equiv.). The crude product was purified by column chromatography (silica gel, 1–10% MeOH in CH_2Cl_2) to afford the title compound as a white amorphous solid (42 mg, 41%).

^1H NMR (500 MHz, Acetone-*d*$_6$): δ 8.56 (s, 1H), 7.75 – 7.69 (m, 2H), 7.59 (s, 2H), 7.39 – 7.35 (m, 3H), 7.04 (t, *J* = 6.4 Hz, 1H, N*H*), 6.76 (br. s, 1H), 6.07 (dd, *J* = 8.3, 6.4 Hz, 1H), 4.09 (ddd, *J* = 14.5, 8.3, 6.4 Hz, 1H), 3.96 – 3.88 (m, 4H), 2.42 (s, 3H).

$^{13}C\{^1H\}$ NMR (126 MHz, Acetone-*d*$_6$): δ 165.2, 162.2, 144.41, 144.38, 141.9, 138.8, 132.6, 130.7, 128.6, 127.8, 127.5, 64.5, 53.6, 47.1, 21.5.

IR (thin film): 3446, 3140, 1740, 1668, 1599, 1287, 1155, 1092, 814 cm^{-1}.

HRMS (ESI): [M+H$^+$] ($C_{20}H_{20}Cl_2N_5O_5S^+$) requires *m/z* 512.0557, found *m/z* 512.0555.

5. **X-ray Crystallography**

Sample Preparation:

A sample vial was charged with amorphous solid **8** (after column chromatography purification) which was suspended in a minimal volume of an 80:20 hexane/ acetone solvent mixture. The vial was left open to air, with crystals forming through slow evaporation.

X-ray diffraction data for compound **8** were collected at 173 K using a Rigaku FR-X Ultrahigh Brilliance Microfocus RA generator/confocal optics with XtaLAB P200 diffractometer [Mo Kα radiation (λ = 0.71073 Å)]. Data were collected (using a calculated strategy) and processed (including correction for Lorentz, polarization and absorption) using CrysAlisPro.[14] Structure was solved by dual-space methods (SHEXLT[15]) and refined by full-matrix least-squares against F^2 (SHELXL-2019/3[16]). Non-hydrogen atoms were refined anisotropically, and hydrogen atoms were refined using a riding model except for the hydrogen atoms on N1 and O3 which were located from the difference Fourier map and refined isotropically subject to a distance restraint. All calculations were performed using the Olex2[17] interface. Selected crystal data: $C_{24}H_{24}N_4O_2S$, M = 432.53, monoclinic, a = 22.6748(15), b = 5.8767(4), c = 16.3771(11) Å, β = 95.621(6)°, U = 2171.8(2) Å^3, T = 173 K, space group $P2_1/c$ (no. 14), Z = 4, 39393 reflections measured, 5375 unique (R_{int} = 0.1004), which were used in all calculations. The final R_1 [$I > 2\sigma(I)$] was 0.0688 and wR_2 (all data) was 0.1201. CCDC 2465018 contains the supplementary crystallographic data for this paper. These data can be obtained free of charge from The Cambridge Crystallographic Data Centre via www.ccdc.cam.ac.uk/structures.

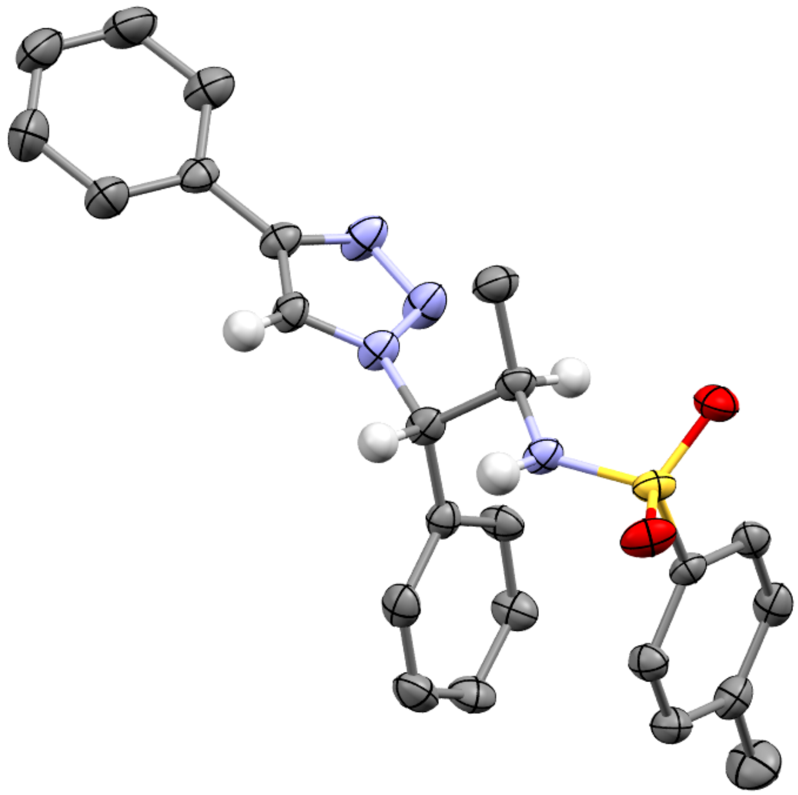

Figure S1: Asymmetric unit cell of compound **8** with 50% ellipsoids. Non-relevant hydrogen atoms have been omitted for clarity. S; Yellow.

6. References

[1] Kumaraswamy, G.; Ankamma, K.; Pitchaiah, A. Tandem Epoxide or Aziridine Ring Opening by Azide/Copper Catalyzed [3+2] Cycloaddition: Efficient Synthesis of 1,2,3-Triazolo β-Hydroxy or β-Tosylamino Functionality Motif. *J. Org. Chem.* **2007**, *72*, 9822-9825.

[2] Evans, D. A.; Faul, M. M.; Bilodeau, M. T. Copper-catalyzed aziridination of olefins by (N-(p-toluenesulfonyl)imino)phenyliodinane. *J. Org. Chem.* **1991**, *56*, 6744-6746.

[3] Hopkins, M. D.; Scott, K. A.; DeMier, B. C.; Morgan, H. R.; Macgruder, J. A.; Lamar, A. A. Formation of N-sulfonyl imines from iminoiodinanes by iodine-promoted, N-centered radical sulfonamidation of aldehydes. *Org. Biomol. Chem.* **2017**, *15*, 9209-9216.

[4] Falk, A.; Cavalieri, A.; Nichol, G. S.; Vogt, D; Schmalz, H.-G. Enantioselective Nickel-Catalyzed Hydrocyanation using Chiral Phosphine-Phosphite Ligands: Recent Improvements and Insights. *Adv. Synth. Catal.*, **2015,** *357*, 3317-3320.

[5] Li, H.; Ying G.; Wu, Y.; Liu, C.; Chuanqi C.; Chen, F.; Shi, Y.; Zhang B. σ-Alkynyl Adsorption Enables Electrocatalytic Semihydrogenation of Terminal Alkynes with Easy-Reducible/Passivated Groups over Amorphous PdSx Nanocapsules. *J. Am. Chem. Soc.* **2022**, *144*, 19456-19465.

[6] Zhang, W; Lin, S. Electroreductive Carbofunctionalization of Alkenes with Alkyl Bromides via a Radical-Polar Crossover Mechanism. *J. Am. Chem. Soc.* **2020**, *142*, 20661-20670.

[7] Parveen, N.; Sekar, G. Palladium Nanoparticle-Catalyzed Stereoselective Domino Synthesis of 3-Allylidene-2(3H)-oxindoles and 3-Allylidene-2(3H)-benzofuranones. *J. Org. Chem.* **2020**, *85*, 4682-4694.

[8] Gassmann, J.; Voss, J. Electroreduction of Organic Compounds, 36 [1]. Electroreduction of Chlorinated Methyl Benzoates. *Z. Naturforsch B.* **2008**, *63*, 1291-1299.

[9] Shahzadi, T.; Saleem, R. S. Z.; Chotana, G. A. Facile Synthesis of Halogen Decorated para-/meta-Hydroxy-benzoates by Iridium-Catalyzed Borylation and Oxidation. *Synthesis* **2018**, *50*, 4336-4342.

[10] Ong, M.; Arnold, M.; Walz, A. W.; Wahl, J. M. Stereospecific Nitrogen Insertion Using Amino Diphenylphosphinates: An Aza-Baeyer-Villiger Rearrangement, *Org. Lett.* **2022**, *24*, 6171-6175.

[11] Shehata, M. F.; Ayer, S. K.; Roizen, J. L. Iron(MCP) Complexes Catalyze Aziridination with Olefins as Limiting Reagents, *J. Org. Chem.* **2018**, *83*, 5072-5081.

[12] Iannazzo, L.; Vollhardt, K. P. C.; Malacria, M.; Aubert, C.; Gandon, V. Alkynylboronates and -boramides in Co^{I}- and Rh^{I}-Catalyzed [2+2+2] Cycloadditions: Construction of Oligoaryls through Selective Suzuki Couplings, *Eur. J. Org. Chem.* **2011**, *2011*, 3251-3374.

[13] Hao, H.; Bagnol, T.; Pucheault, M.; Schafer, L. L. Using Catalysts To Make Catalysts: Titanium-Catalyzed Hydroamination To Access *P,N*-Ligands for Assembling Catalysts in One Pot, *Org. Lett.* **2021**, *23*, 1974-1979.

[14] *CrysAlisPro* v1.171.43.109a Rigaku Oxford Diffraction, Rigaku Corporation, *Tokyo,* Japan, 2023.

[15] Sheldrick, G. M. SHELXT – Integrated space-group and crystal structure determination. *Acta Crystallogr., Sect. A: Found. Adv*. **2015**, *71*, 3-8.

[16] Sheldrick, G. M. Crystal structure refinement with SHELXL. *Acta Crystallogr., Sect. C: Struct. Chem.* **2015**, *71*, 3-8.

[17] Dolomanov, O. V.; Bourhis, L. J.; Gildea, R. J.; Howard, J. A. K.; Puschmann, H. OLEX2: a complete structure solution, refinement and analysis program. *J. Appl. Crystallogr*. **2009**, *42*, 339-341.

7. Copies of NMR Spectra

4-Methyl-*N*-(2-phenyl-2-(4-phenyl-1H-1,2,3-triazol-1-yl)ethyl)benzenesulfonamide (1)

^{1}H NMR (500 MHz, DMSO-*d*$_6$):

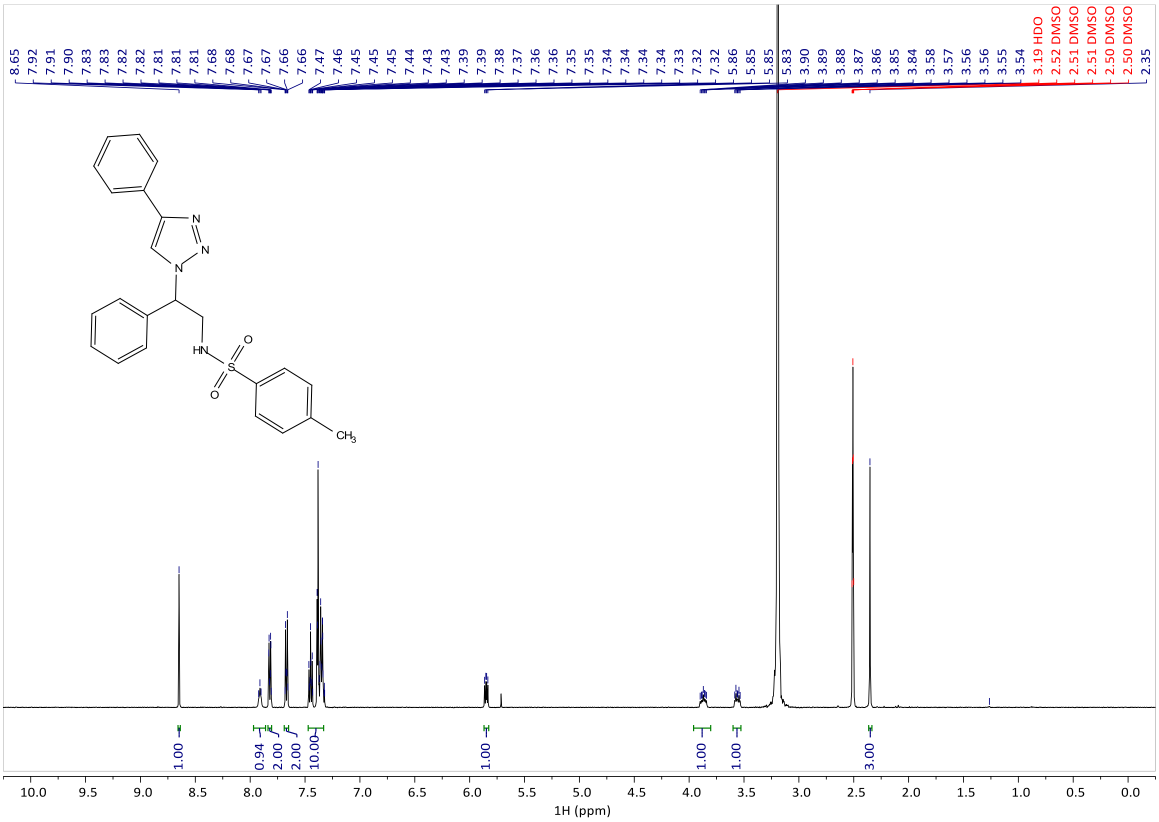

^{13}C{^{1}H} NMR (126 MHz, DMSO-*d*$_6$):

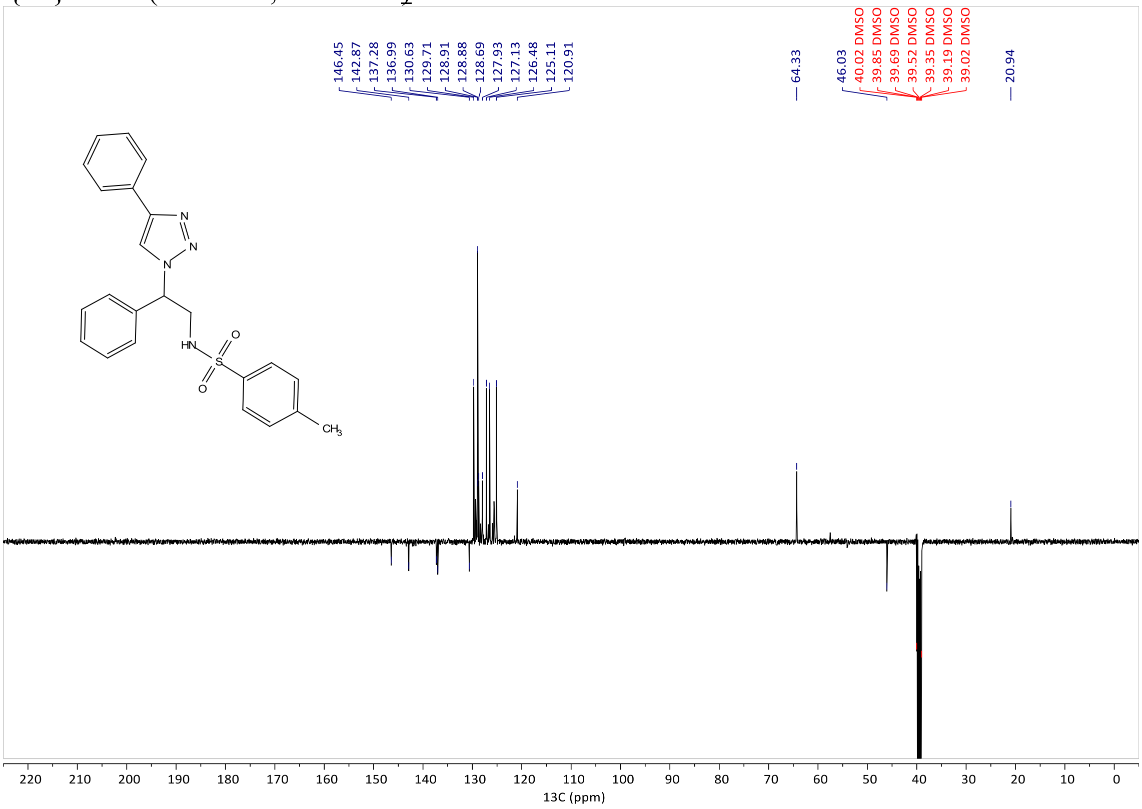

_N_-(2-(2-bromophenyl)-2-(4-phenyl-1H-1,2,3-triazol-1-yl)ethyl)-4-methylbenzenesulfonamide (2)

^{1}H NMR (500 MHz, Acetone-d_6):

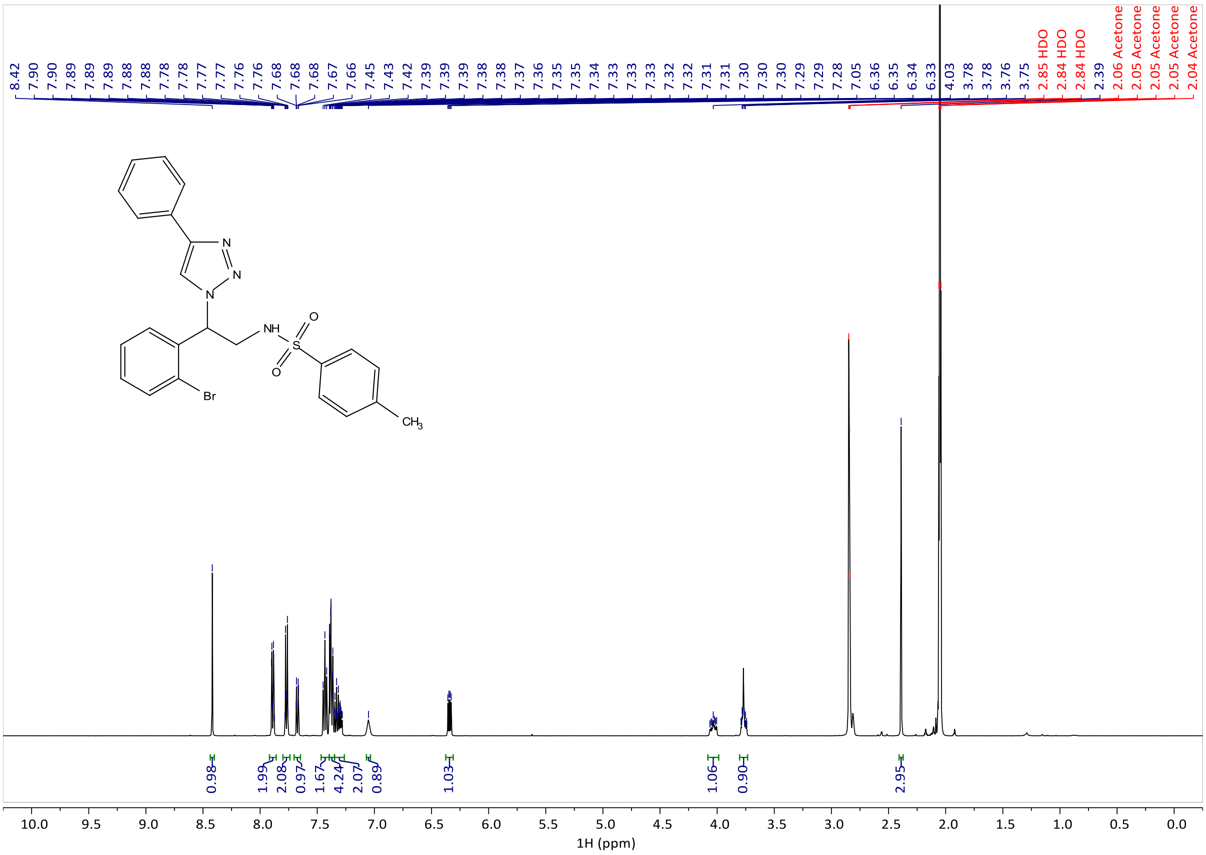

^{13}C{^{1}H} NMR (126 MHz, Acetone-d_6):

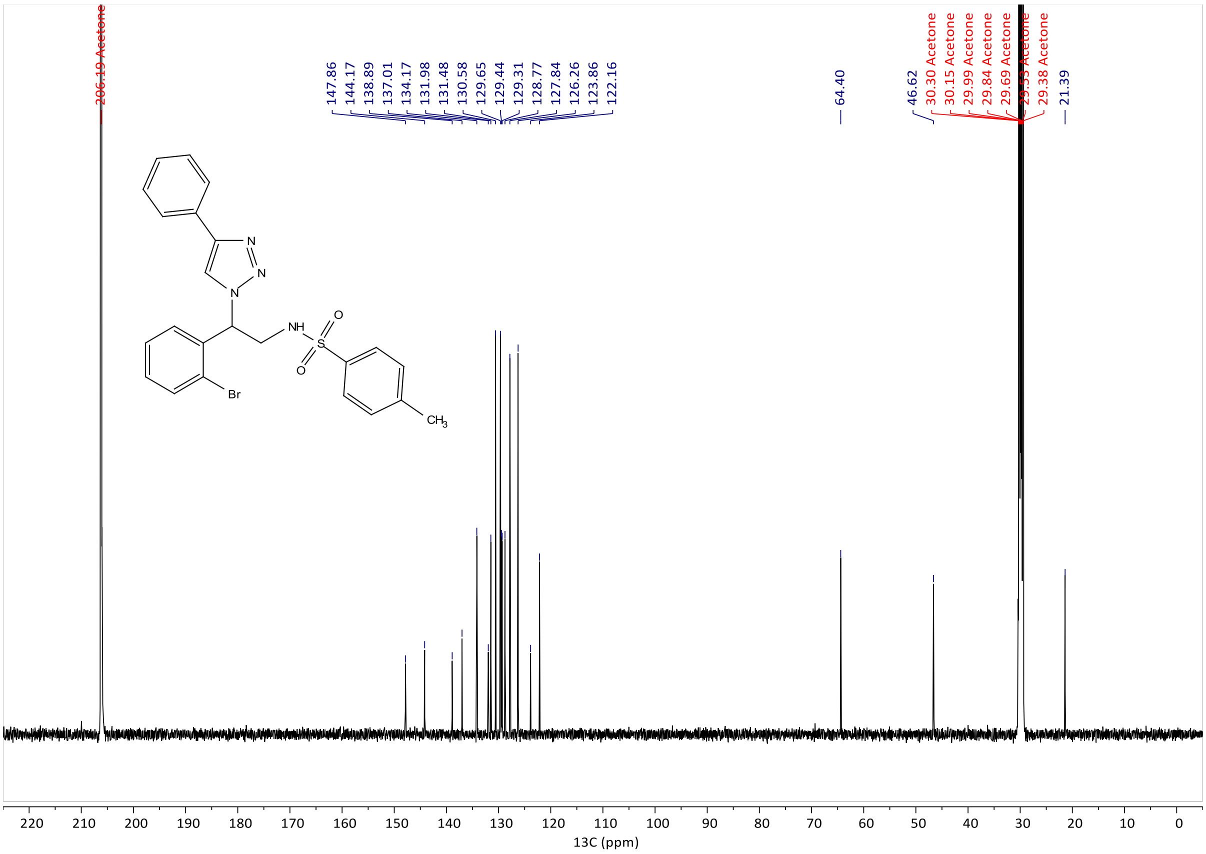

N-(2-(3-fluorophenyl)-2-(4-phenyl-1H-1,2,3-triazol-1-yl)ethyl)-4-methylbenzenesulfonamide (3)

^{1}H NMR (500 MHz, Acetone-d_6):

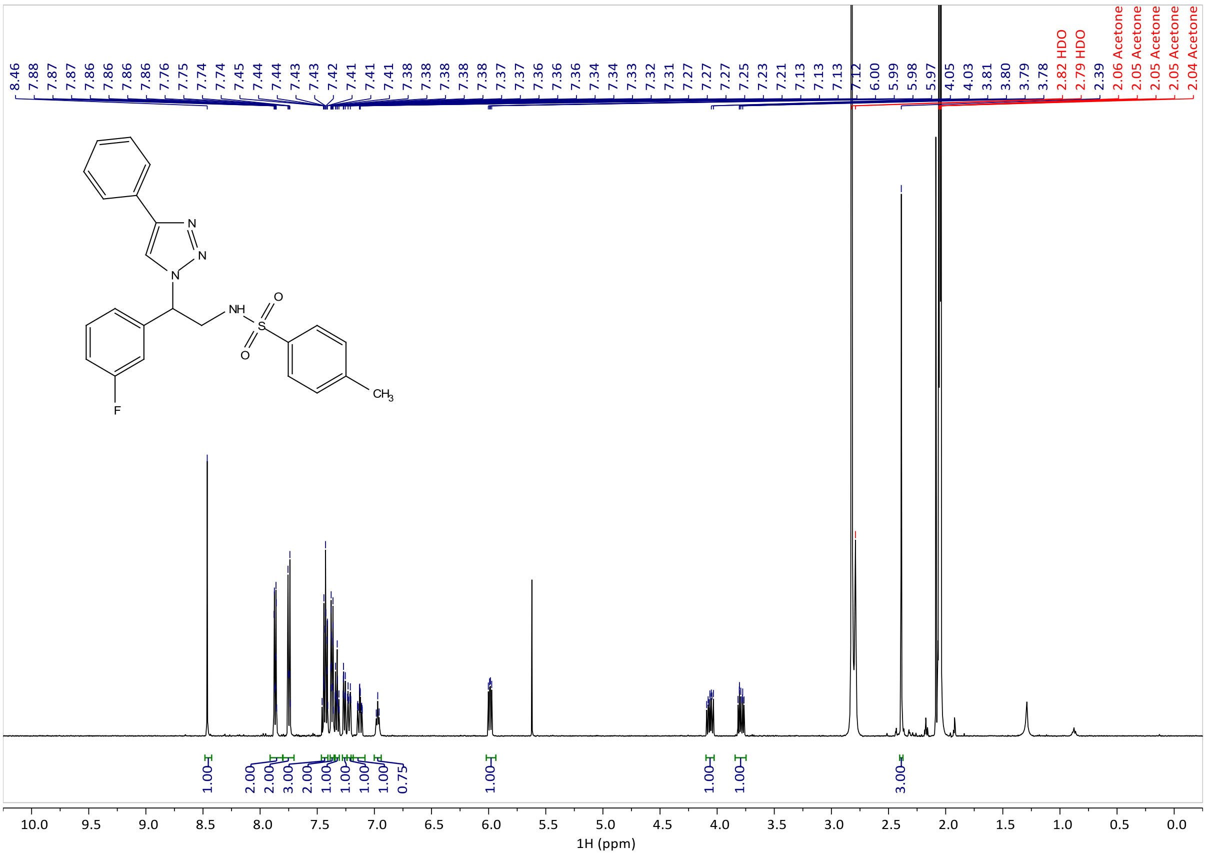

$^{13}C\{^{1}H\}$ NMR (126 MHz, Acetone-d_6):

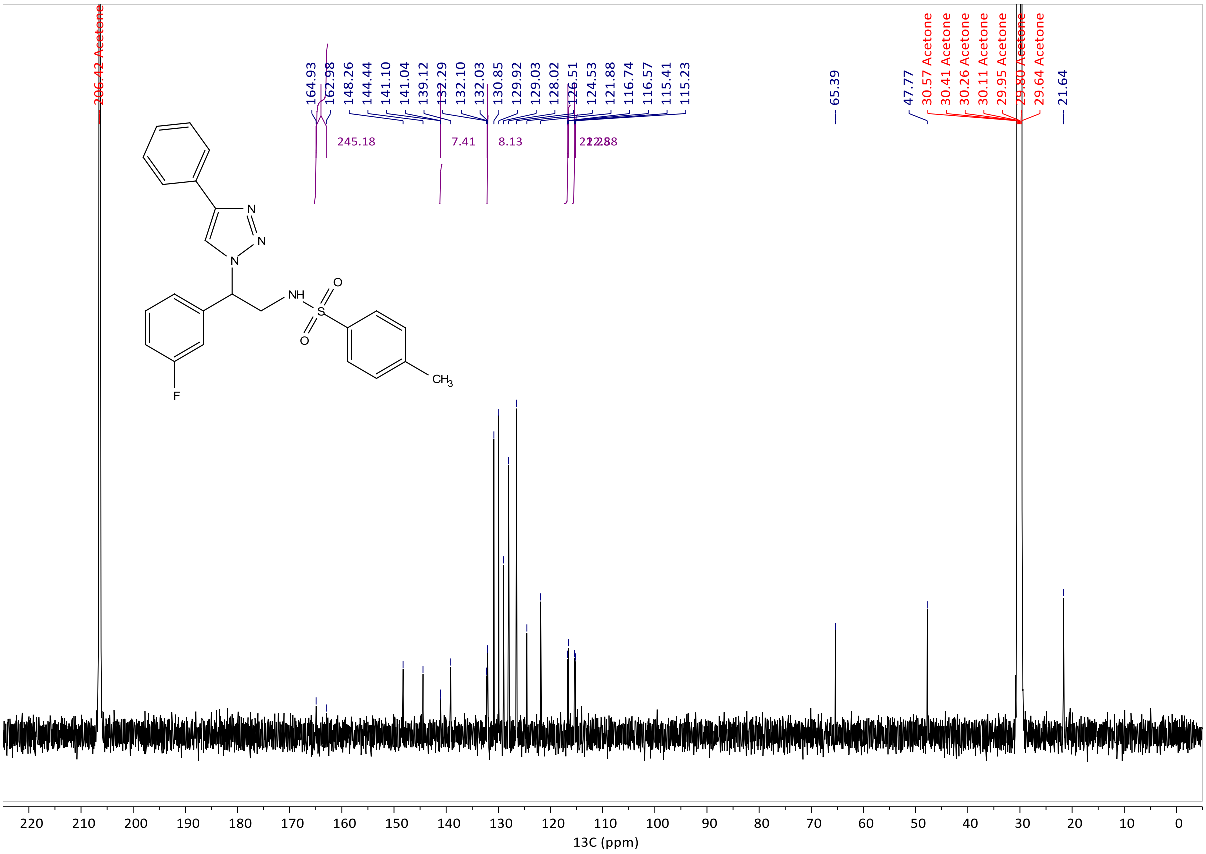

^{19}F NMR (377 MHz, Acetone-d_6):

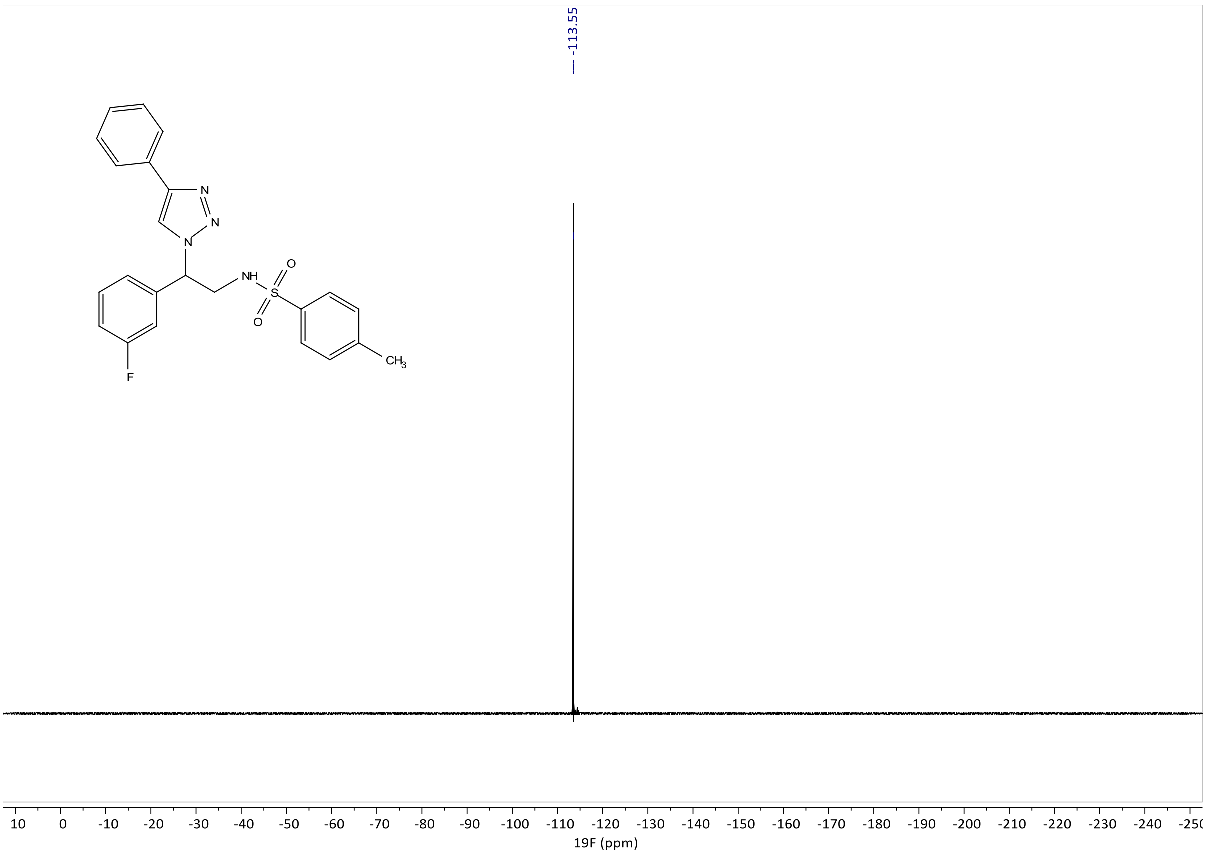

Methyl 4-(2-((4-methylphenyl)sulfonamido)-1-(4-phenyl-1H-1,2,3-triazol-1-yl)ethyl)benzoate (4)

^{1}H NMR (500 MHz, Acetone-d_6):

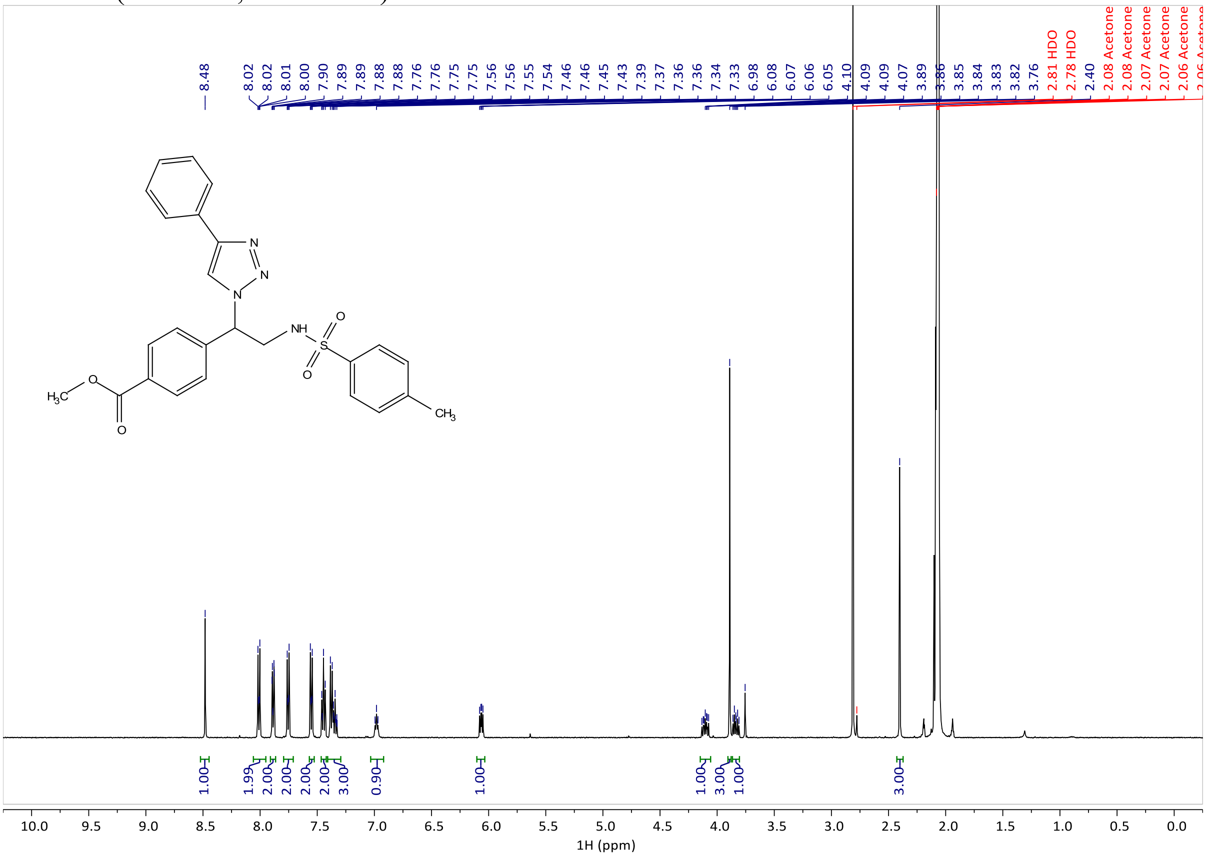

^{13}C{^{1}H} NMR (126 MHz, Acetone-d_6):

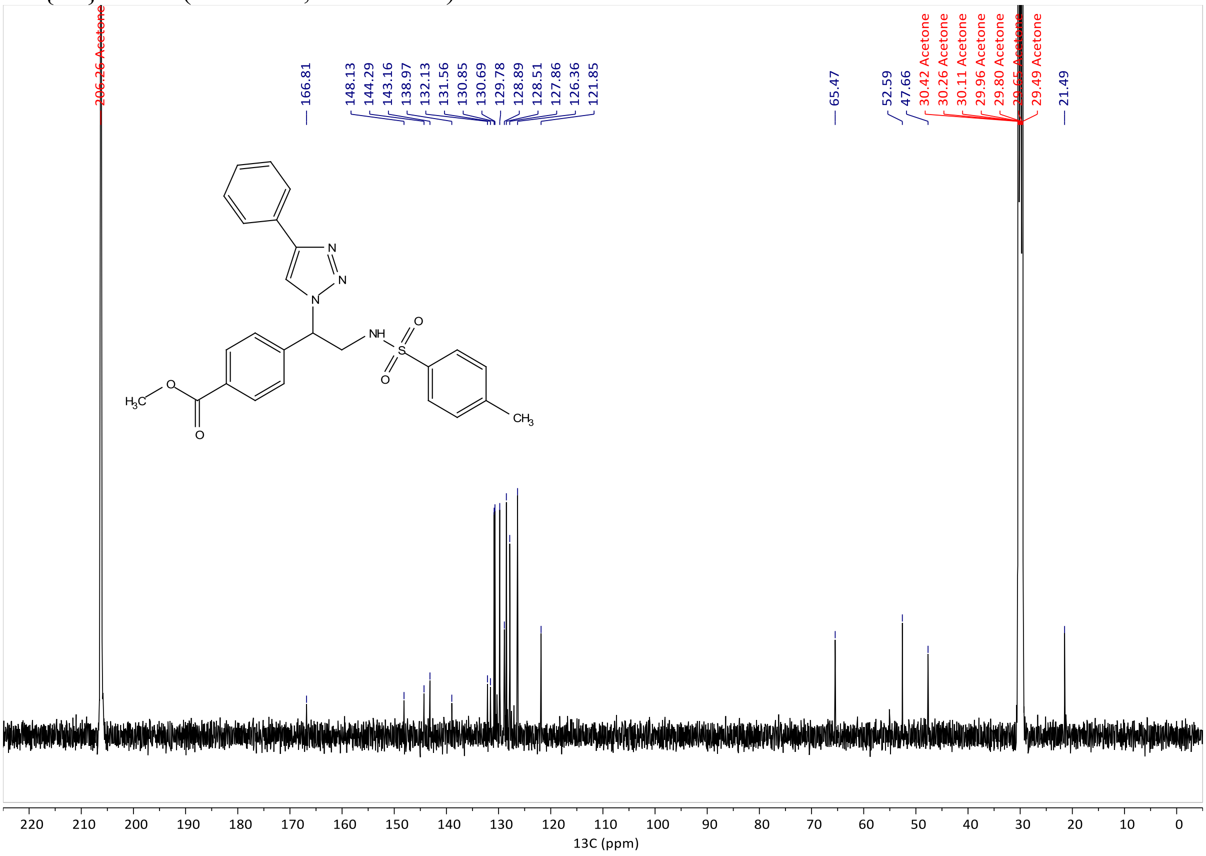

N-(2-(4-Methoxyphenyl)-2-(4-phenyl-1H-1,2,3-triazol-1-yl)ethyl)-4-methylbenzenesulfonamide (5)

^{1}H NMR (500 MHz, DMSO-d_6):

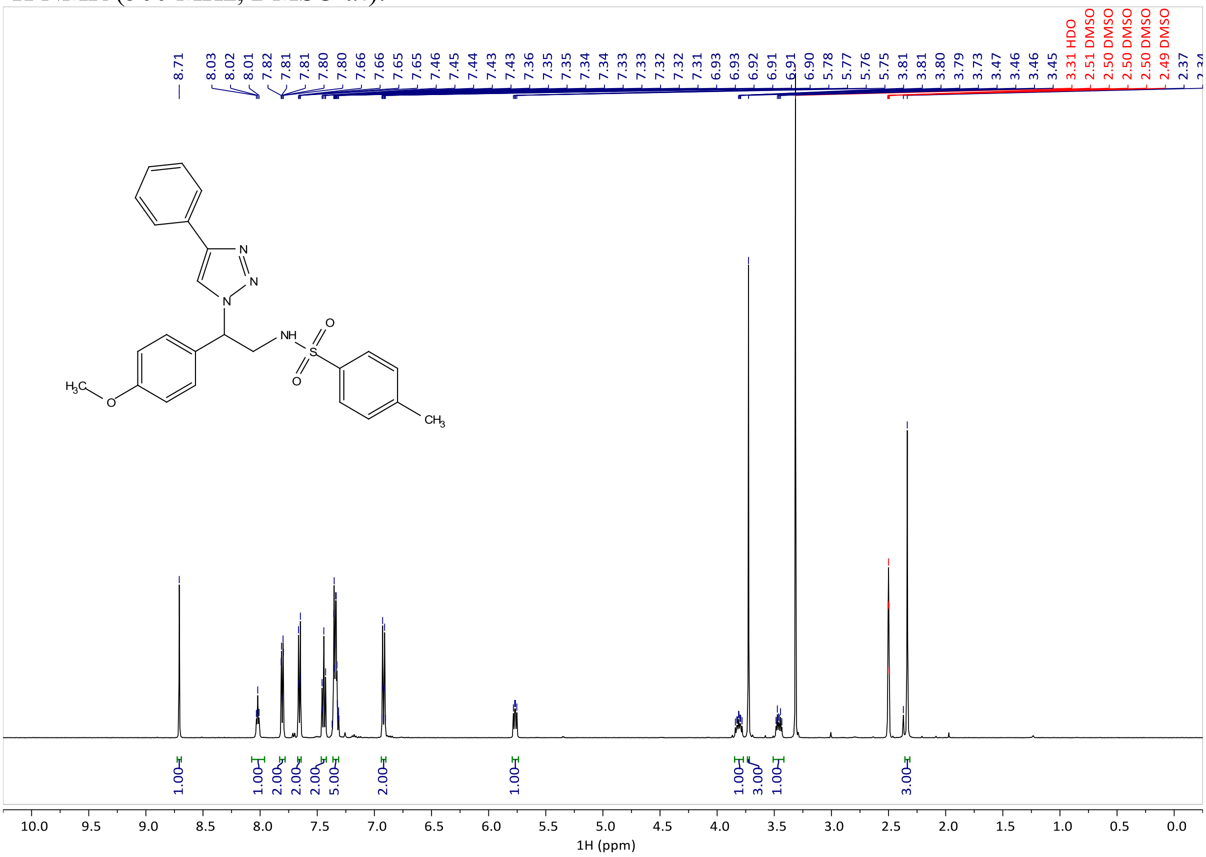

^{13}C{^{1}H} NMR (126 MHz, DMSO-d_6):

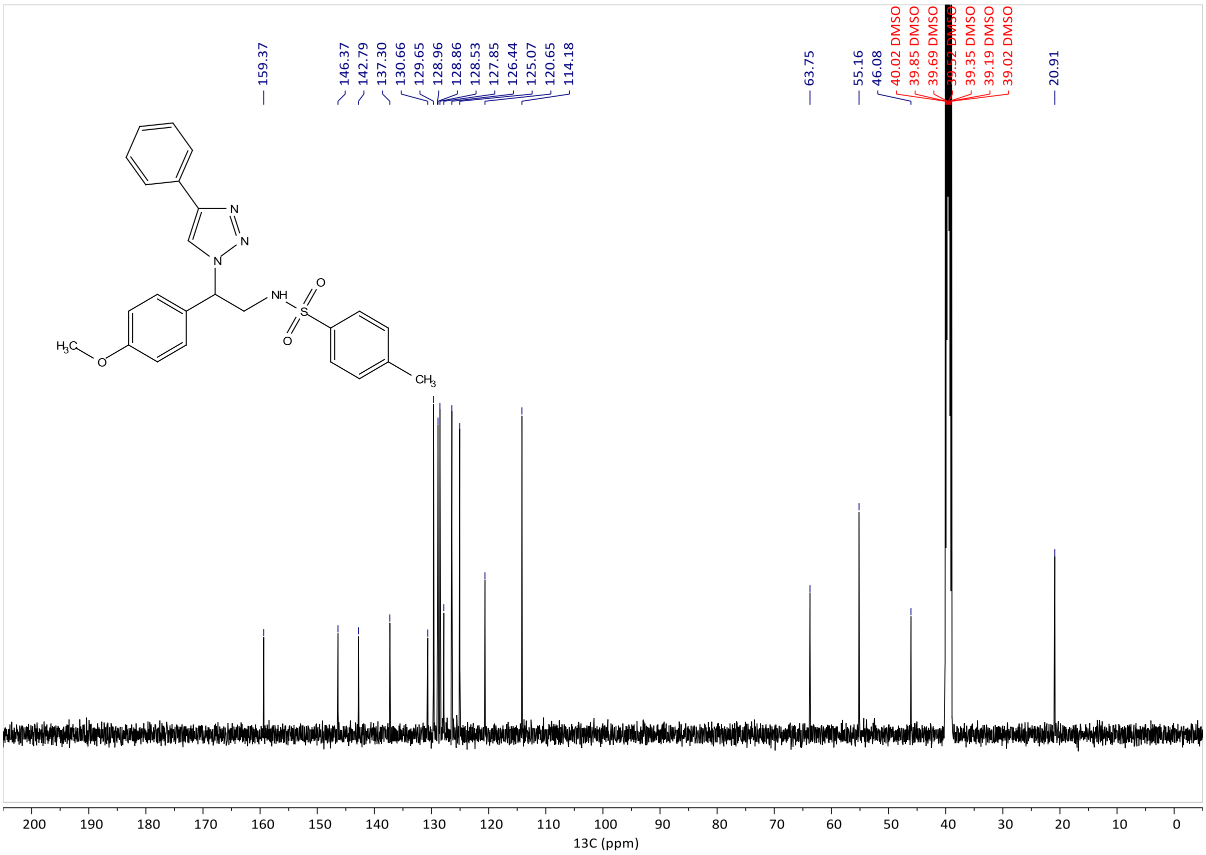

N-(2-(Benzofuran-2-yl)-2-(4-phenyl-1H-1,2,3-triazol-1-yl)ethyl)-4-methylbenzenesulfonamide (6)

^{1}H NMR (500 MHz, Acetone-d_6):

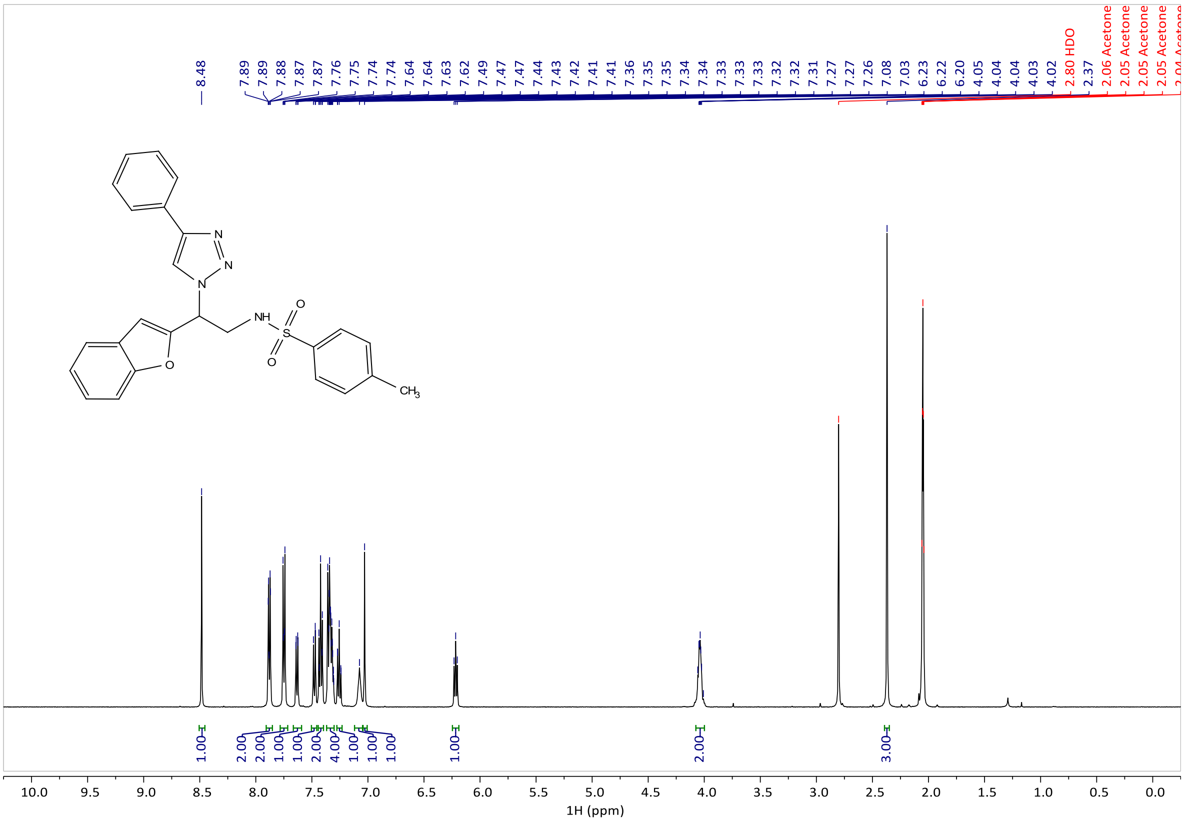

^{13}C{^{1}H} NMR (126 MHz, Acetone-d_6):

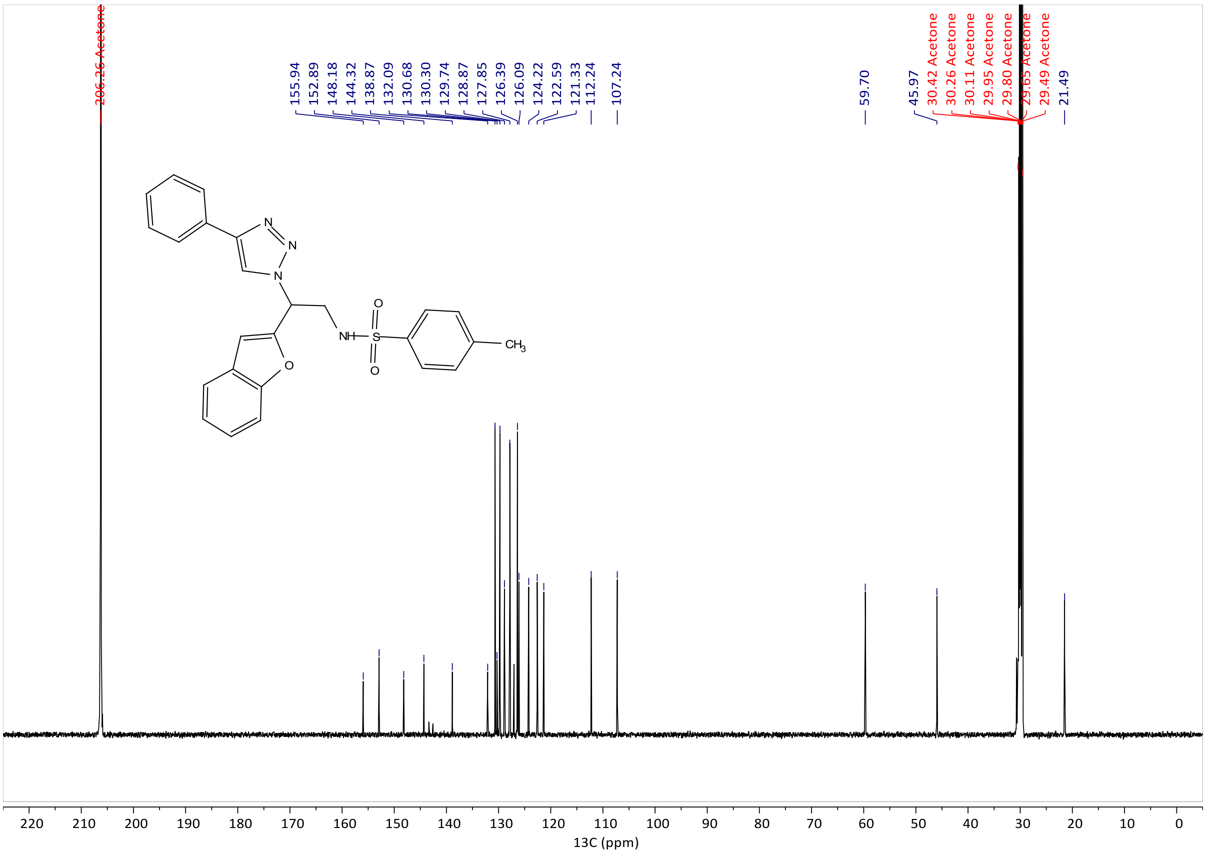

4-Methyl-*N*-(2-(4-phenyl-1H-1,2,3-triazol-1-yl)-2-(quinolin-6-yl)ethyl)benzenesulfonamide (7)

^{1}H NMR (500 MHz, DMSO-d_6):

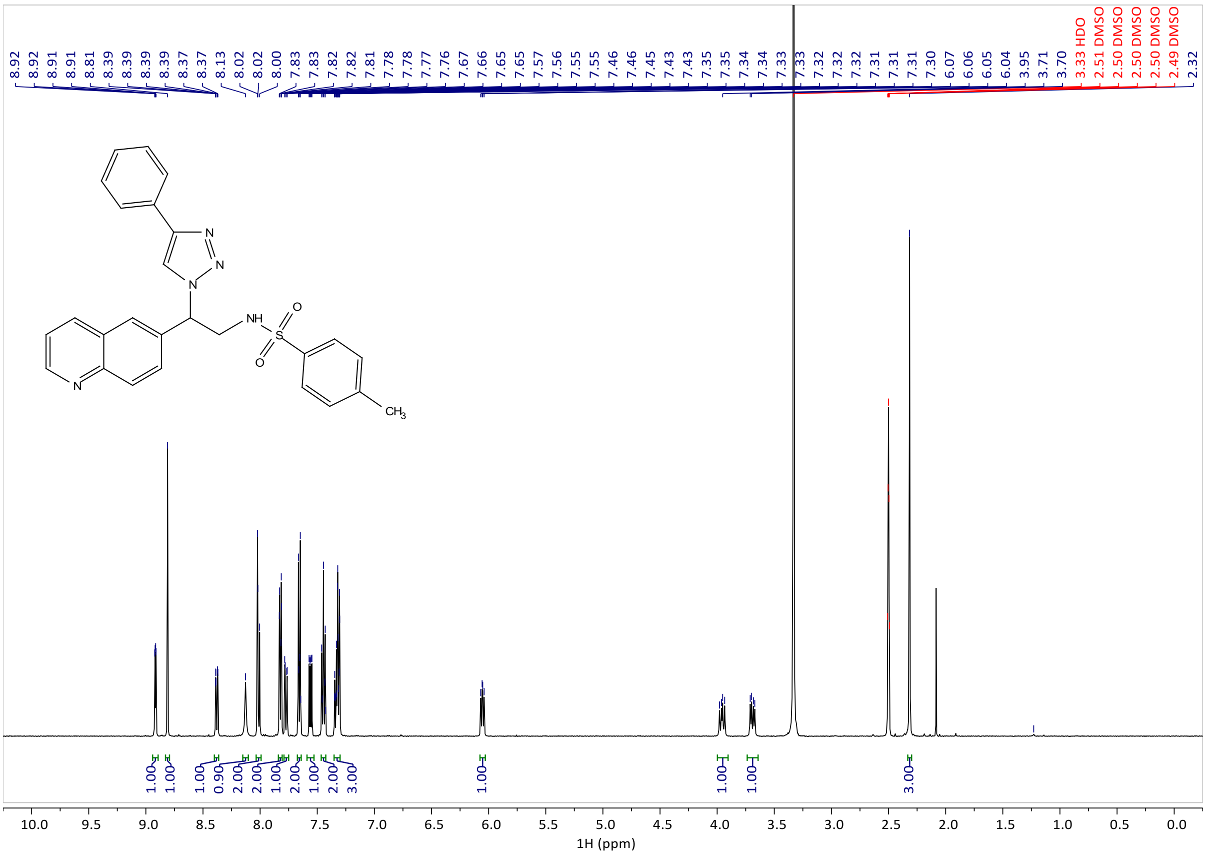

^{13}C{^{1}H} NMR (126 MHz, DMSO-d_6):

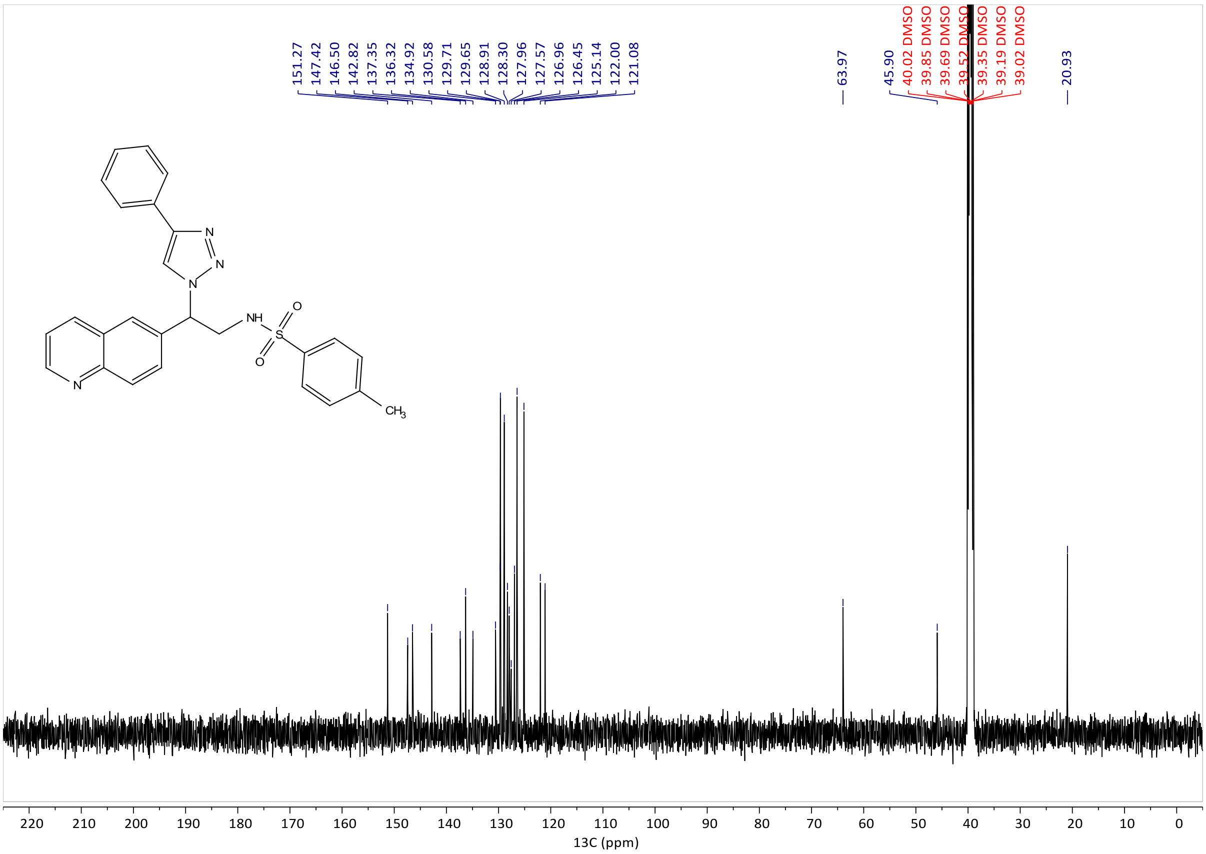

4-Methyl-*N*-(1-phenyl-1-(4-phenyl-1H-1,2,3-triazol-1-yl)propan-2-yl)benzenesulfonamide (8)

^{1}H NMR (500 MHz, Acetone-*d*$_6$):

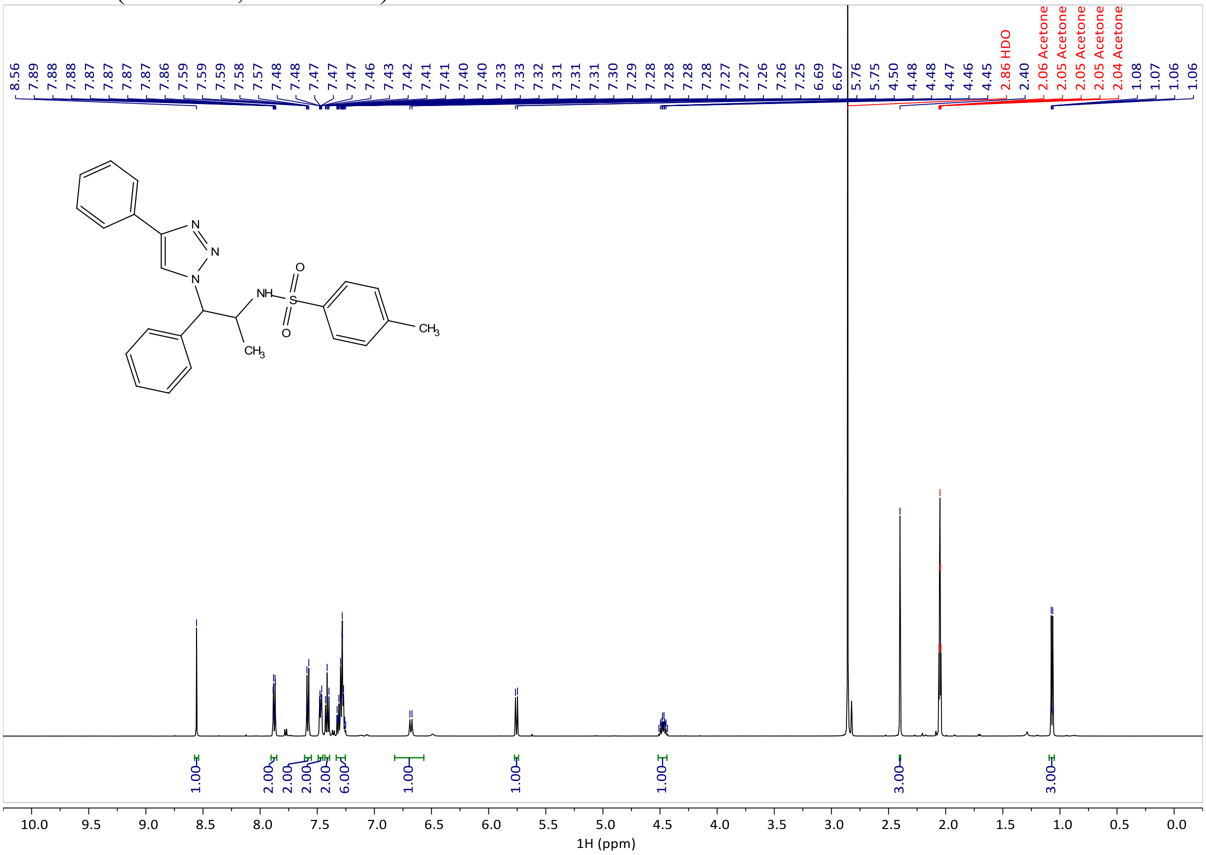

^{13}C{^{1}H} NMR (126 MHz, Acetone-*d*$_6$):

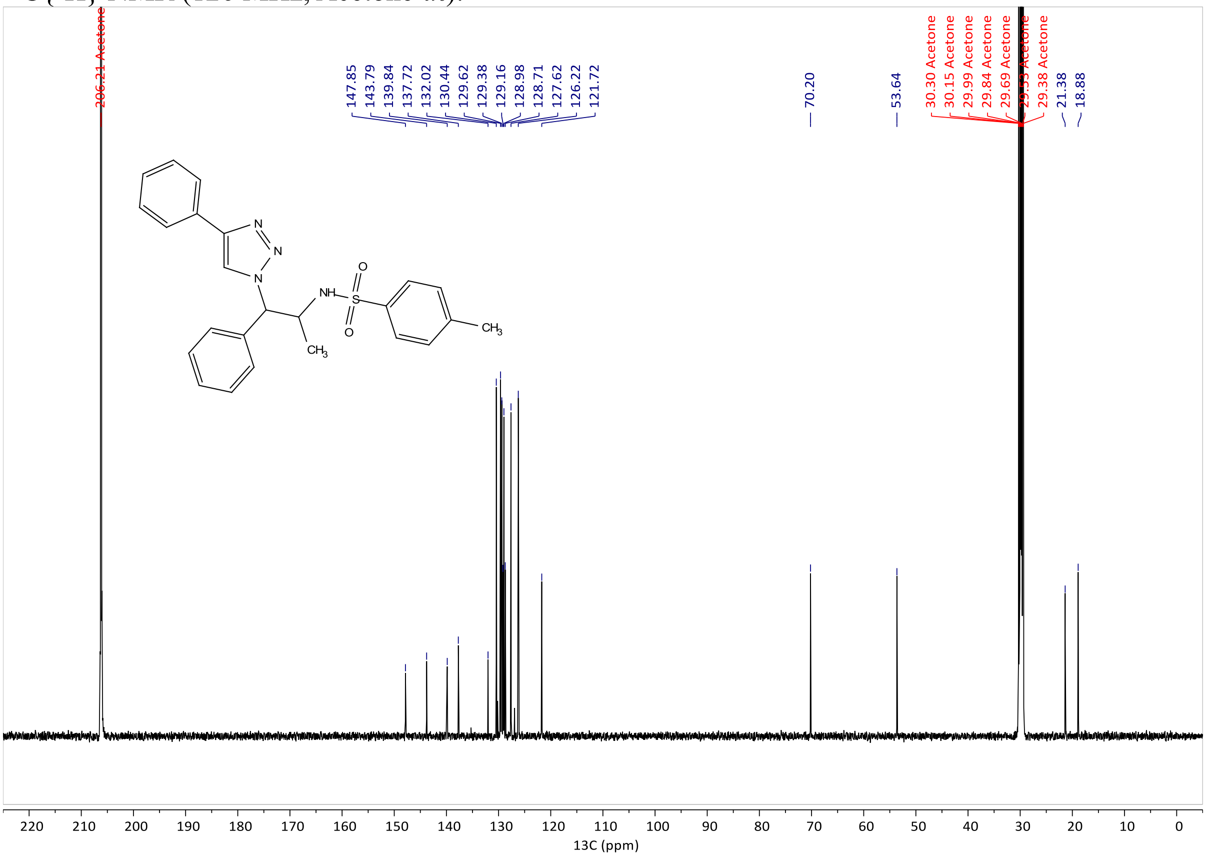

Ethyl 2-((4-methylphenyl)sulfonamido)-3-phenyl-3-(4-phenyl-1H-1,2,3-triazol-1-yl) (9)

^{1}H NMR (500 MHz, Acetone-d_6):

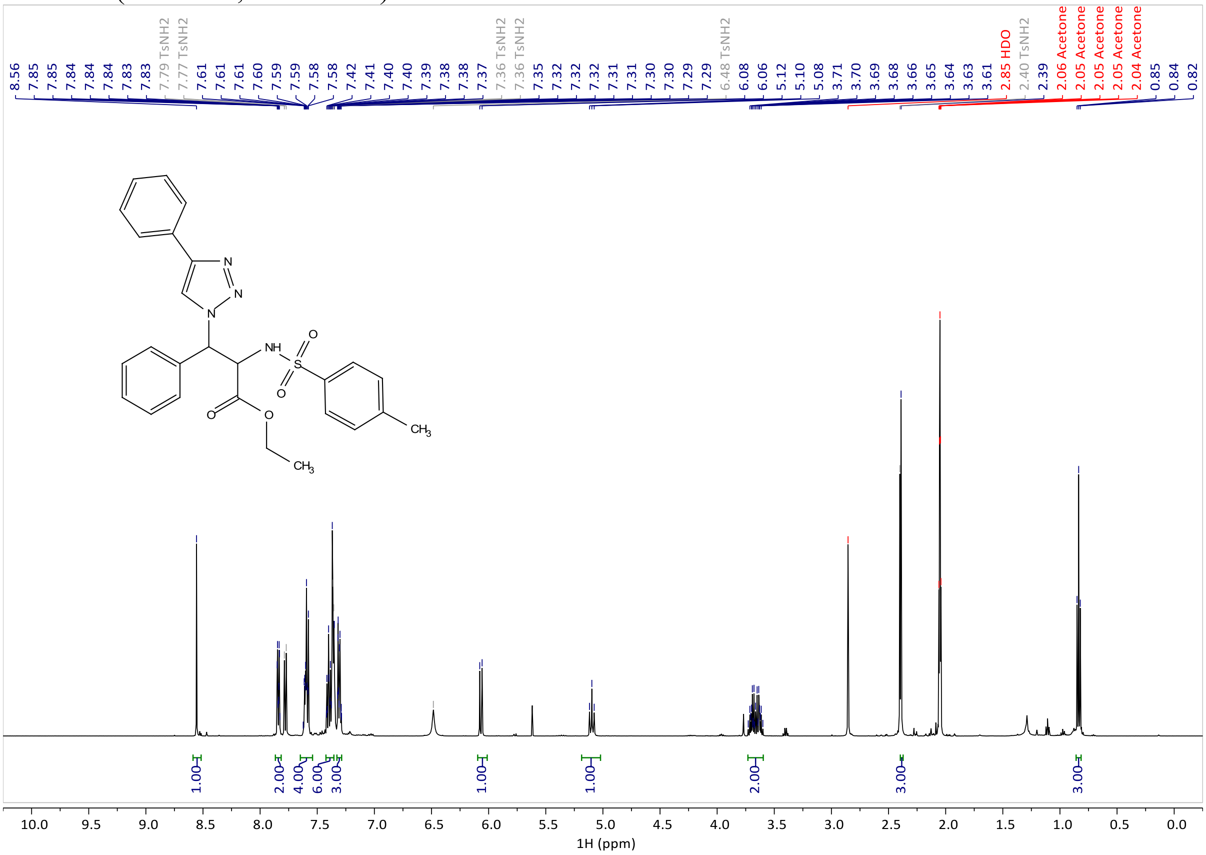

^{13}C{^{1}H} NMR (126 MHz, Acetone-d_6):

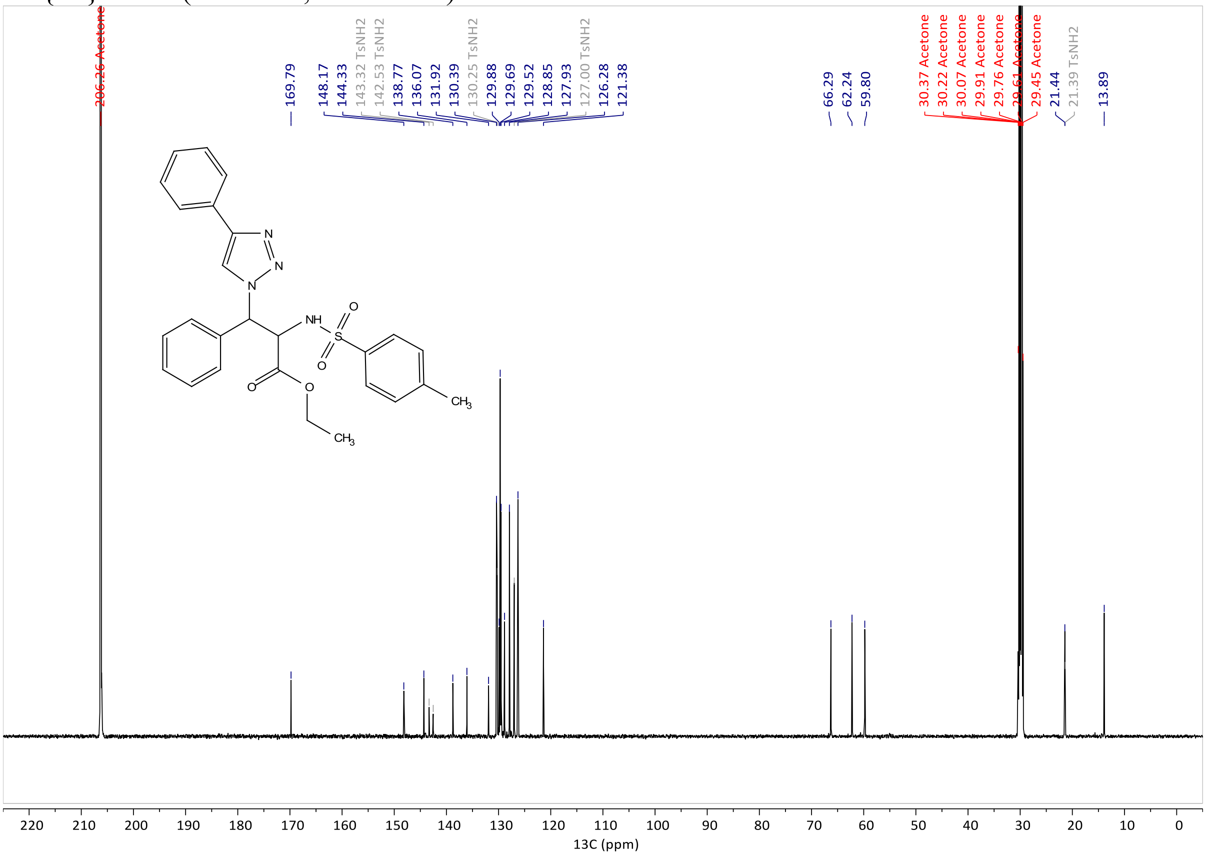

4-Methyl-*N*-(2-phenyl-2-(4-phenyl-1H-1,2,3-triazol-1-yl)propyl)benzenesulfonamide (10)

^{1}H NMR (500 MHz, Acetone-d_6):

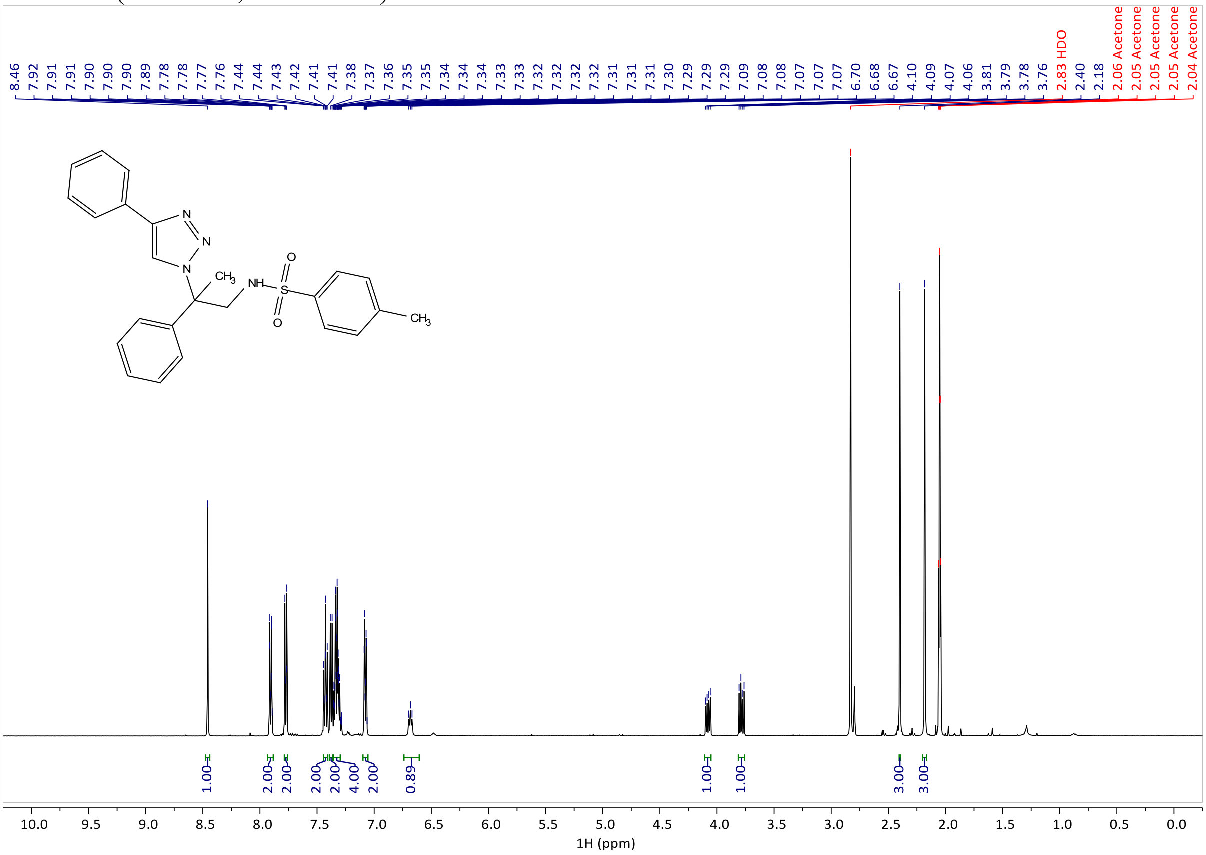

^{13}C {^{1}H} NMR (126 MHz, Acetone-d_6):

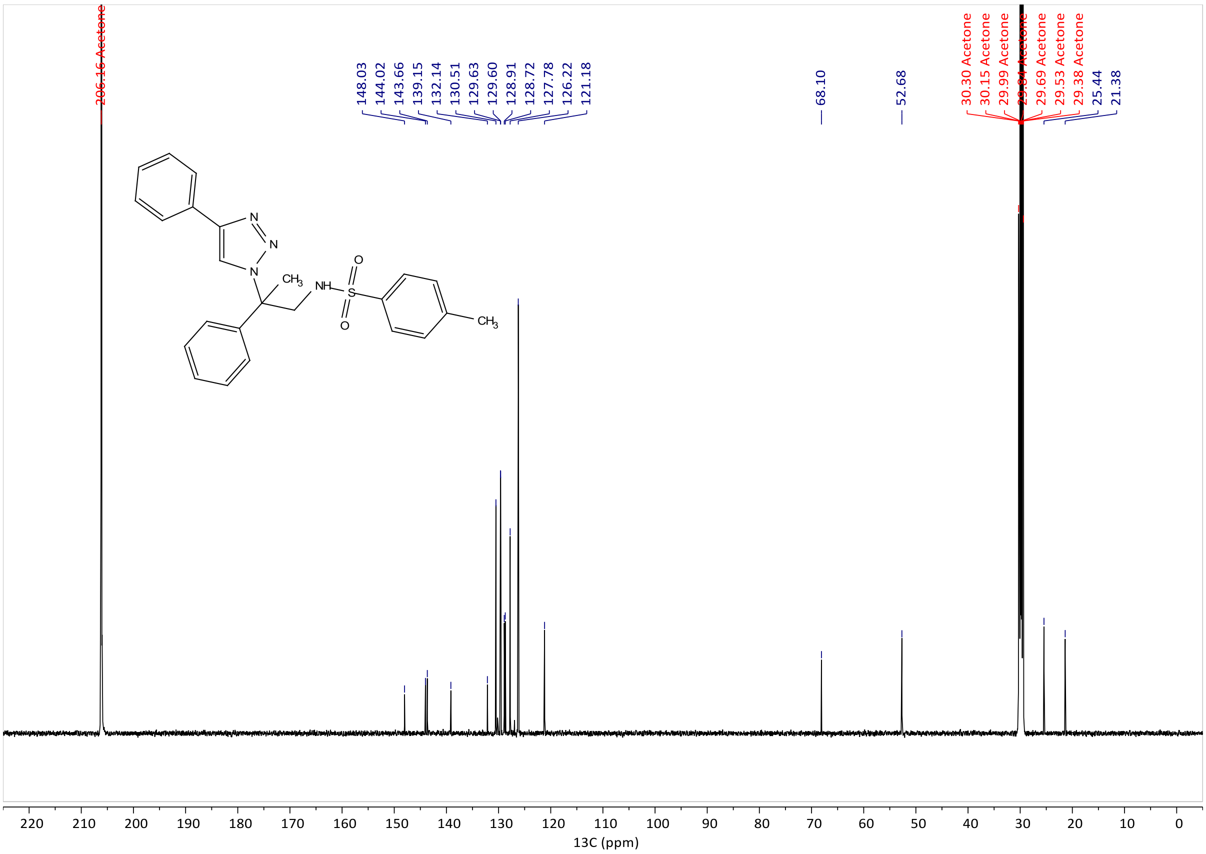

N-(2,2-Diphenyl-2-(4-phenyl-1H-1,2,3-triazol-1-yl)ethyl)-4-methylbenzenesulfonamide (11)

^{1}H NMR (500 MHz, Acetone-d_6):

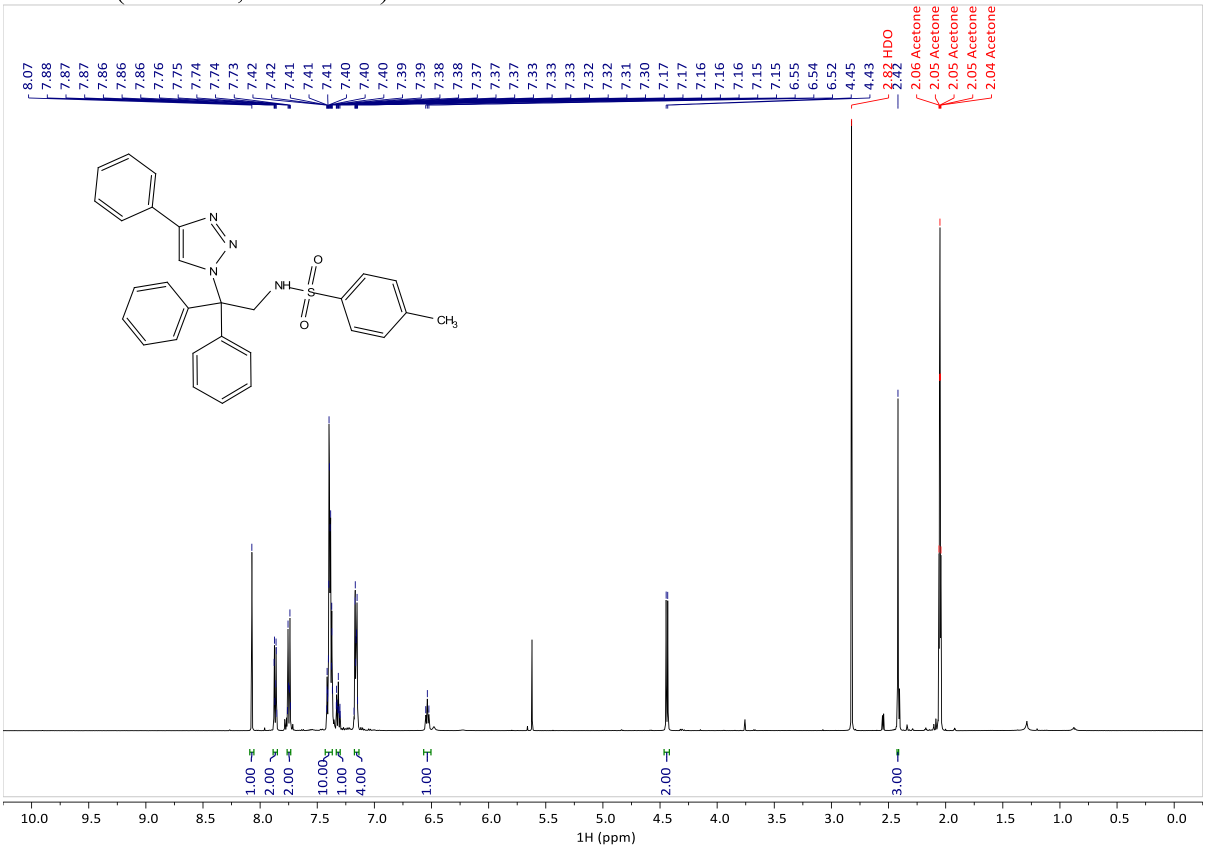

^{13}C{^{1}H} NMR (126 MHz, Acetone-d_6):

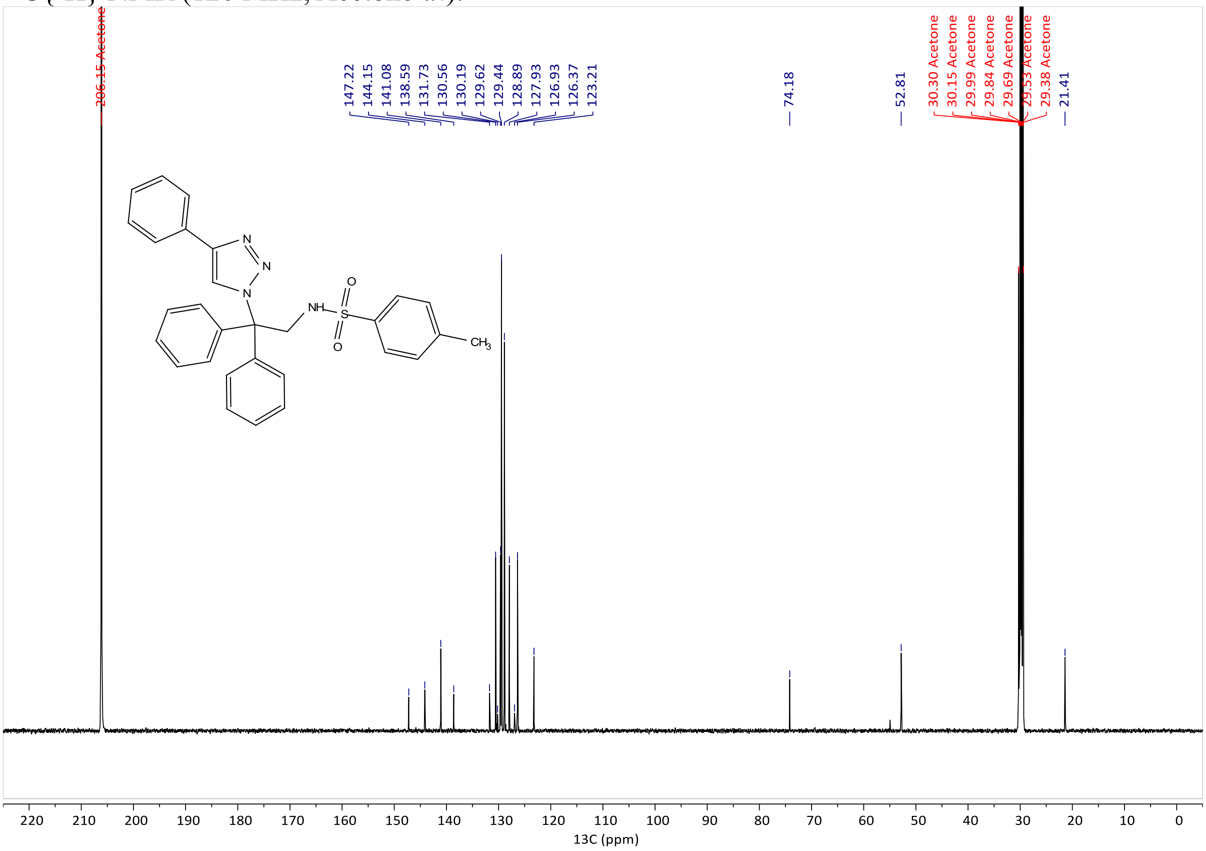

_N_-(2-(4-(2-Chlorophenyl)-1H-1,2,3-triazol-1-yl)-2-phenylethyl)-4-methylbenzenesulfonamide (12)

^{1}H NMR (500 MHz, Acetone-d_6):

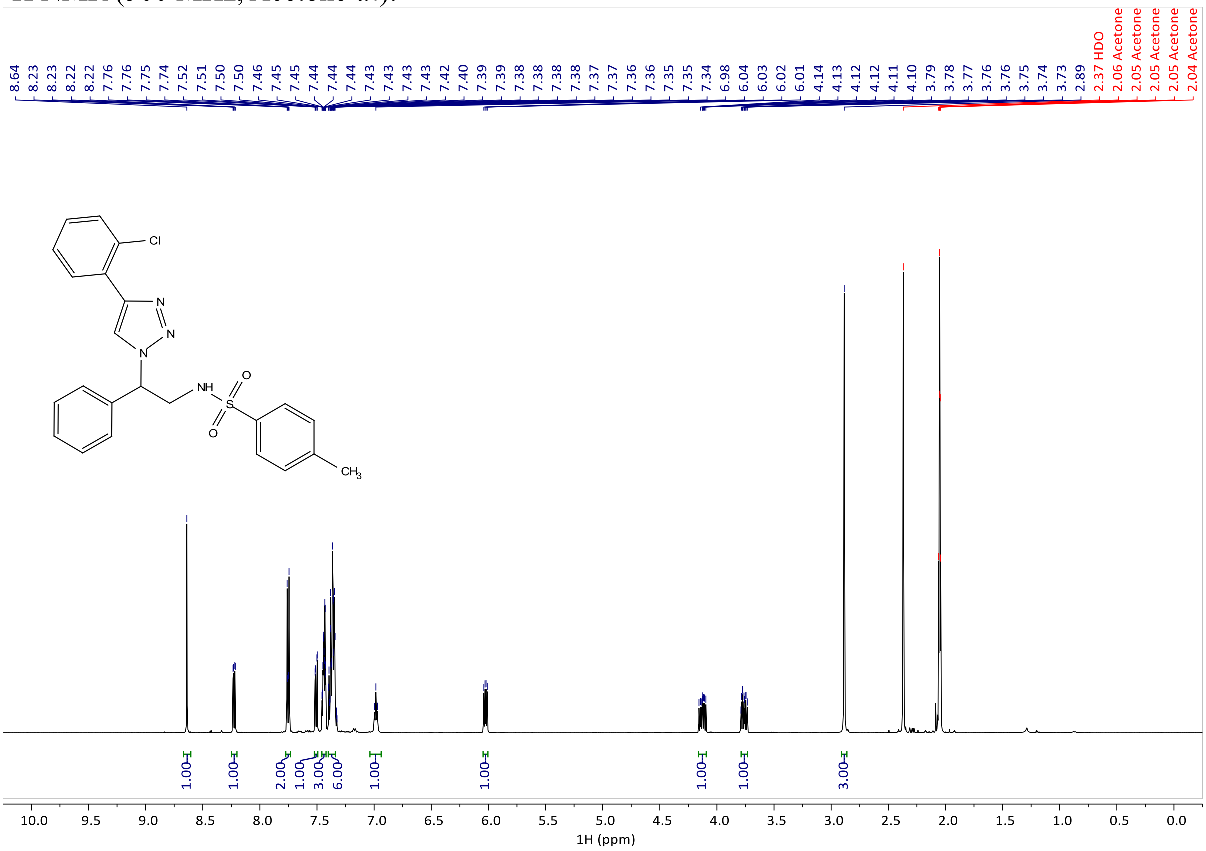

^{13}C{^{1}H} NMR (126 MHz, Acetone-d_6):

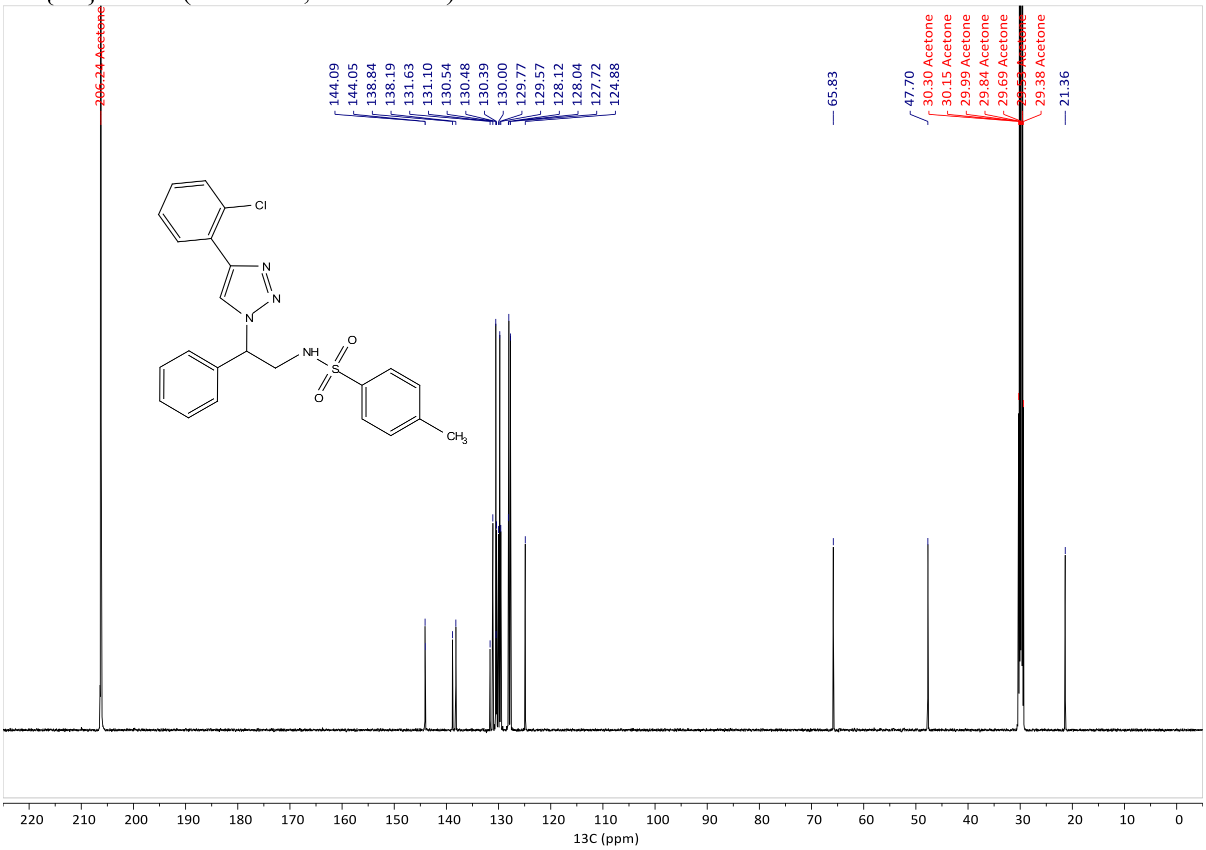

***N*-(2-(4-(3-Chlorophenyl)-1H-1,2,3-triazol-1-yl)-2-phenylethyl)-4-methylbenzenesulfonamide (13)**

^{1}H NMR (500 MHz, Acetone-d_6):

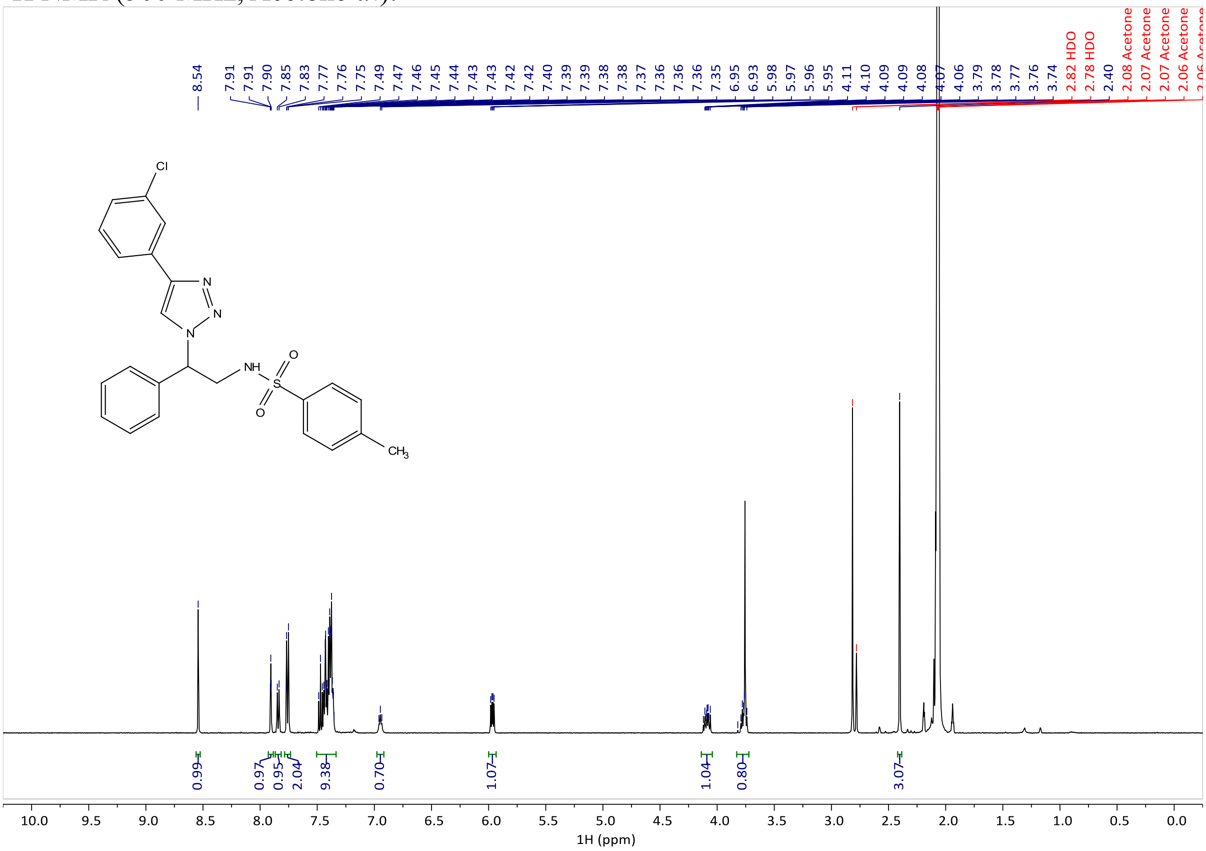

^{13}C{^{1}H} NMR (126 MHz, Acetone-d_6):

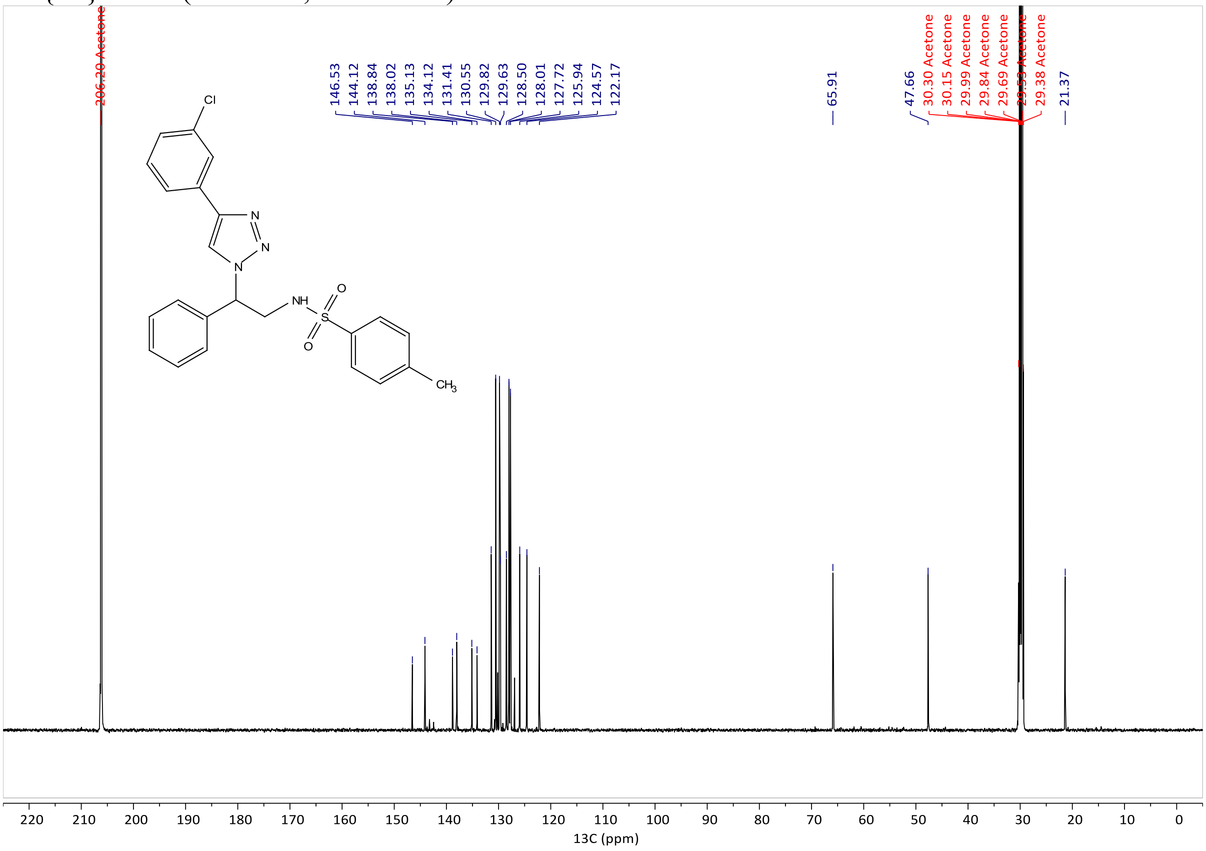

***N*-(2-(4-(4-Chlorophenyl)-1H-1,2,3-triazol-1-yl)-2-phenylethyl)-4-methylbenzenesulfonamide (14)**

^{1}H NMR (500 MHz, Acetone-*d*$_6$):

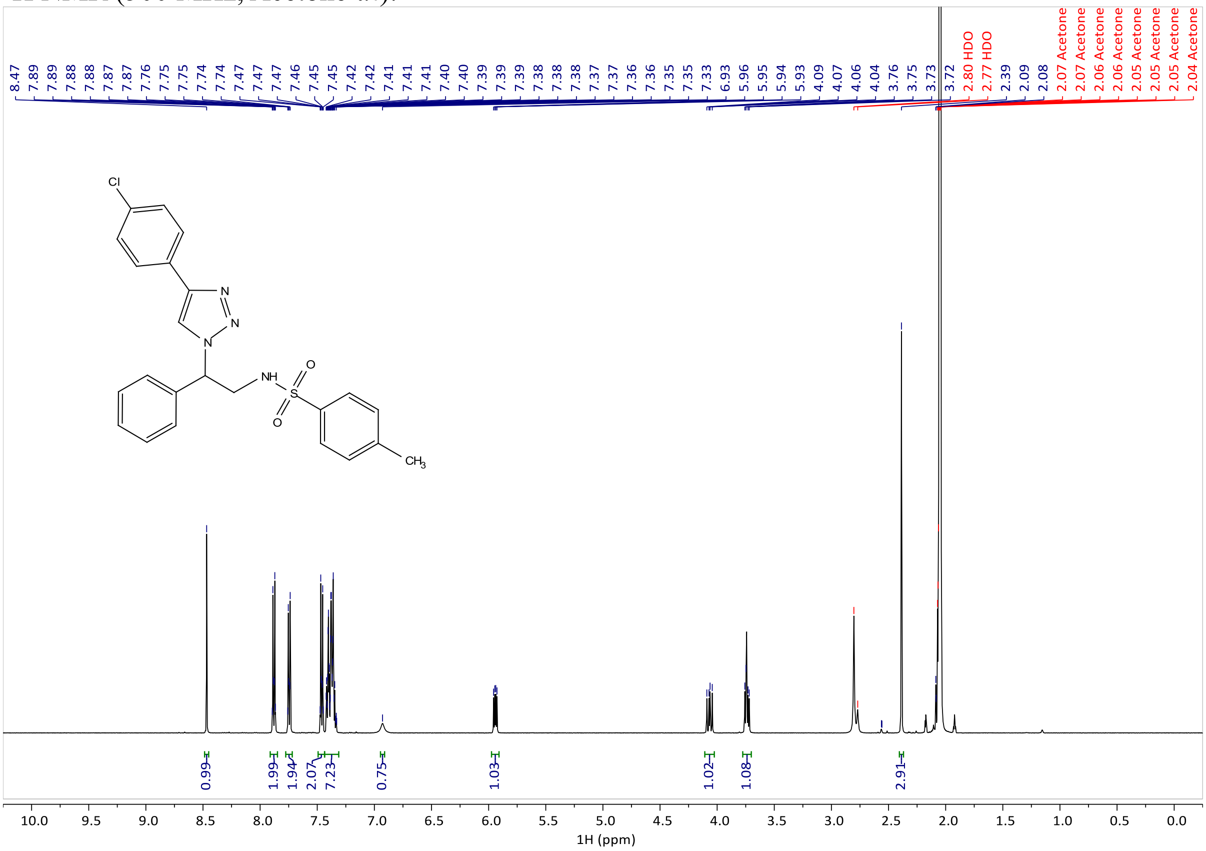

^{13}C{^{1}H} NMR (126 MHz, Acetone-*d*$_6$):

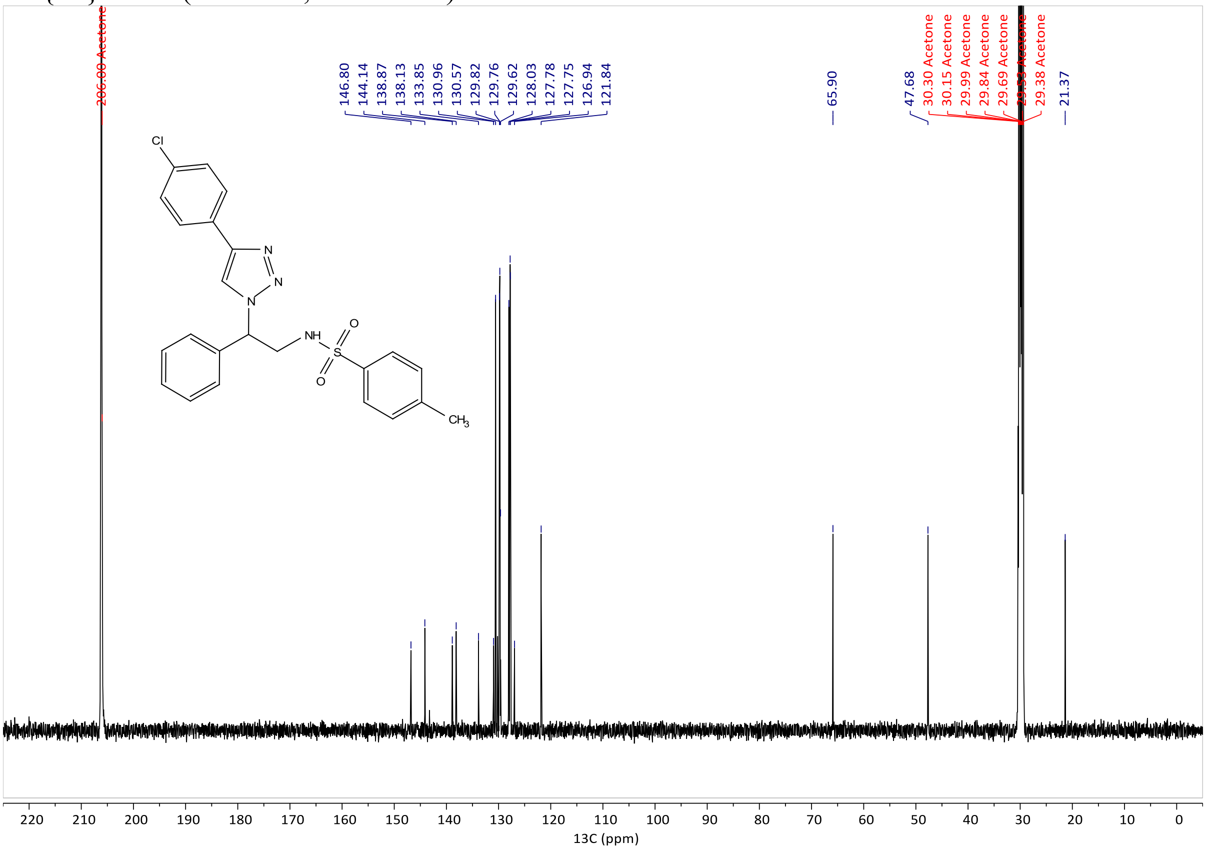

4-Methyl-*N*-(2-(4-(4-nitrophenyl)-1H-1,2,3-triazol-1-yl)-2-phenylethyl)benzenesulfonamide (15)

^{1}H NMR (500 MHz, Acetone-*d*$_6$):

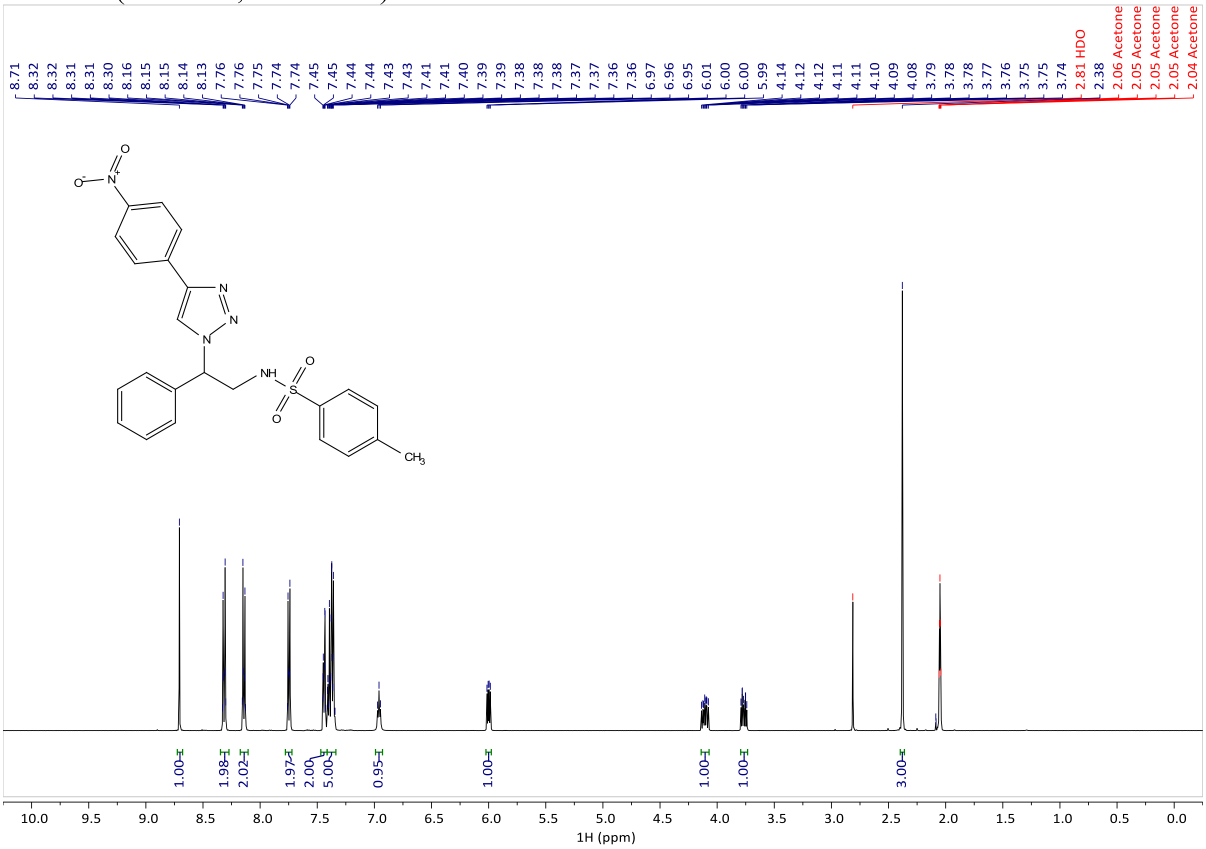

^{13}C{^{1}H} NMR (126 MHz, Acetone-*d*$_6$):

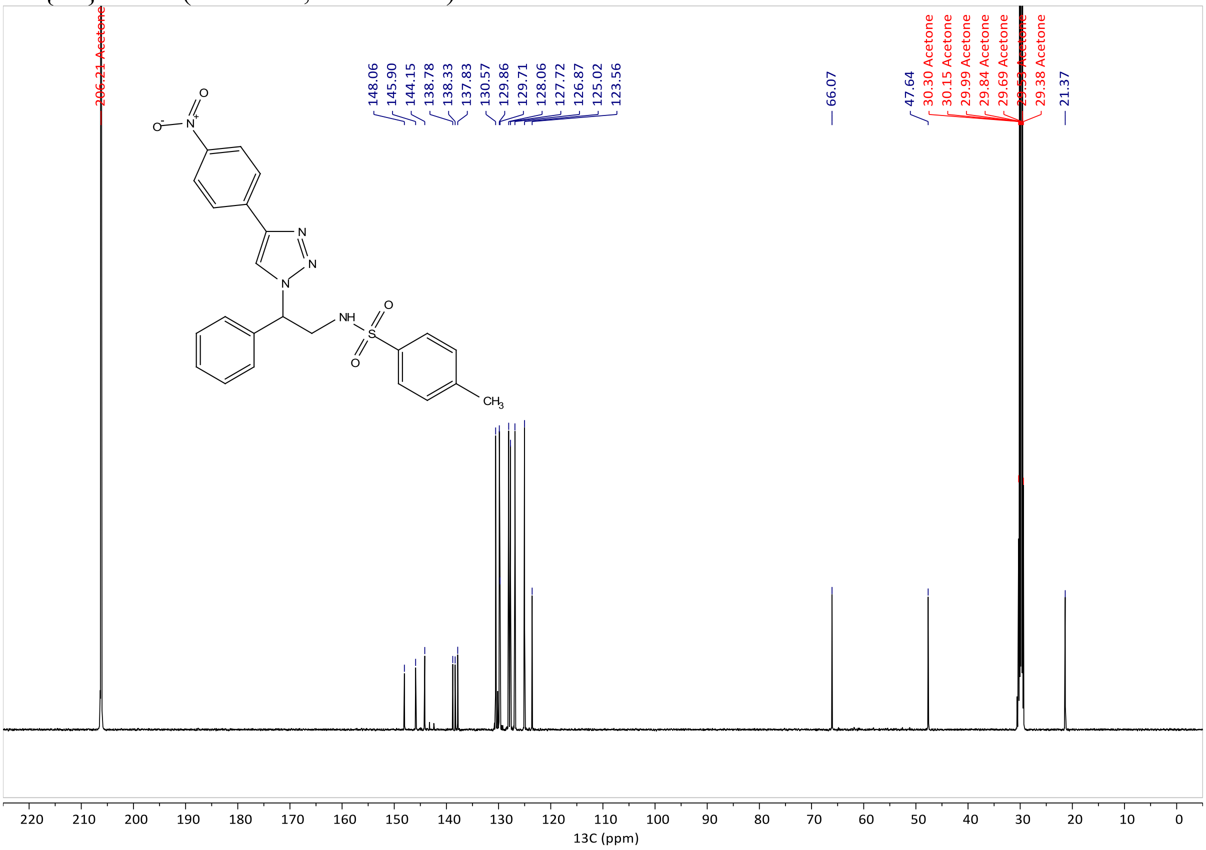

***N*-(2-(4-(4-Methoxyphenyl)-1H-1,2,3-triazol-1-yl)-2-phenylethyl)-4-methylbenzenesulfonamide (16)**

^{1}H NMR (500 MHz, Acetone-*d*$_6$):

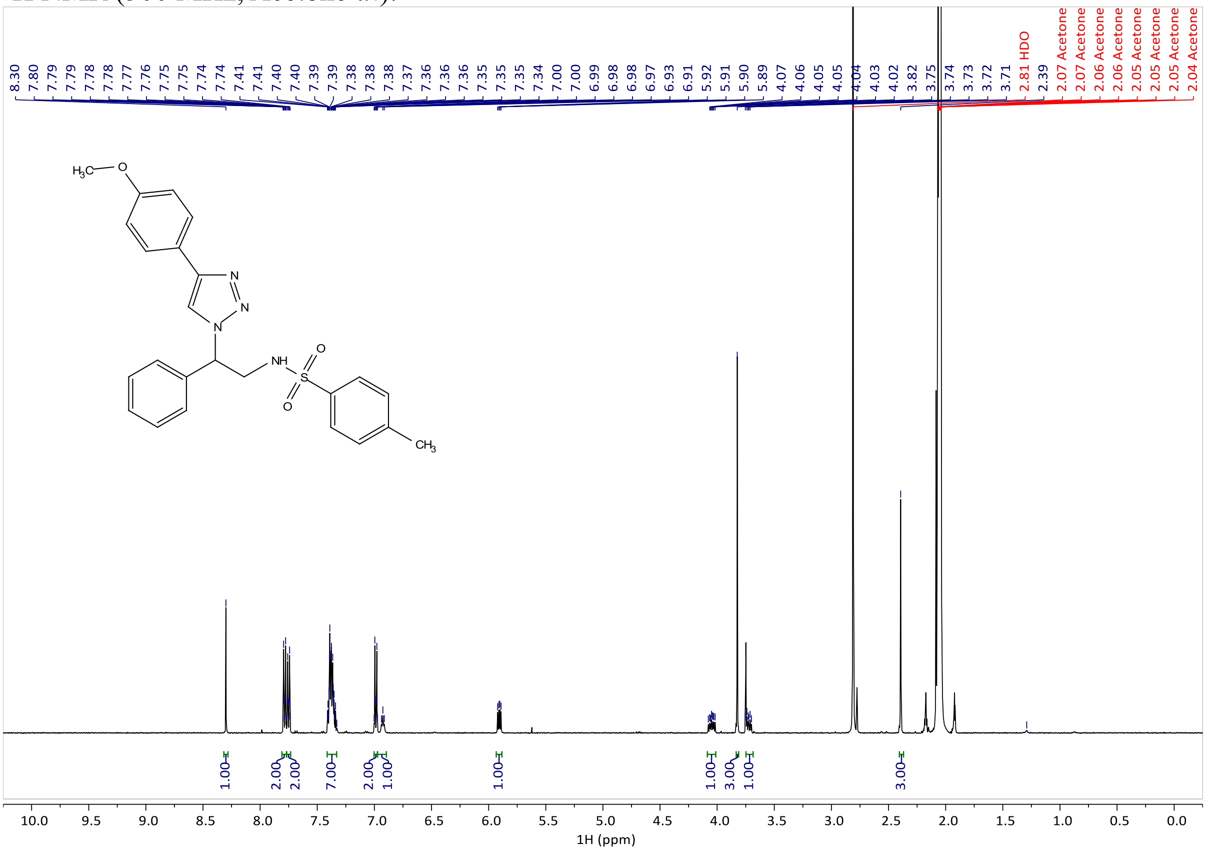

^{13}C{^{1}H} NMR (126 MHz, Acetone-*d*$_6$):

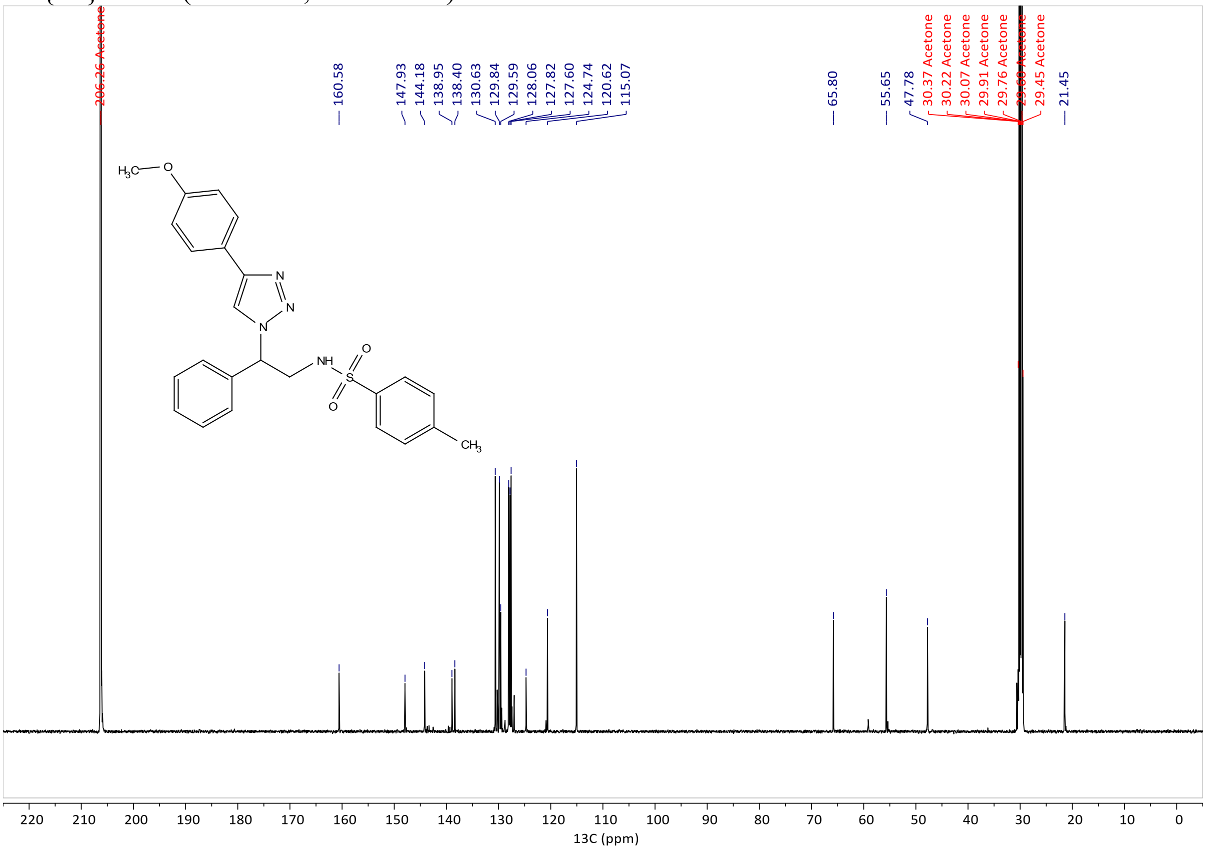

4-Methyl-*N*-(2-phenyl-2-(4-(pyridin-2-yl)-1H-1,2,3-triazol-1-yl)ethyl)benzenesulfonamide (17)

^{1}H NMR (500 MHz, Acetone-*d*$_6$):

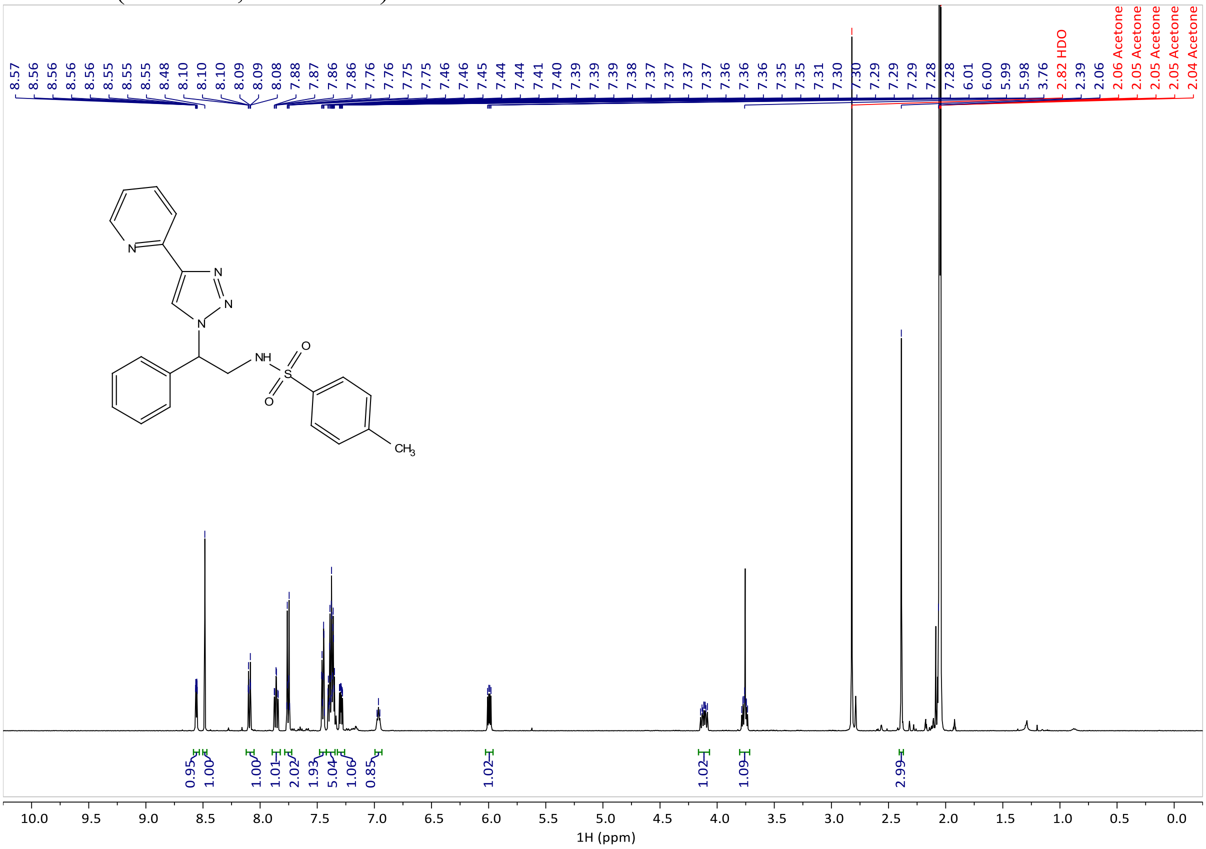

^{13}C{^{1}H} NMR (126 MHz, Acetone-*d*$_6$):

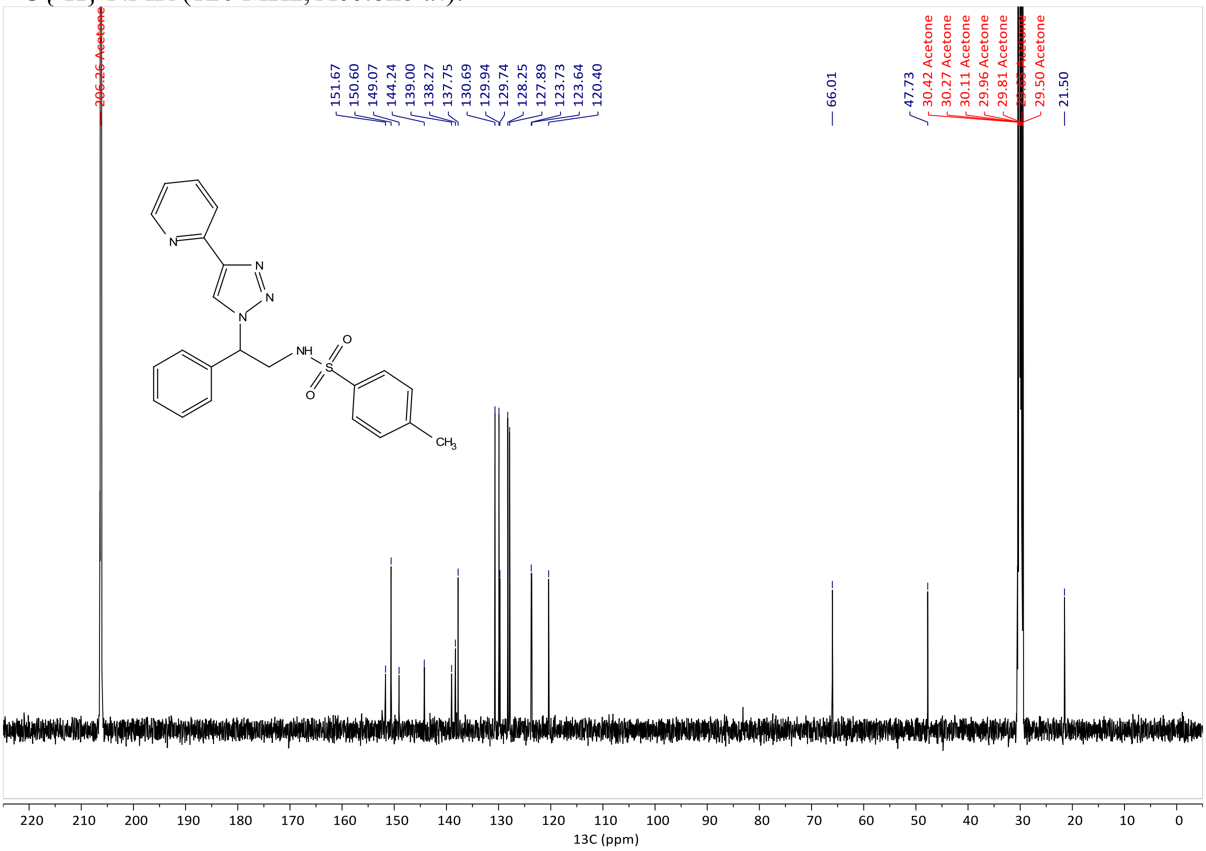

4-Methyl-*N*-(2-phenyl-2-(4-(trimethylsilyl)-1H-1,2,3-triazol-1-yl)ethyl)benzenesulfonamide (18)

^{1}H NMR (500 MHz, Acetone-*d*$_6$):

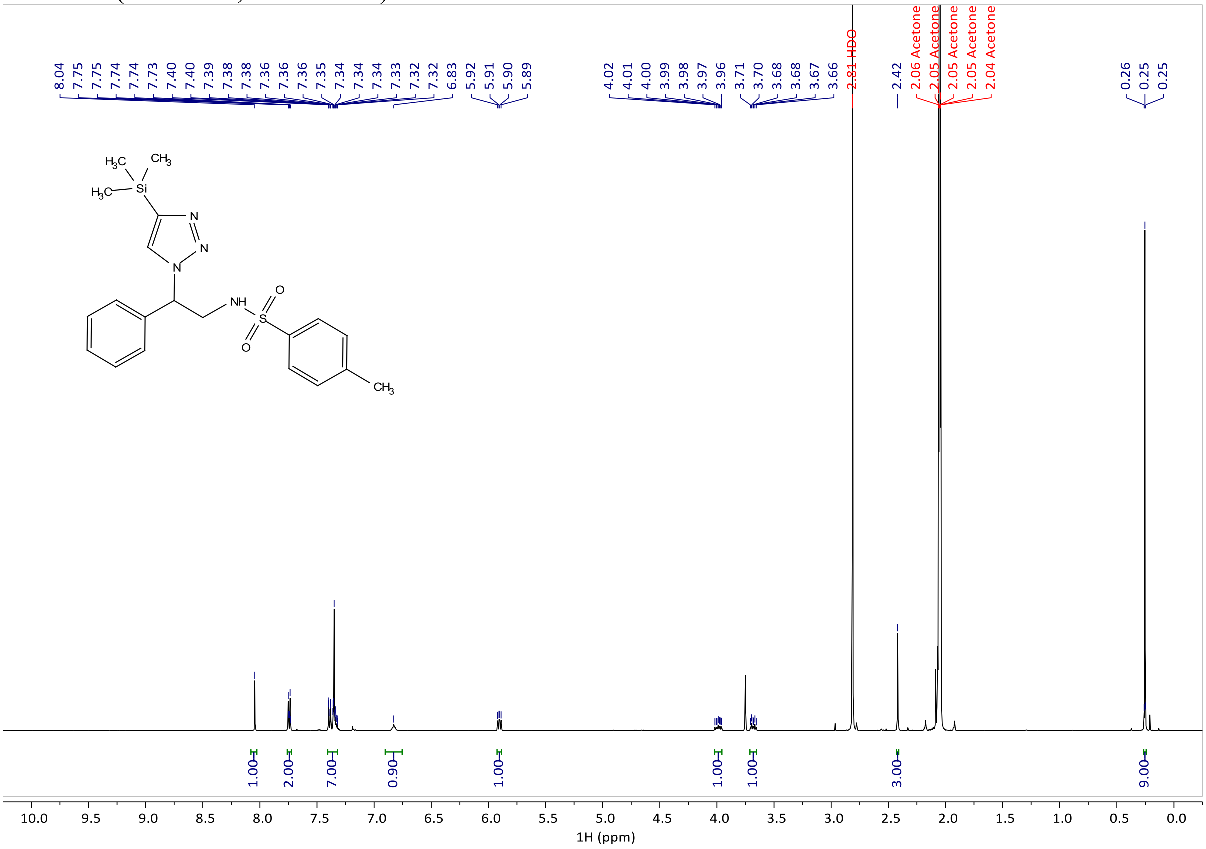

^{13}C{^{1}H} NMR (126 MHz, Acetone-*d*$_6$):

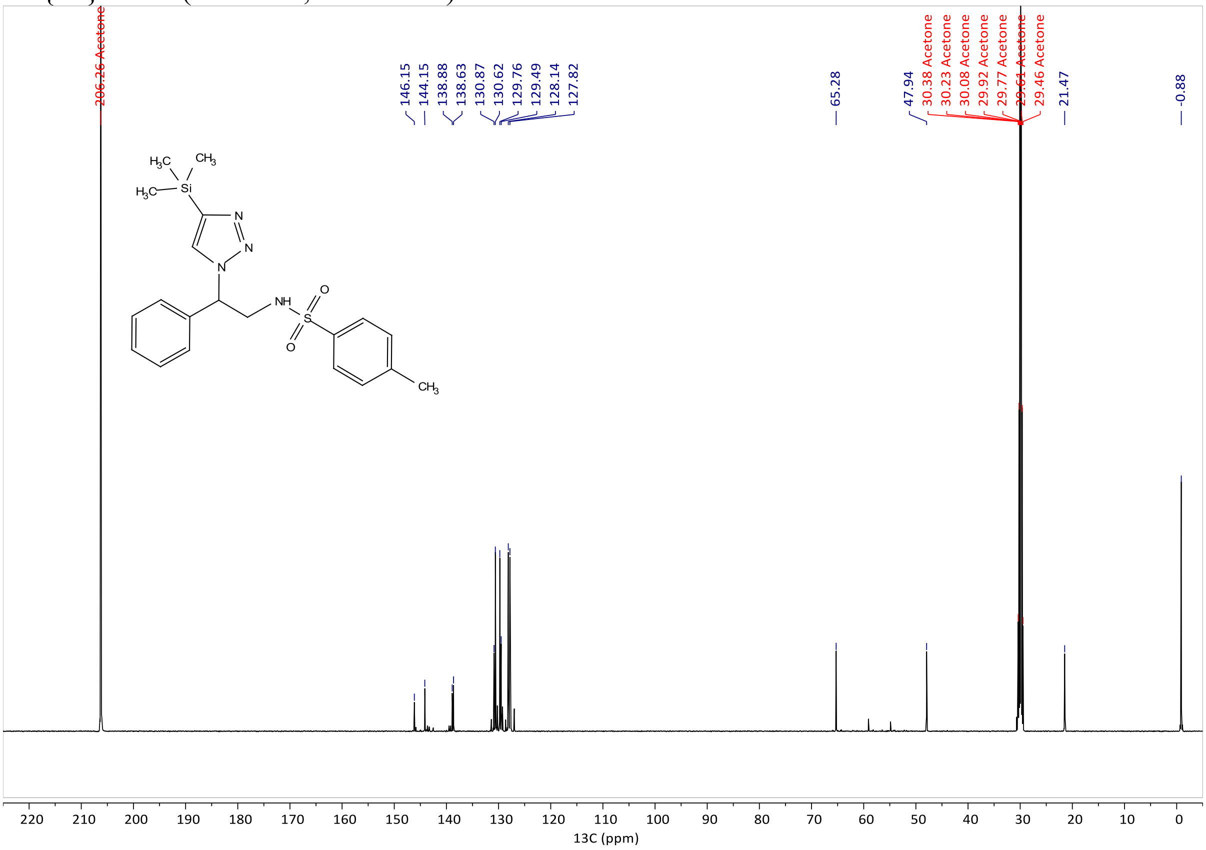

***N*-(2-(4-Butyl-1H-1,2,3-triazol-1-yl)-2-phenylethyl)-4-methylbenzenesulfonamide (19)**

^{1}H NMR (500 MHz, Acetone-*d*$_6$):

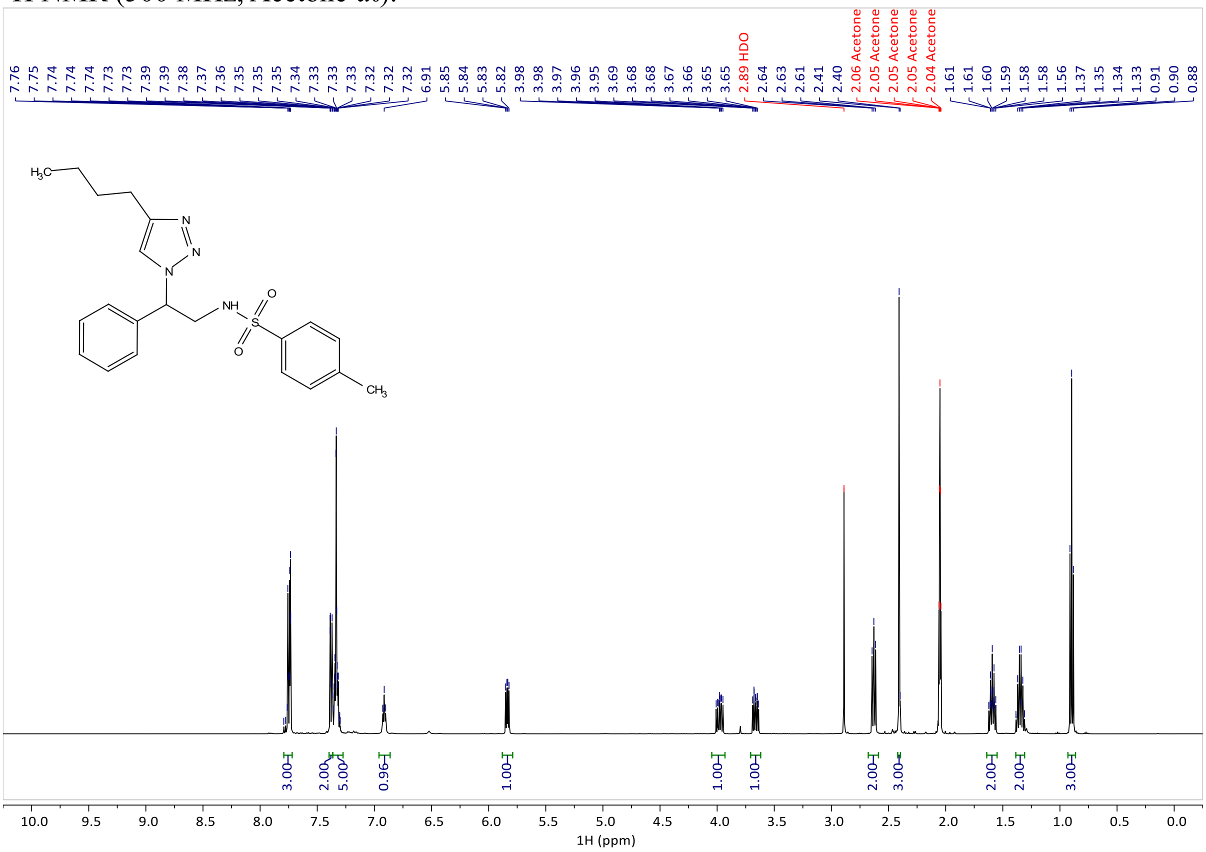

^{13}C{^{1}H} NMR (126 MHz, Acetone-*d*$_6$):

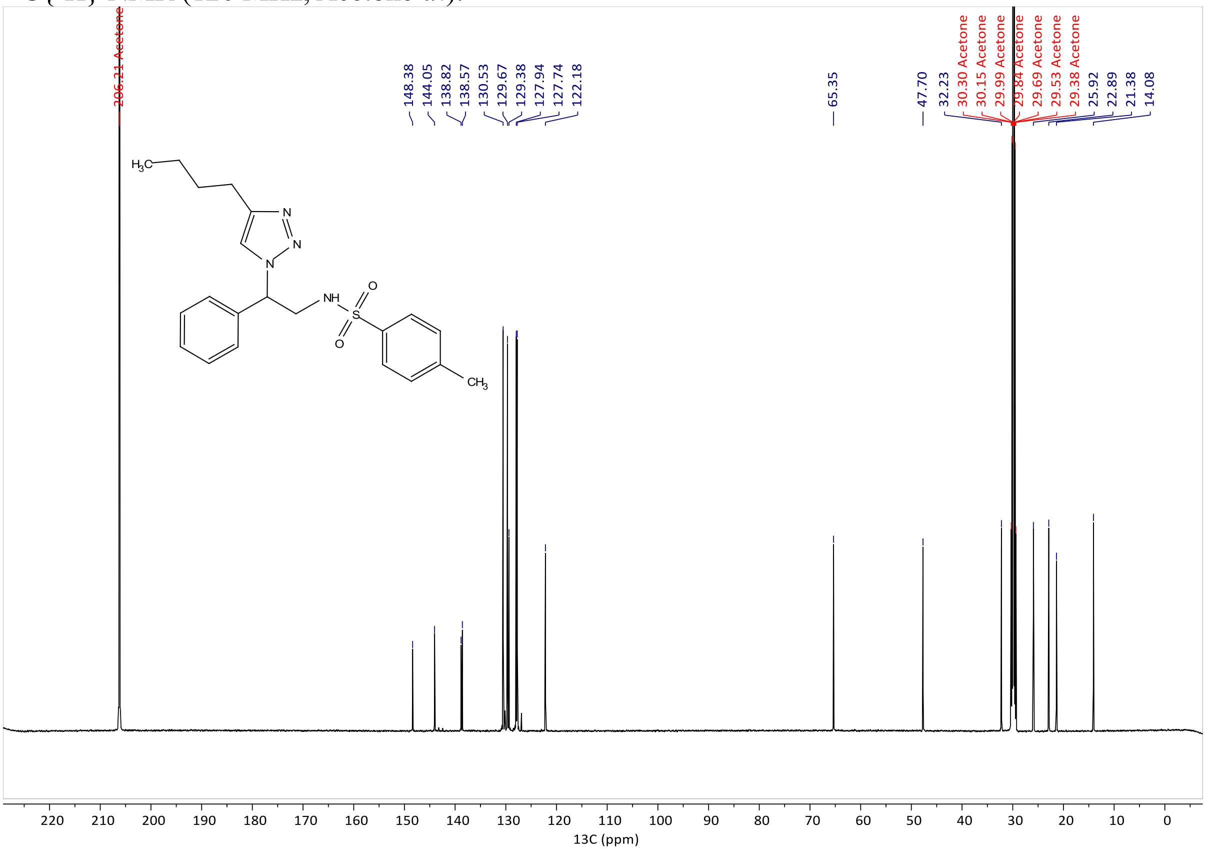

1-(2-((4-Methylphenyl)sulfonamido)-1-phenylethyl)-1H-1,2,3-triazole-4-carboxamide (20)

^{1}H NMR (500 MHz, DMSO-d_6):

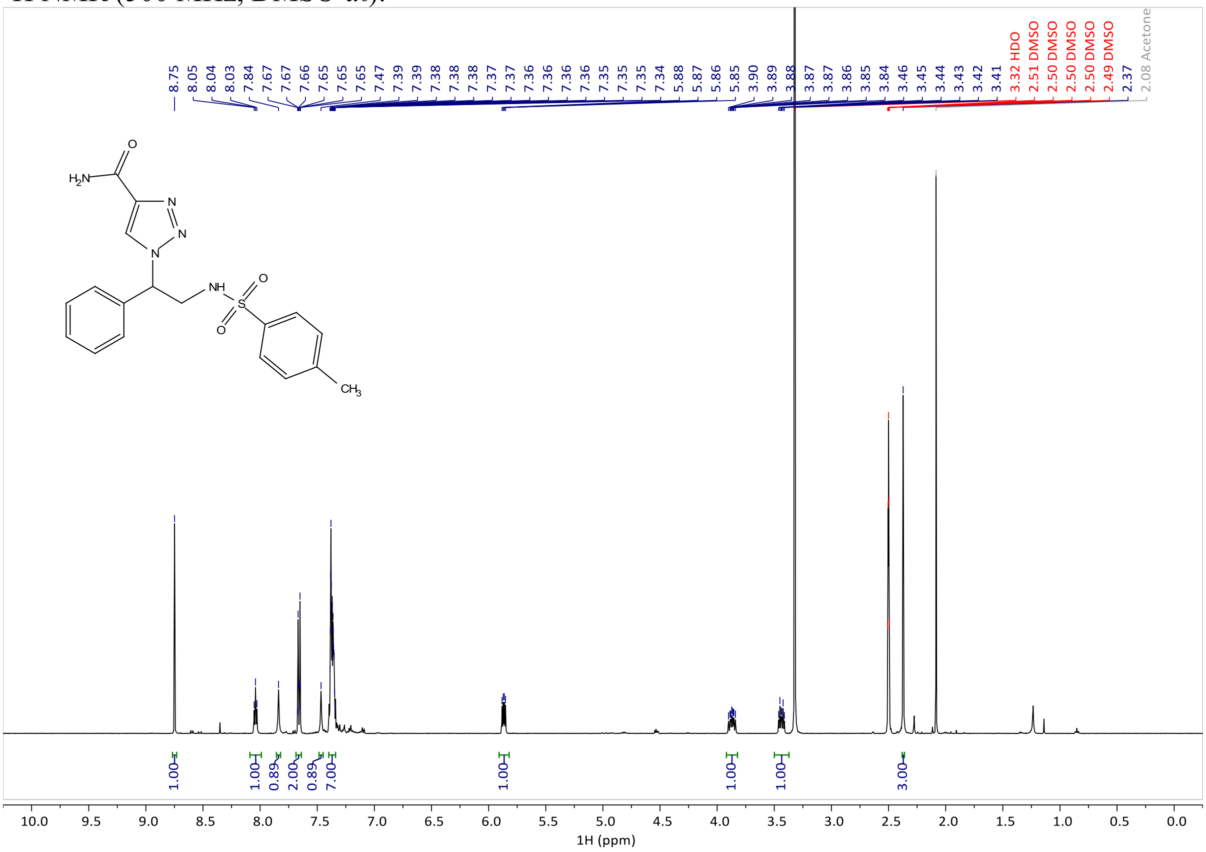

^{13}C{^{1}H} NMR (126 MHz, DMSO-d_6):

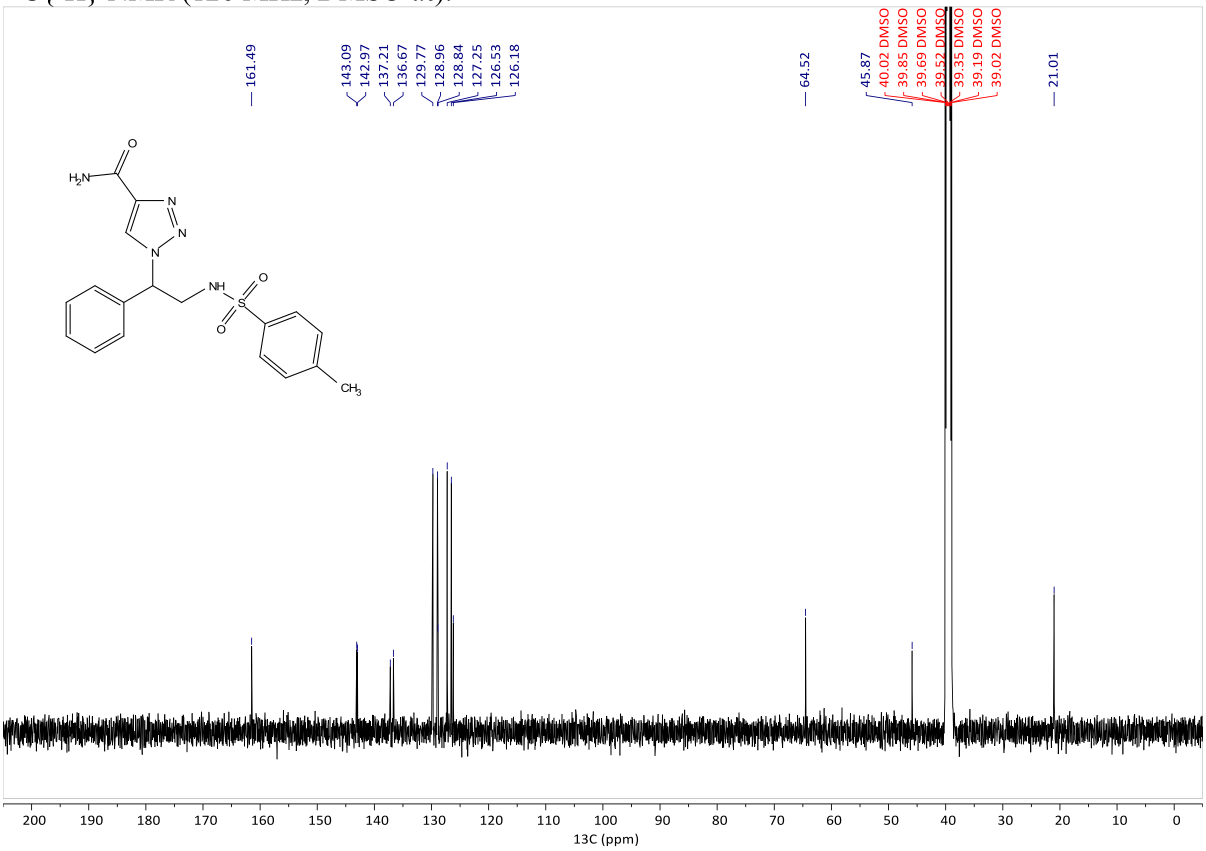

4-Nitro-*N*-(2-phenyl-2-(4-phenyl-1H-1,2,3-triazol-1-yl)ethyl)benzenesulfonamide (21)

^{1}H NMR (500 MHz, Acetone-*d*$_6$):

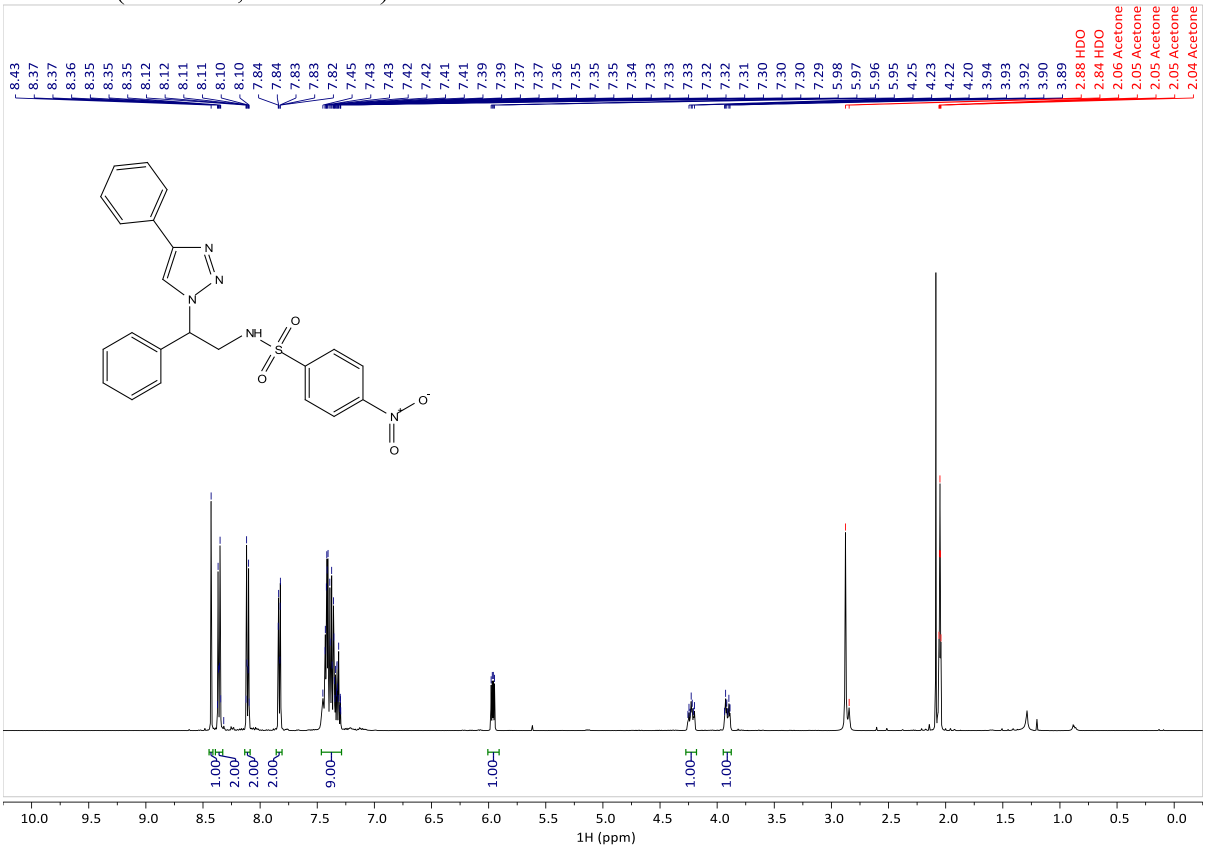

^{13}C{^{1}H} NMR (126 MHz, Acetone-*d*$_6$):

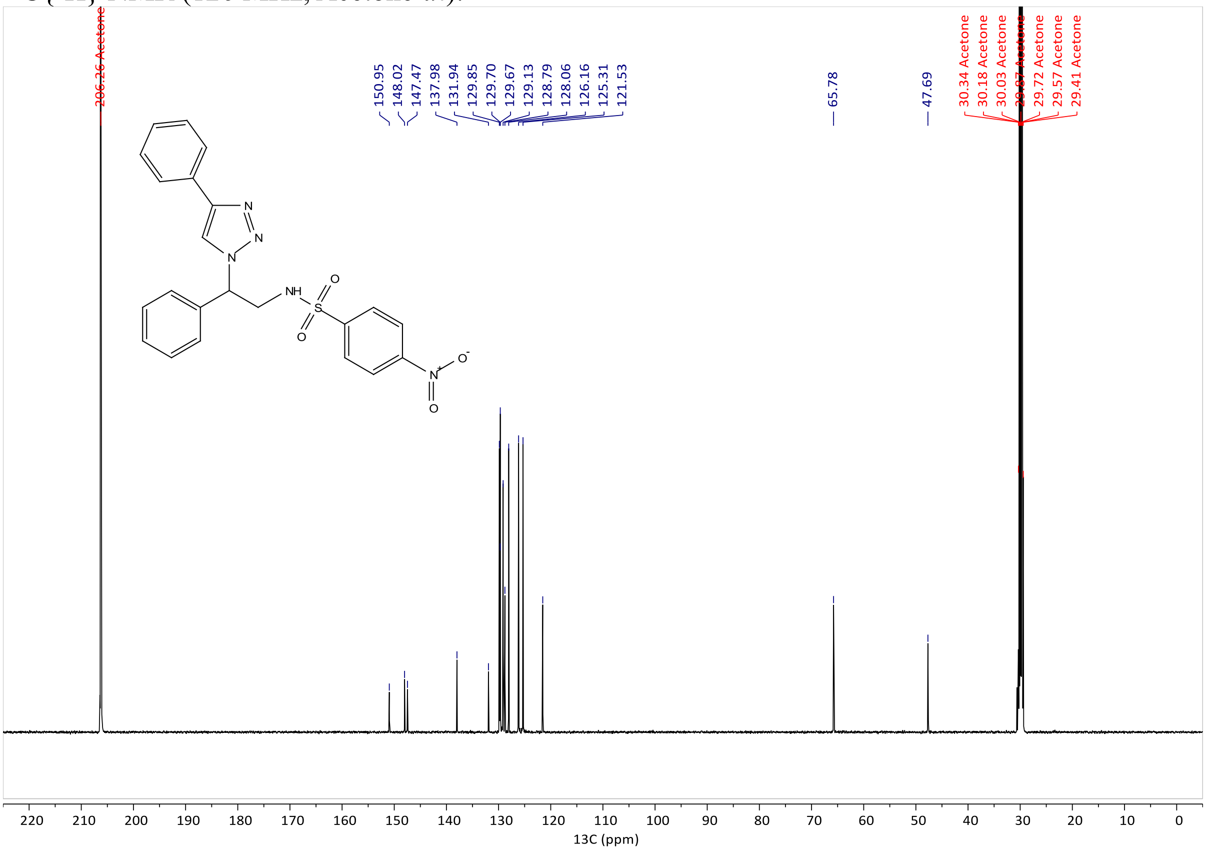

***N*-(2-Phenyl-2-(4-phenyl-1H-1,2,3-triazol-1-yl)ethyl)-4-(trifluoromethyl)benzenesulfonamide (22)**

^{1}H NMR (500 MHz, Acetone-*d*6):

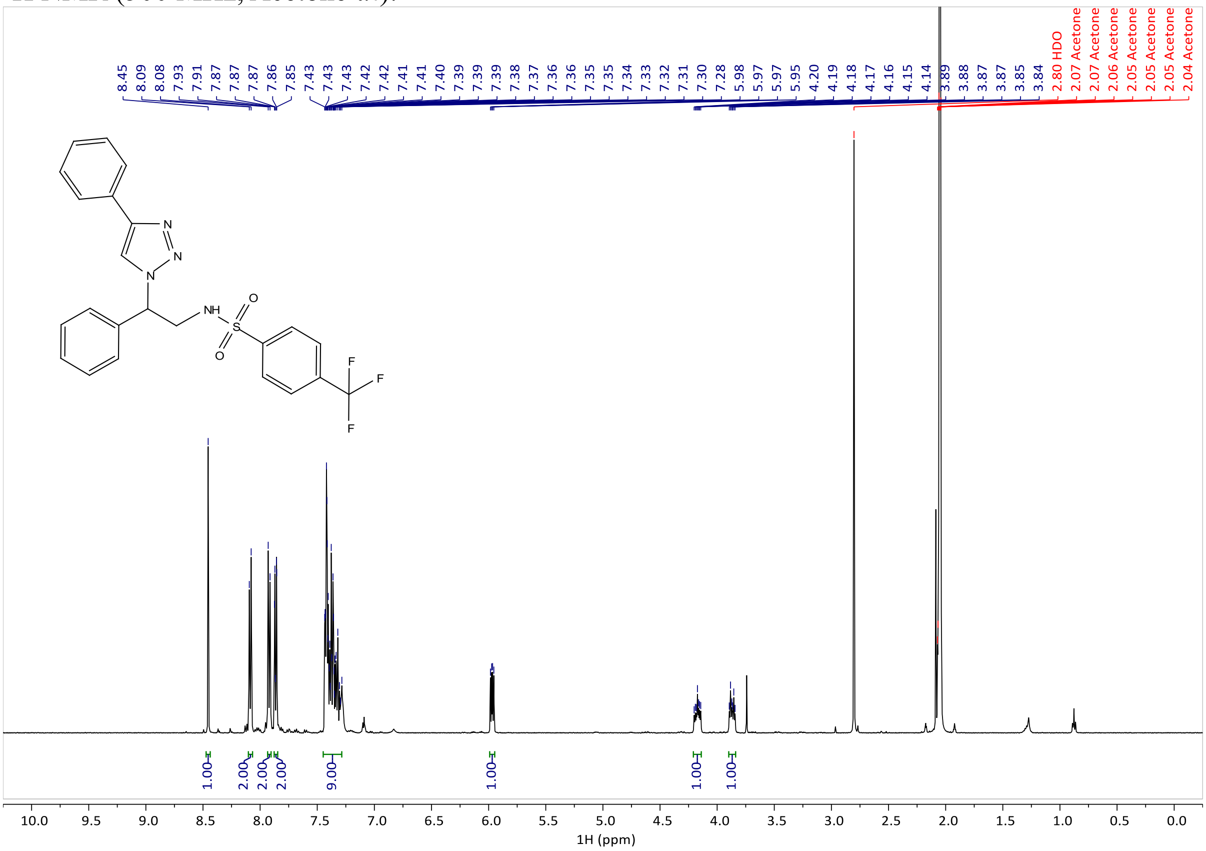

^{13}C{^{1}H} NMR (126 MHz, Acetone-*d*6):

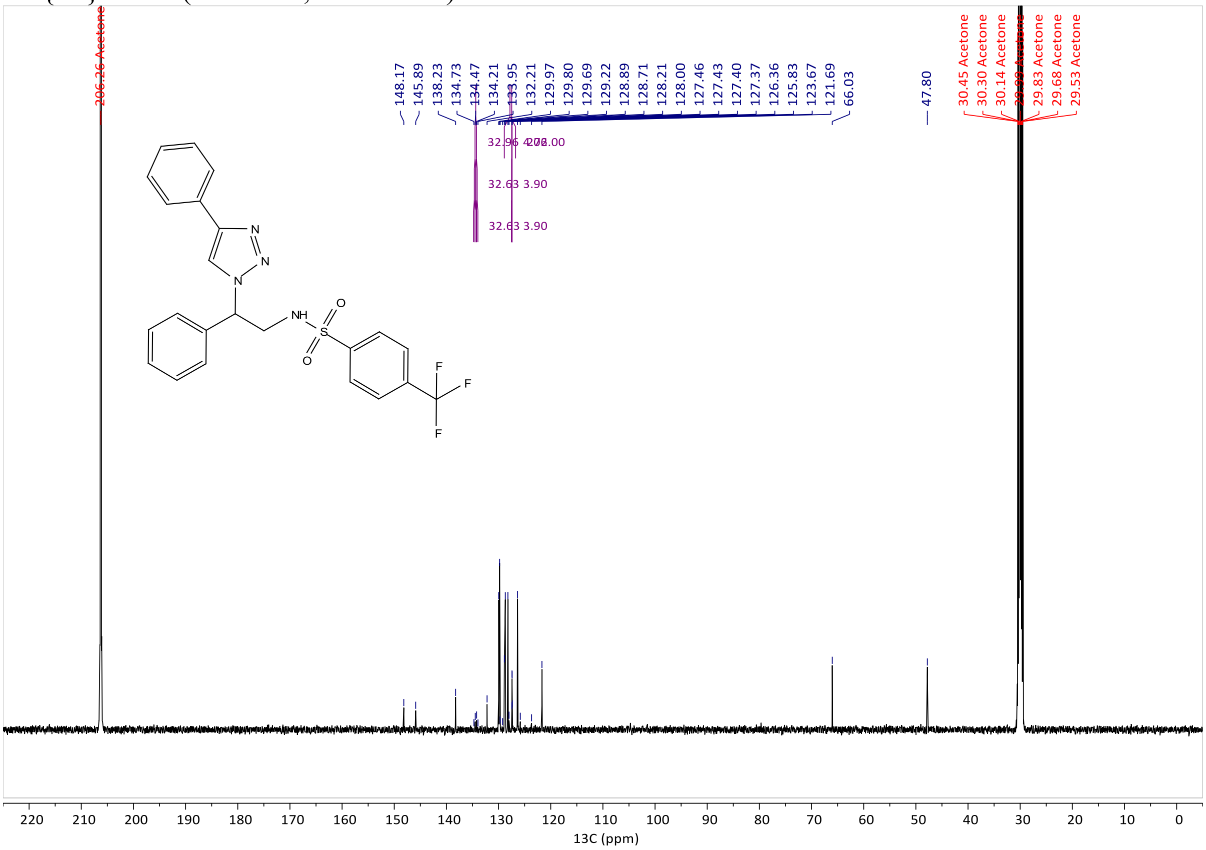

^{19}F NMR (470 MHz, Acetone-d_6):

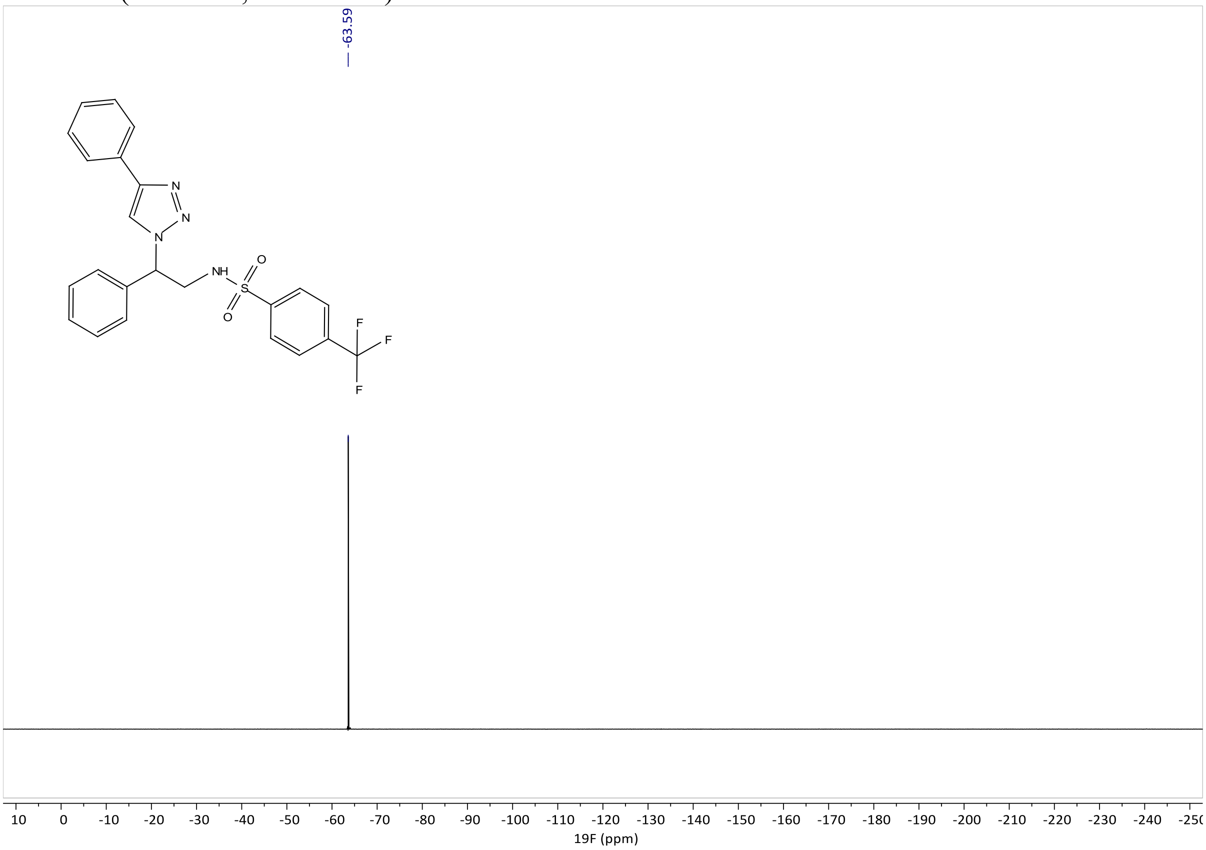

2,6-Difluoro-*N*-(2-phenyl-2-(4-phenyl-1*H*-1,2,3-triazol-1-yl)ethyl)benzenesulfonamide (23)

^{1}H NMR (500 MHz, DMSO-d_6):

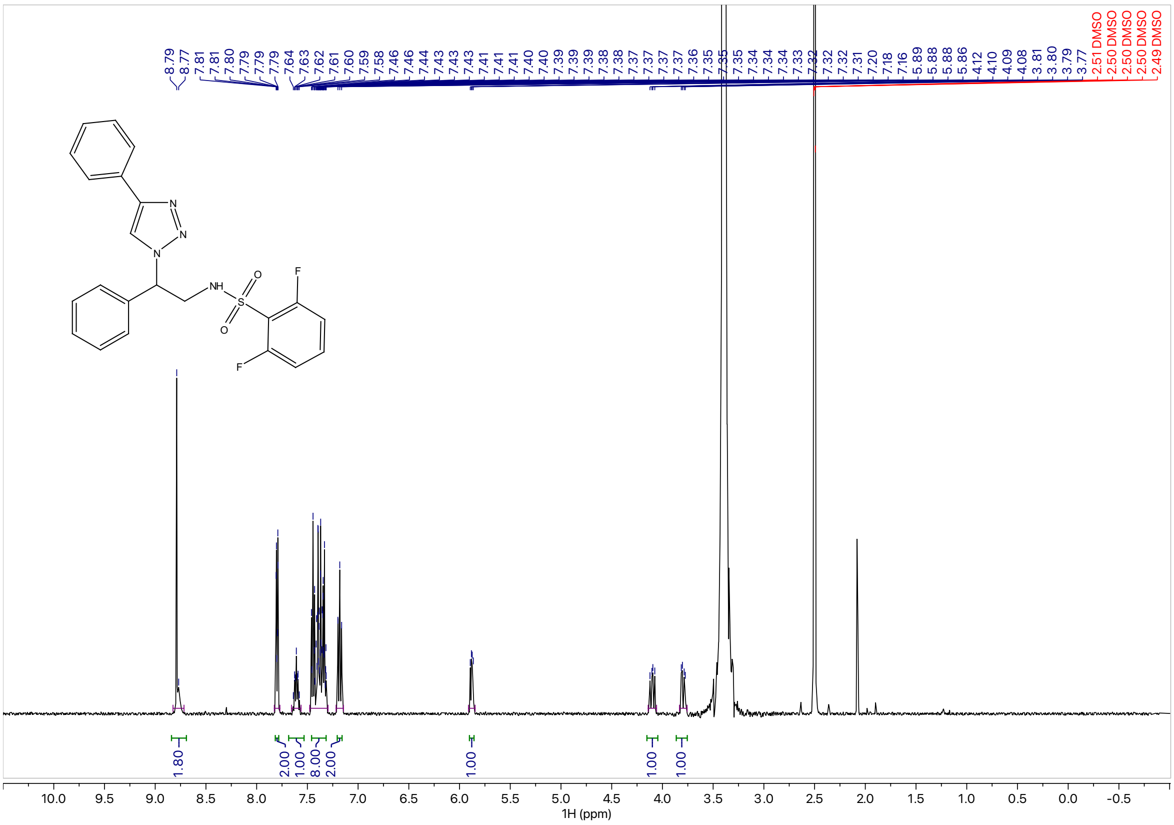

^{13}C{^{1}H} NMR (126 MHz, DMSO-d_6):

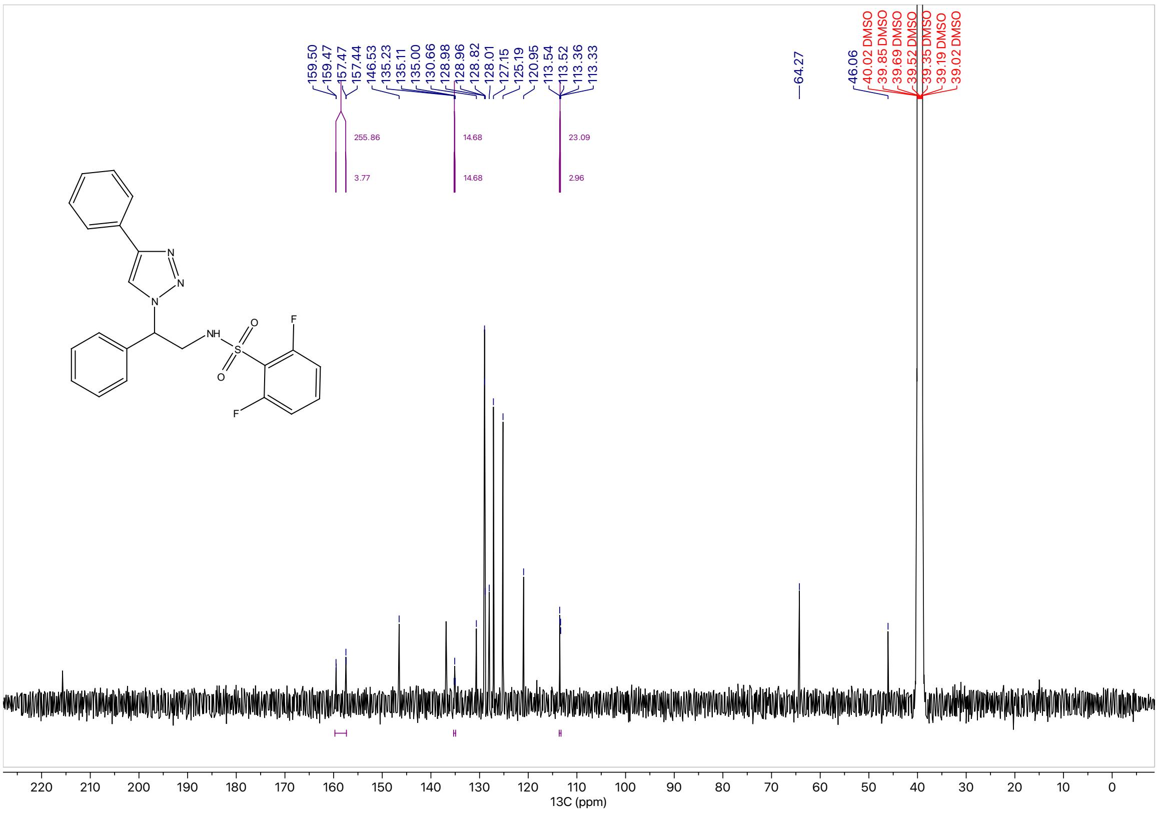

^{19}F NMR (470 MHz, DMSO-d_6):

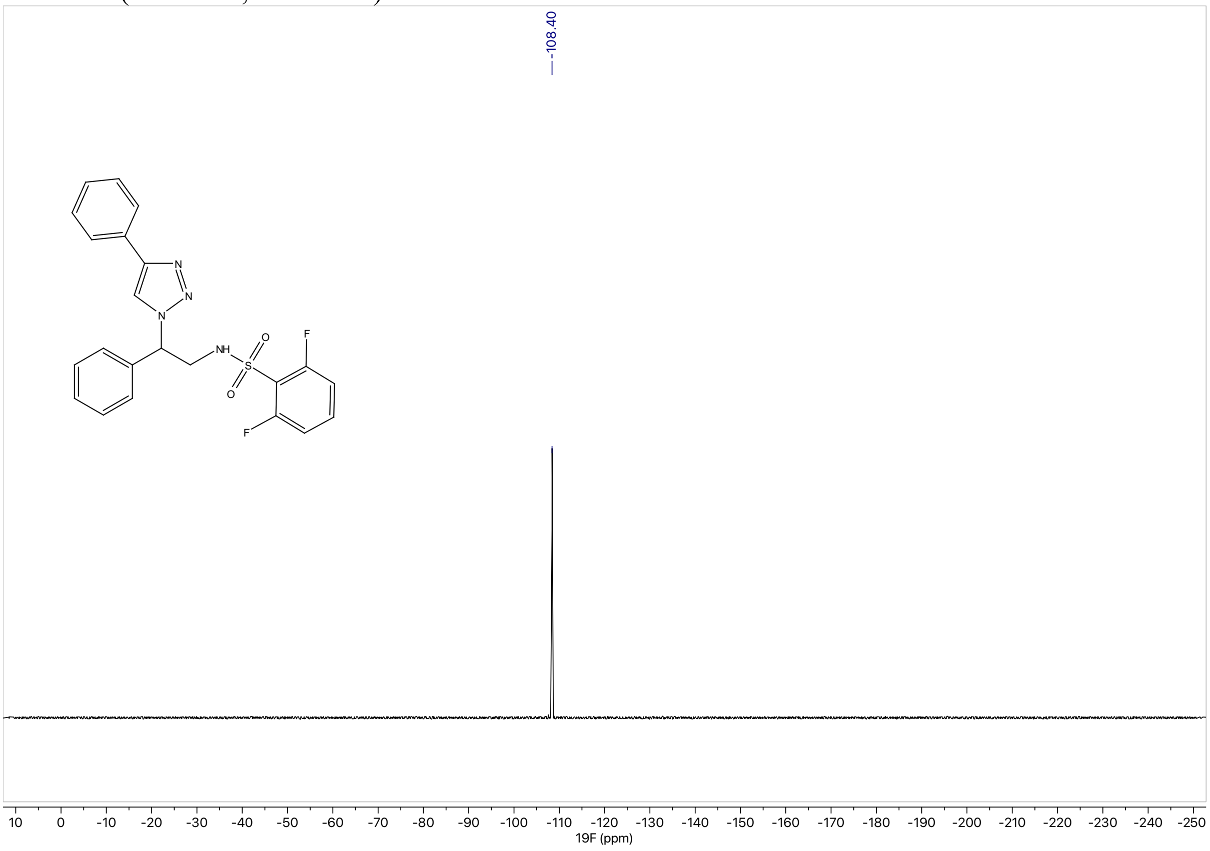

_N_-(2-(Dibenzo[_b_,_d_]thiophen-2-yl)-2-(4-phenyl-1H-1,2,3-triazol-1-yl)ethyl)-4-methylbenzenesulfonamide (24)

^{1}H NMR (500 MHz, DMSO-d_6):

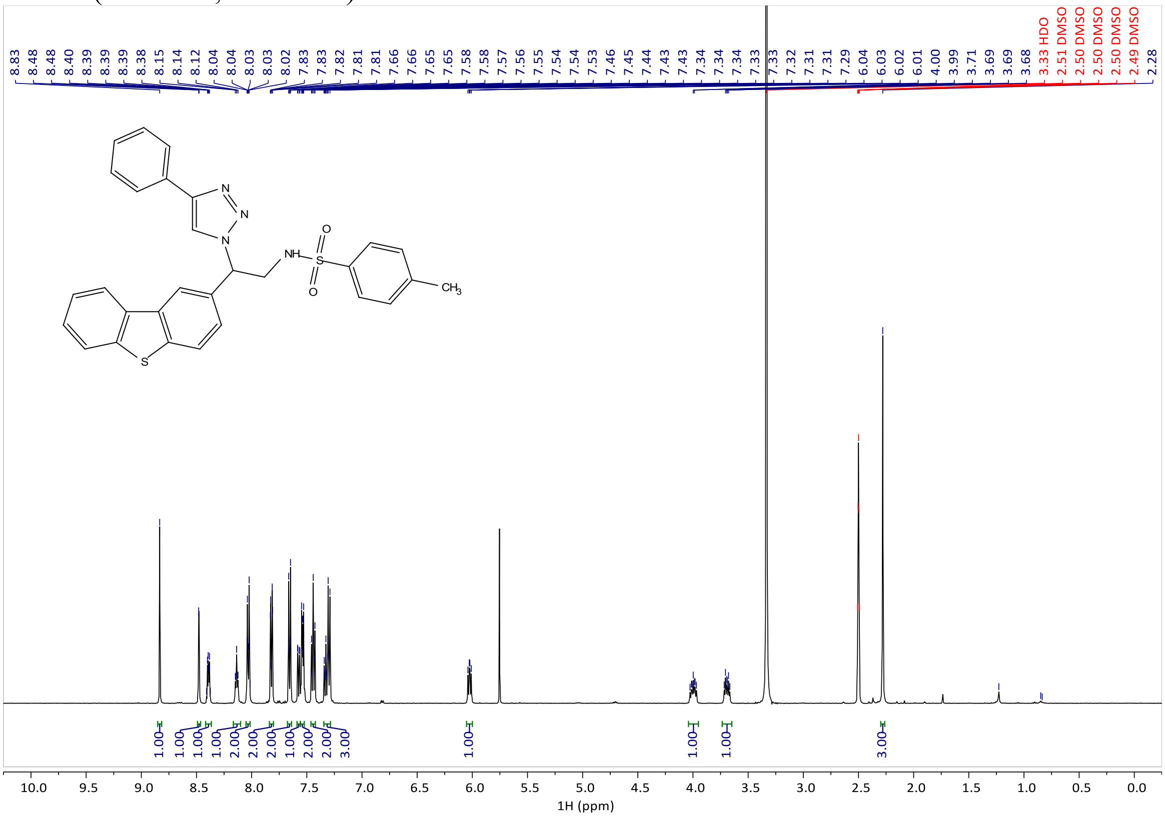

^{13}C{^{1}H} NMR (126 MHz, DMSO-d_6):

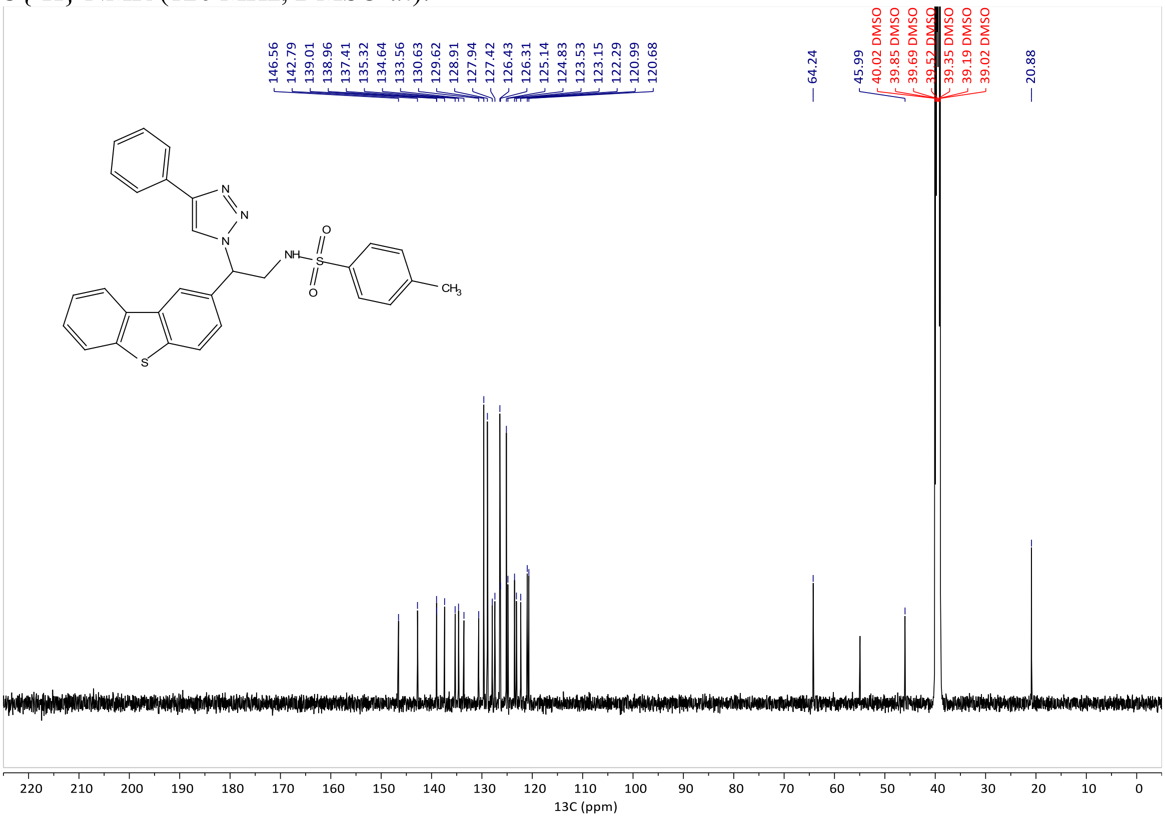

***N*-(2-(2,6-difluorophenyl)-2-(4-(trimethylsilyl)-1H-1,2,3-triazol-1-yl)ethyl)-4-nitrobenzenesulfonamide (25)**

^{1}H NMR (500 MHz, Acetone-d_6):

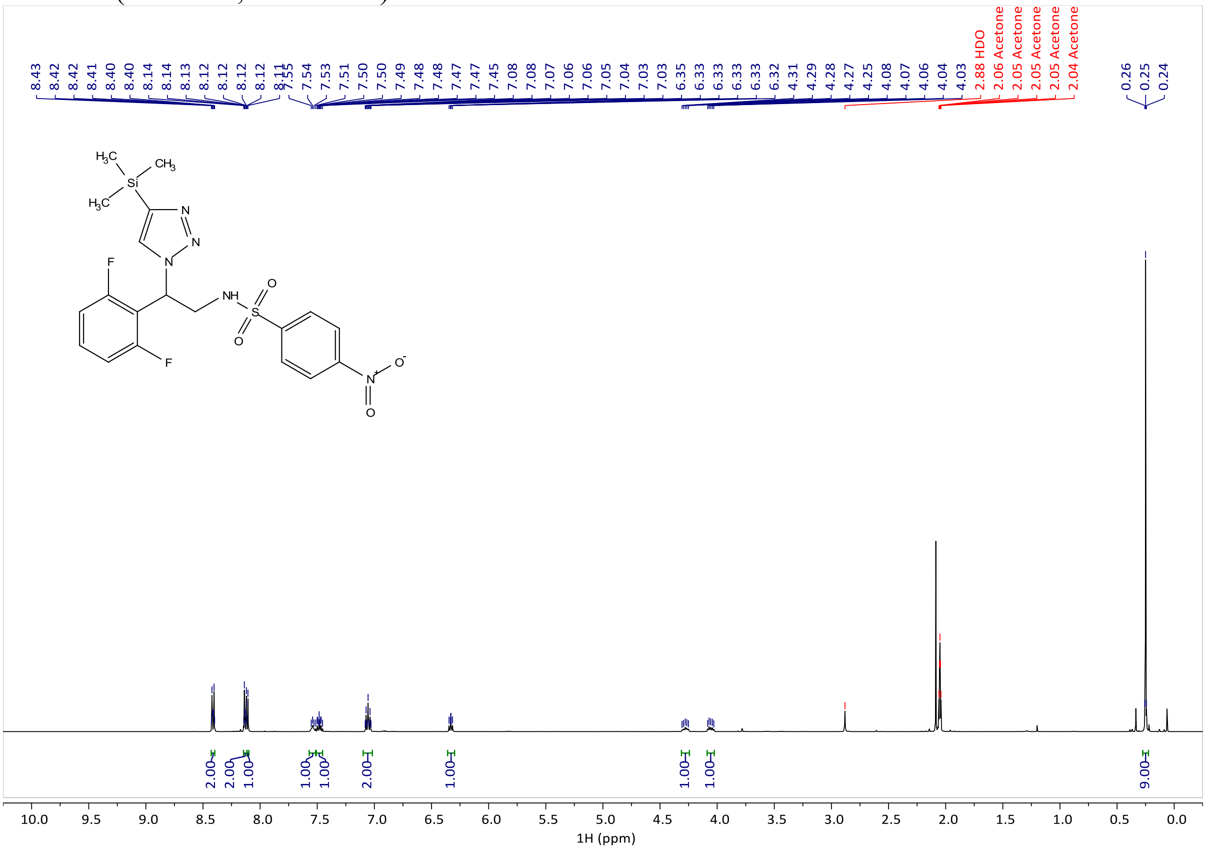

$^{13}C\{^{1}H\}$ NMR (126 MHz, Acetone-d_6):

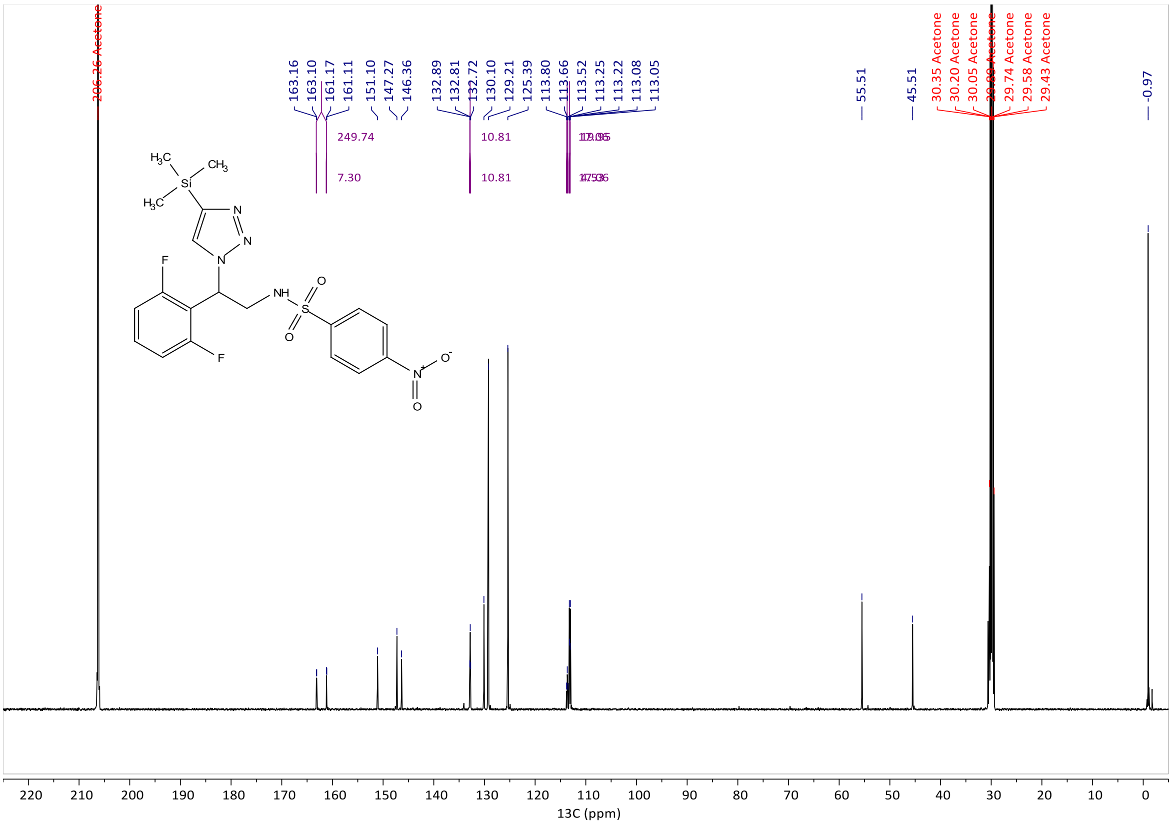

^{19}F NMR (377 MHz, Acetone-d_6):

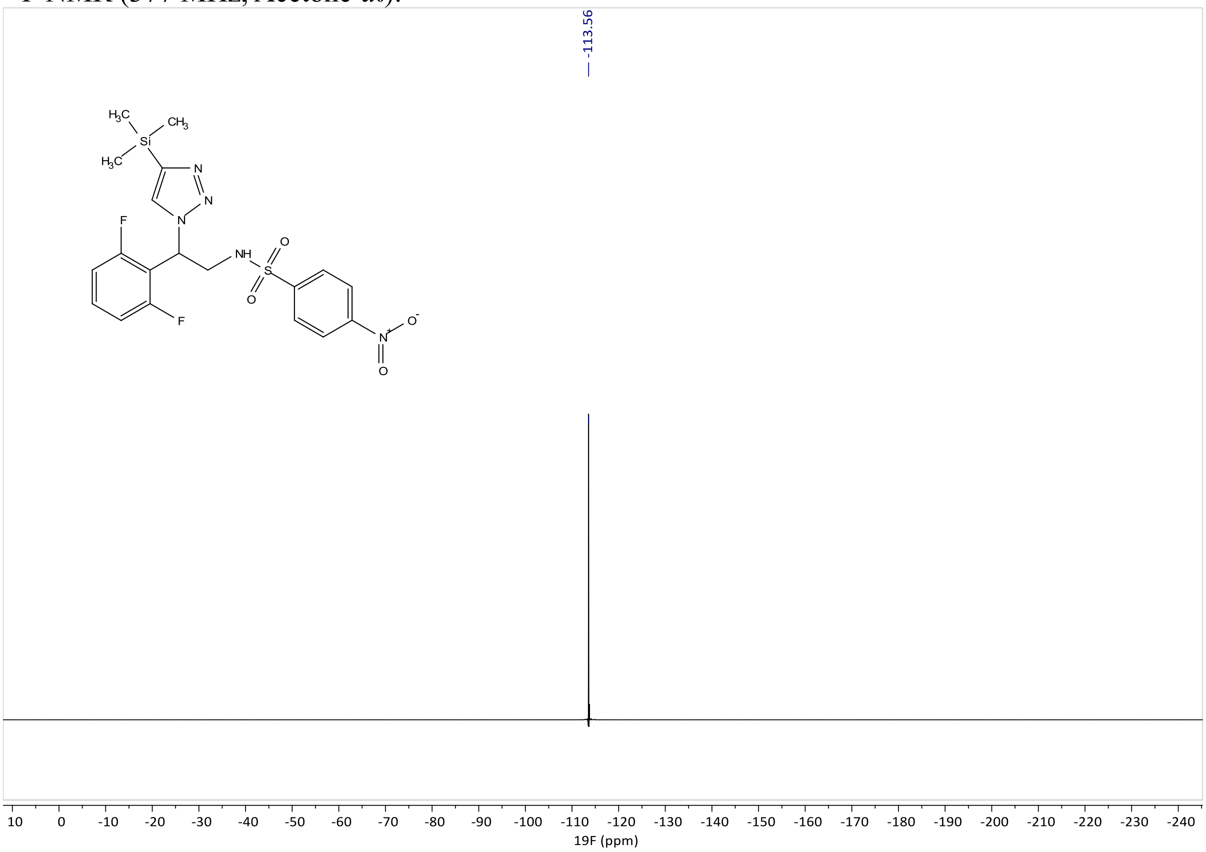

***N*-(2-(4-(4-methoxyphenyl)-1H-1,2,3-triazol-1-yl)-2-(3,4,5-trimethoxyphenyl)ethyl)-4-nitrobenzenesulfonamide (26)**

^{1}H NMR (500 MHz, Acetone-d_6):

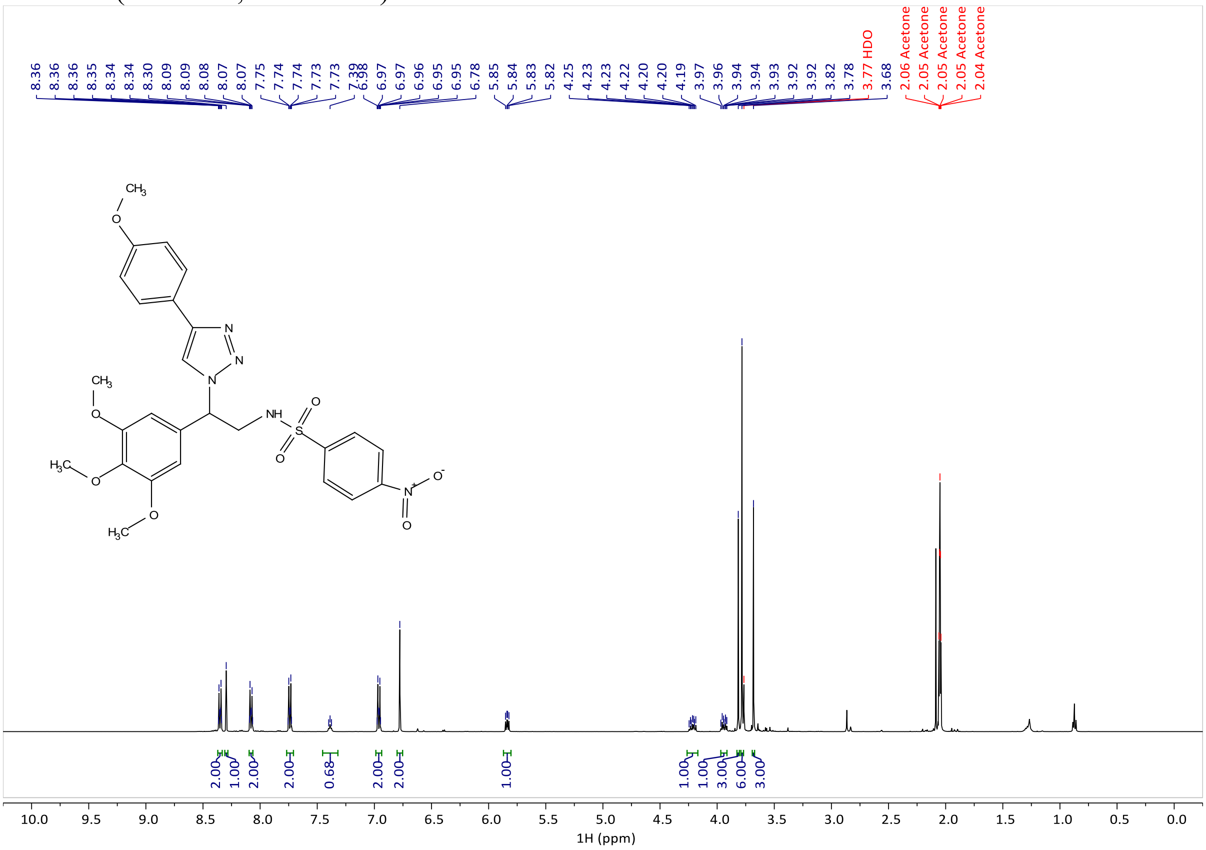

^{13}C{^{1}H} NMR (126 MHz, Acetone-d_6):

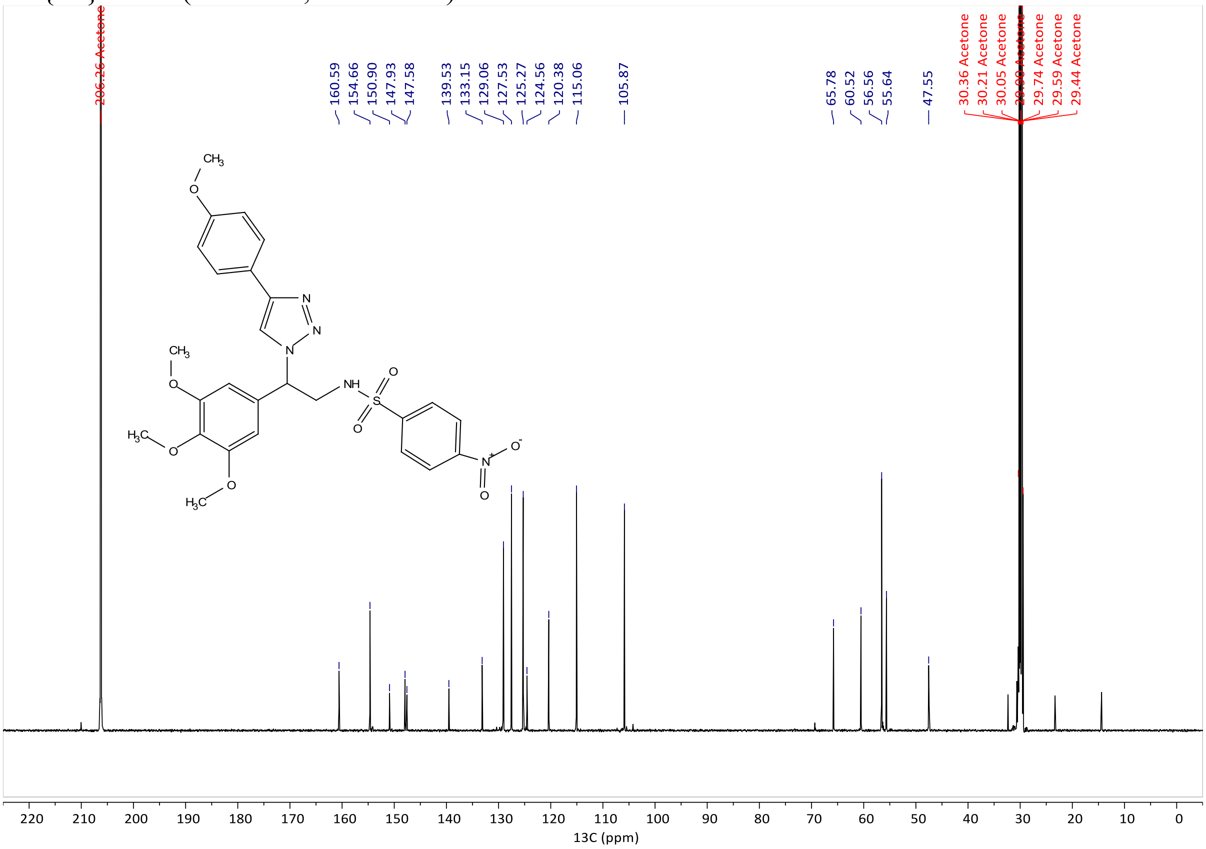

Methyl 4-(1-(4-carbamoyl-1H-1,2,3-triazol-1-yl)-2-((4-methylphenyl)sulfonamido)ethyl)-2,6-dichlorobenzoate (27)

^{1}H NMR (500 MHz, Acetone-*d*$_6$):

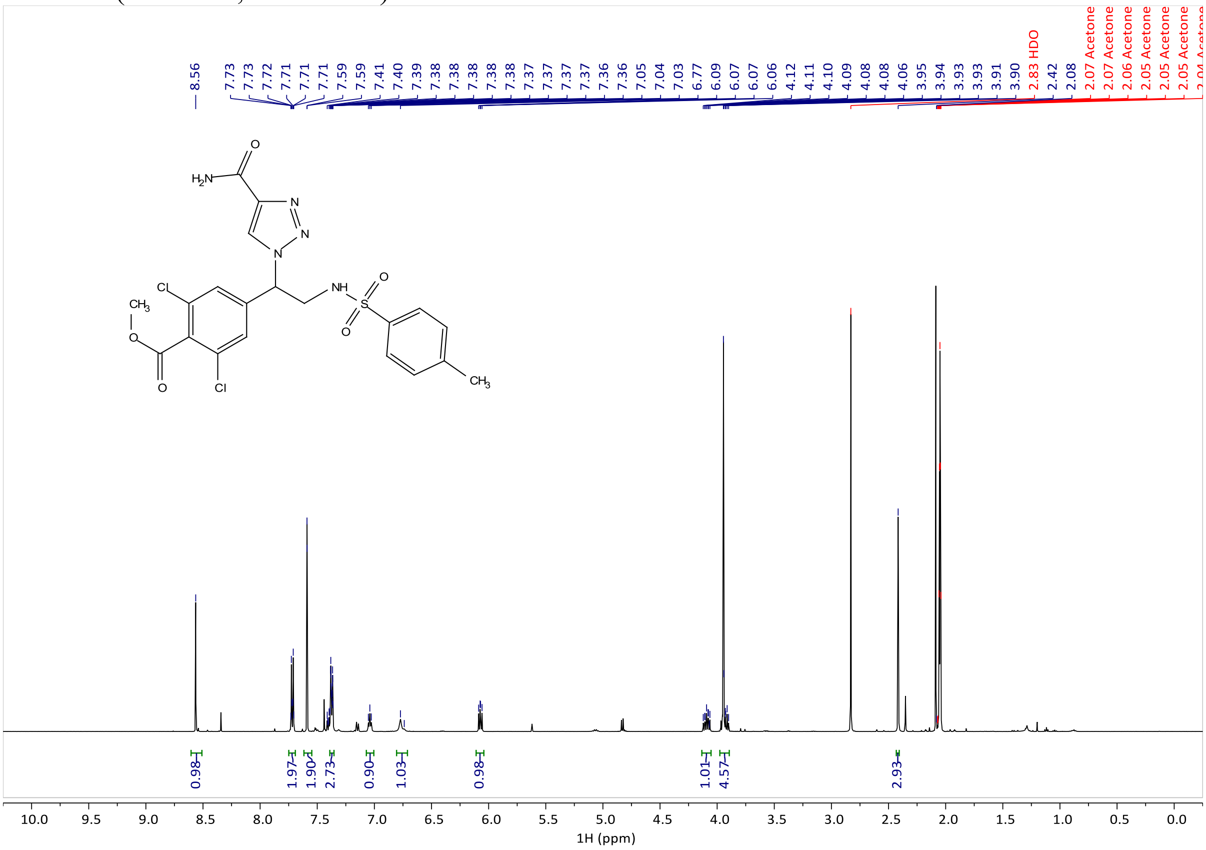

^{13}C {^{1}H} NMR (126 MHz, Acetone-*d*$_6$):

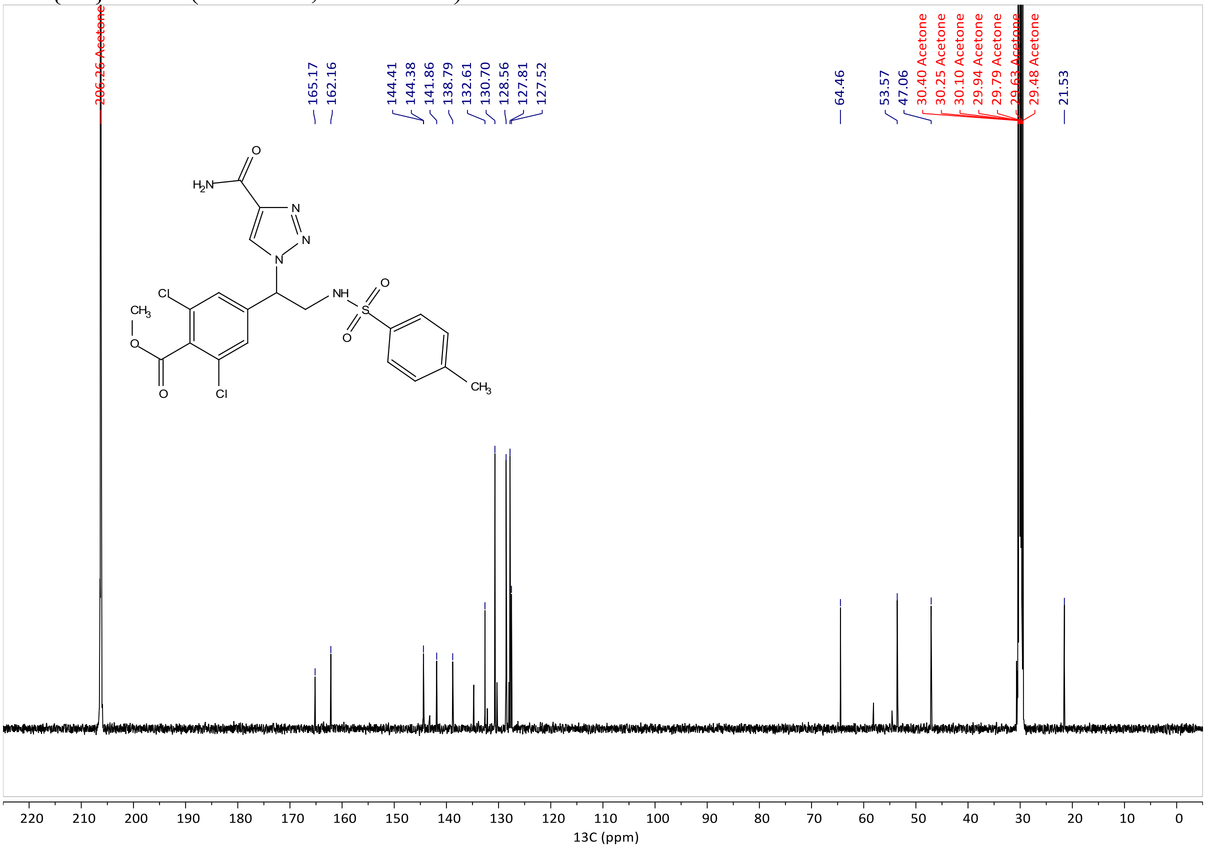

Supplement: Supplementary file 1 [file jo5c01782_si_001.pdf]
